# Apolipoprotein E4 has extensive conformational heterogeneity in lipid-free and lipid-bound forms

**DOI:** 10.1073/pnas.2215371120

**Published:** 2023-02-07

**Authors:** Melissa D. Stuchell-Brereton, Maxwell I. Zimmerman, Justin J. Miller, Upasana L. Mallimadugula, J. Jeremías Incicco, Debjit Roy, Louis G. Smith, Jasmine Cubuk, Berevan Baban, Gregory T. DeKoster, Carl Frieden, Gregory R. Bowman, Andrea Soranno

**Affiliations:** ^a^Department of Biochemistry and Molecular Biophysics, Washington University in St. Louis, Saint Louis, MO 63110; ^b^Center for Science and Engineering of Living Systems, Washington University in St. Louis, Saint Louis, MO 63130

**Keywords:** apolipoprotein E, Alzheimer’s disease, single-molecule FRET, protein folding

## Abstract

The ε4-allele variant of apolipoprotein E (ApoE4) is the strongest genetic risk factor for Alzheimer’s disease, although it only differs from its neutral counterpart ApoE3 by a single amino acid substitution. While ApoE4 influences the formation of plaques and neurofibrillary tangles, the structural determinants of pathogenicity remain undetermined due to limited structural information. Previous studies have led to conflicting models of the C-terminal region positioning with respect to the N-terminal domain across isoforms largely because the data are potentially confounded by the presence of heterogeneous oligomers. Here, we apply a combination of single-molecule spectroscopy and molecular dynamics simulations to construct an atomically detailed model of monomeric ApoE4 and probe the effect of lipid association. Importantly, our approach overcomes previous limitations by allowing us to work at picomolar concentrations where only the monomer is present. Our data reveal that ApoE4 is far more disordered and extended than previously thought and retains significant conformational heterogeneity after binding lipids. Comparing the proximity of the N- and C-terminal domains across the three major isoforms (ApoE4, ApoE3, and ApoE2) suggests that all maintain heterogeneous conformations in their monomeric form, with ApoE2 adopting a slightly more compact ensemble. Overall, these data provide a foundation for understanding how ApoE4 differs from nonpathogenic and protective variants of the protein.

## Significance

Despite being identified as the strongest genetic risk factor for Alzheimer’s disease more than 20 years ago, a connection between the biochemical properties of apolipoprotein E (ApoE) and its role in the disease remains elusive. This is largely due to the limited structural information available for the different forms adopted by the protein (monomer, dimer, tetramer, and lipid bound) across pathogenic and nonpathogenic variants. Here, we provide the characterization of the full-length pathogenic ApoE4 in its monomeric form both in the presence and absence of lipids. We demonstrate that the protein does not adopt a single structure, but a multiplicity of different conformations, which impacts the interpretation of the structure-function mechanism of ApoE.

Apolipoprotein E (ApoE) is a 299-amino acid protein involved in lipid transport and cholesterol homeostasis (1, 2) that plays a key role in Alzheimer’s disease (AD). The polymorphic nature of human APOE allows for encoding three variants (ApoE2, ApoE3, and ApoE4) (3) that have dramatic functional differences, even though it is only a single amino acid change that differentiates ApoE3 from ApoE2 (R158C) and ApoE4 (C112R) (4). The most striking example is ApoE4, which is recognized as the major genetic risk factor for AD (5–9), with individuals who are homozygous for the ε4 allele having up to 15-fold higher probability of developing late-onset AD (10, 11). In contrast, ApoE3 appears to have no impact on the progression of AD, while ApoE2 has been proposed to be protective toward the disease (12). A current hypothesis is that these functional differences stem from structural changes imposed upon ApoE by this single residue substitution and thus having a potential impact on its interaction with AD factors, such as amyloid-beta plaques and neurofibrillary tangles (13, 14). In both the cardiovascular and the central nervous systems, ApoE is prevalently associated noncovalently with lipids as part of lipoproteins, and the single residue substitutions are known to alter its interaction with specific lipoprotein populations (15). From a biochemical point of view, previous work from Garai et al. suggests that only the monomeric form—not the oligomers—is competent for high-affinity lipid binding (16). Therefore, understanding the monomeric structure of ApoE is key to unmasking the mechanisms controlling its interaction with lipids. In addition, recent experiments have found that ApoE expressed by microglia and astrocytes can also occur in poorly lipidated and nonlipidated forms (17). However, a structural characterization of monomeric ApoE in its lipid-free states remains elusive. One major obstacle is posed by the high propensity of ApoE to form oligomers (18), which hampers the investigation of the monomeric form (*SI Appendix*, Fig. S1). A second challenge is the disordered nature of numerous short segments of the protein, which have been proposed to be flexible and confer structural heterogeneity (19) rendering these regions invisible to conventional structural biology methods.

**Fig. 1.**
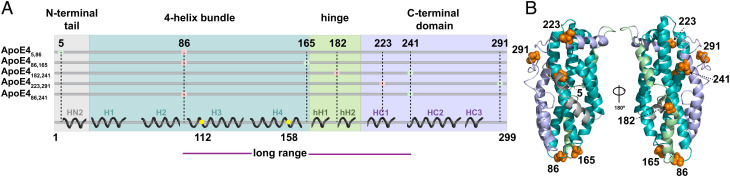
Protein structural regions and single-molecule constructs of full-length ApoE4. (*A*) Schematic representation of the secondary structure content in ApoE4 based on the NMR structure (Protein Data Bank (PDB): 2L7B) of the ApoE3-like variant with corresponding designations and identification of the major protein domains: N-terminal tail (gray), four-helix bundle (teal), hinge region (green), and C-terminal domain (light purple). Helical notations are reported for each helix. Labeling positions are identified on the linear sequence by green and red dots (the color scheme is only indicative of FRET labels and not of residue labeling for a specific fluorophore). Yellow dots identify the mutations associated with ApoE3 and ApoE2 variants. Position A86C is located in the random coil between helices H2 and H3 as previously defined (20, 30) and serves as a common reference point to investigate the folded N-terminal domain from two different perspectives. When paired with position A5C (ApoE4_5,86_), which is situated upstream of the start of the H1 helix, A86C monitors the conformational properties and folding stability of the N-terminal tail. When paired with position G165C (ApoE4_86,165_), which is located at the end of the H4 helix, A86C provides a readout for the folding of the four-helix bundle (22, 30). Positions G182C and A241C (ApoE4_182,241_) allow monitoring the behavior of the hinge domain with respect to the C terminus, while positions S223C and A291C (ApoE4_223,291_) provide information on the structural properties of the C-terminal domain. Finally, probe positions located at A86C and A241C (ApoE4_86,241_) allow us to monitor long-range interactions between the N- and C-terminal domains. (*B*) One hundred and eighty-degree rotated views of the monomeric ApoE3-like variant NMR structure (PDB: 2L7B) highlighting labeling positions shown in orange. Structure color differentiates the major protein domains described in *A*.

ApoE comprises four different regions: the N-terminal tail (residues 1 to 23), the four-helix bundle (24 to 167) (20–22), the hinge region (168 to 205), and the C-terminal domain (206 to 299) (Fig. 1). Current conformational models (19, 23) of the monomeric lipid-free ApoE agree on the structure of the four-helix bundle (20–22), but they disagree on the configurations of the hinge and C-terminal regions and their orientation with respect to the four-helix bundle. Ensemble Förster resonance energy transfer (FRET) and Electron Paramagnetic Resonance (EPR) studies (24) suggest that ApoE4 forms a close contact between the four-helix bundle and the C-terminal domain, whereas ApoE3 explores more open conformations. This is at odds with the compact set of structures determined by Nuclear Magnetic Resonance (NMR) on a monomeric ApoE3-like variant (22). Recent Hydrogen Deuterium Exchange Mass Spectrometry (HDX-MS) experiments identified isoform-dependent differences in solvent accessibility of the four-helix bundle, hinting that single amino acid substitutions affect the ability of the C-terminal domain to shield specific regions of the four-helix bundle (19). However, the interpretation of ensemble FRET, EPR (24), and HDX-MS experiments (19) is complicated by the fact that measurements were performed under conditions in which the protein is a stable tetramer (16, 19) and, therefore, are not representative of the conformations of the protein in its monomeric form. The same limitation applies to previous investigations of the folding stability of the protein domains (16, 25–27) and its interaction with lipids (24–29), where ApoE was studied at concentrations that favor either dimer or tetramer conformations (16, 24, 28, 29).

Here, we circumvent these experimental difficulties by harnessing single-molecule fluorescence spectroscopy, an approach that enables working at sufficiently low protein concentrations to avoid oligomerization and directly access the protein in its monomeric form (*SI Appendix*, Fig. S1). Single-molecule FRET provides a direct readout on the conformations and stability of specific domains within full-length ApoE4 in both the lipid-free and lipid-bound states. We further complement single-molecule observations with molecular dynamics (MD) simulations to obtain an atomically detailed representation of protein conformations that is consistent with our experimental data.

## Results

To study the conformations of ApoE4 via single-molecule FRET, we designed, expressed, and purified five distinct full-length double-cysteine mutants of the protein (Fig. 1*A* and *SI Appendix*). We used the ApoE3-like structure determined by NMR (22) (Fig. 1*B*) as a blueprint to guide our choice of labeling positions, such that each dye pair combination probes one of the four regions of the protein.

### Folding and Stability of the Four-Helix Bundle.

**Fig. 2.**
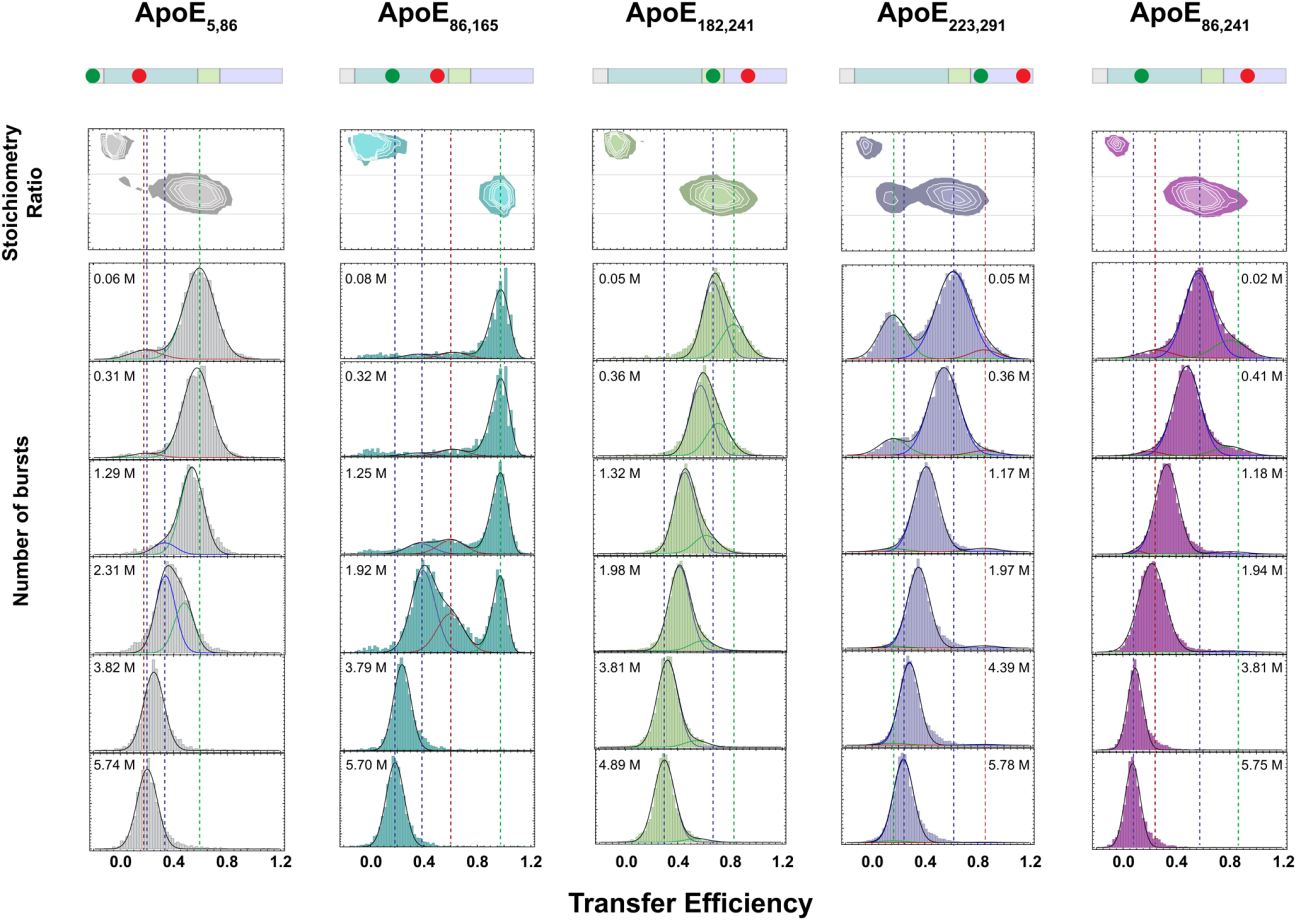
Single-molecule fluorescence experiments of lipid-free full-length ApoE4. Transfer efficiency histograms for selected bursts with fluorescence stoichiometry ratio between 0.3 and 0.7 across the five full-length constructs ApoE4_5,86_ (gray), ApoE4_86, 165_ (teal), ApoE4_182,241_ (green), ApoE_223,291_ (light purple), and ApoE4_86,241_ (magenta) at increasing concentrations of GdmCl. Under aqueous conditions, all histograms reveal coexistence of multiple states. Lines are visual guides for contrasting the native and completely unfolded configurations in each construct.

**Fig. 3.**
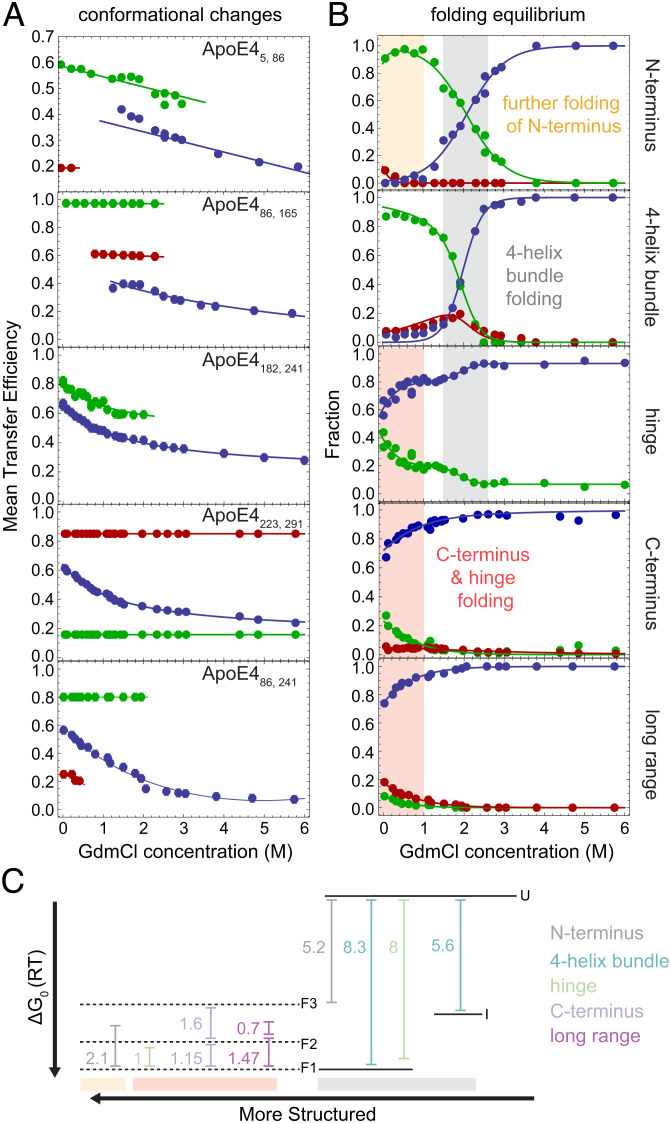
Mean transfer efficiencies and relative fractions of populations for lipid-free ApoE4. (*A*) Blue, red, and green identify corresponding populations in transfer efficiency histograms of Fig. 2. Solid lines connect mean transfer efficiencies to simply provide a visual guide. Mean transfer efficiencies are shown only for population fractions larger than 10% or when analyzed assuming a fix shared value. Associated SD errors are reported in *SI Appendix*, Table S1. (*B*) Solid lines reflect independent fits with a three-state equilibrium between the different conformers (*SI Appendix*, Table S2 and Fig. S2). Vertical shaded areas indicate folding across specific regions. (*C*) Free energy diagram of identified states in the four-helix bundle, hinge, and N and C termini and from long-range measurements. Solid lines represent the equilibrium between completely unfolded protein (U), formation of the intermediate (I), and complete folding of the four-helix bundle (F). Dashed lines indicate the different folded states identified in the N-terminal tail, hinge region, C-terminal domain, and long-range contacts. Dashed lines are used to underline that these different configurations coexist with the folded state of the four-helix bundle. See *SI Appendix*, Fig. S4 for free energy diagrams of each single construct.

We first focus on the ApoE4_86,165_ construct, where labeling positions are located in the random coil between helices H2 and H3 (A86C) and at the end of helix H4 (G165C), which enables probing the folding of the four-helix bundle. Although 79 amino acids apart in the sequence, the two labeling positions are expected to be in close proximity with a predicted transfer efficiency of 0.99 (Fig. 1*B*) based on the ApoE3-like NMR structure (22). Indeed, under aqueous buffer conditions (50 mM NaPi, pH 7.4), single-molecule FRET measurements of ApoE4_86,165_ display a narrow distribution of transfer efficiencies with a mean value of 0.98 ± 0.01 (Fig. 2 and *SI Appendix*, Table S1) compatible with the folded four-helix bundle. With increasing concentrations of guanidinium chloride (GdmCl) (Fig. 2), the fraction of the population at high transfer efficiency decreases in favor of two other populations characterized by distinct mean transfer efficiencies. One population is observed at E ~ 0.62 across different GdmCl concentrations, and its relative abundance exhibits a nonmonotonic trend, increasing between 0 and 1.5 M GdmCl and then decreasing until its disappearance at ~3 M GdmCl (Fig. 3*A*), which is consistent with an intermediate state. Its lower transfer efficiency, compared to the folded state, is compatible with a more expanded conformation (*SI Appendix*, Fig. S2), suggesting a partial unpacking of the four-helix bundle. The other population reveals a continuous shift in transfer efficiencies from 0.35 to 0.2 when moving from low to high denaturant concentration (Fig. 3*A*), which is accompanied by a continuous increase in its relative abundance (Fig. 3*B*). This is consistent with the behavior expected for an unfolded region undergoing denaturation (31). By fitting the relative abundance of each population with a three-state model, we quantify the stability of the intermediate and folded states, which are ΔG_0_^UI^ = −5.6 ± 0.4 RT and ΔG_0_^UF^ = −8.3 ± 0.4 RT, respectively (Fig. 3*C* and *SI Appendix*, Figs. S3 and S4 and Table S2), with R being the universal gas constant and T the room temperature. The midpoint of the unfolding transition occurs at ~2 M GdmCl (Fig. 3*B*), which is in excellent agreement with previous ensemble experiments (25–27) (*SI Appendix*, Table S3).

### N-Terminal Tail.

We complete the investigation of the N-terminal domain by focusing on the N-terminal tail, which is not resolved in the crystal structure of the four-helix bundle (30). Position A5C is situated upstream of the start of helix H1 and when paired with A86C monitors the conformational properties of the N-terminal tail (Fig. 1). Single-molecule FRET measurements of ApoE4_5,86_ reveal two distinct populations in equilibrium under aqueous buffer conditions. The more abundant population has a mean transfer efficiency of 0.61 ± 0.02, while the less abundant population sits at 0.21 ± 0.05 (Fig. 2). Comparing the donor lifetime vs. transfer efficiency indicates that the population at low transfer efficiency is compatible with a rigid distance where positions 5 and 86 are located ~7 nm apart (*SI Appendix*, Fig. S2). Conversely, the population at higher transfer efficiency follows the expected trend of a dynamic conformational ensemble, that is, an ensemble of interdye distances that are sampled in a timescale much shorter than the residence time of the protein in the confocal volume. Interestingly, the results are better described using a wormlike chain distribution with persistence length *l_p_* (an estimate of the minimal flexible segment) equal to 2.5 nm and contour length *l_c_* (the maximum extension of the probed region) equal to 7.7 nm (*SI Appendix*, Fig. S2). Note that this contour length is just ~25% of the contour length expected for an equivalent fully disordered region, suggesting that secondary structure formation occurs within this population. To further test for the presence of secondary structure, we investigated the effect of denaturant. We observe that the population at low transfer efficiency is completely destabilized at 0.5 M GdmCl and that the population at higher transfer efficiency tends to shift toward lower values with increasing denaturant (Fig. 3*A*). This result is consistent with a population that is not completely structured and contains a certain degree of flexibility (31). Interestingly, a noticeable shift in the mean transfer efficiency of this population occurs between 1 and 2 M GdmCl accompanied by a change in the width of the distribution (*SI Appendix*, Figs. S5 and S6). We interpret this behavior as the result of the coexistence of two populations with similar transfer efficiencies within the same observed peak. By fitting two independent populations within the mean transfer efficiency distribution (Fig. 2), we obtain a midpoint of the transition (c_1/2_) equal to 2.06 ± 0.01 M and a ΔG_0_ equal to 5.2 ± 0.2 RT (Fig. 3*C*, compare alternative analysis in *SI Appendix*). This observation can be understood considering that positions 5 and 86 sample not only the N-terminal tail but also helices H1 and H2 of the four-helix bundle.

### Hinge Region.

Positions G182C and A241C (ApoE4_182,241_) allow monitoring of the behavior of the hinge domain with respect to the C terminus. Analysis of the corresponding transfer efficiency histograms reveals an asymmetric distribution of transfer efficiencies under aqueous buffer conditions. We analyze the asymmetric distribution in terms of two distinct populations (Fig. 2). The population associated with lower mean transfer efficiency (E = 0.62 ± 0.02) accounts for 60% of the observed molecules, whereas the high transfer efficiency population (E = 0.83 ± 0.02) accounts for the remaining 40%, corresponding to a free energy difference between these states of 1.0 ± 0.2 RT (*SI Appendix*, Tables S1 and S2). The asymmetry of the distribution persists with increasing denaturant concentrations, with both populations shifting toward lower transfer efficiencies (Fig. 3), as expected for disordered or partially disordered regions (31). Comparing lifetime and transfer efficiency indicates that both populations reflect dynamic averages that, similar to the case of the N-terminal tail, we can describe in terms of a wormlike chain (*SI Appendix*, Fig. S2). Interestingly, the dependence of the relative abundance of the two populations on denaturant concentration reveals a second transition in the range between 1.5 and 2.5 M GdmCl concentration. The range of this transition coincides with the same range observed for the folding transition of the four-helix bundle (c_1/2_ = 1.9 ± 0.2 M, *SI Appendix*, Table S2) and suggests a conformational change of the hinge region concomitant with the folding of the N-terminal domain.

### C-Terminal Domain.

Positions S223C and A291C (ApoE4_223,291_) provide information on the structural properties of the C-terminal domain. Under aqueous buffer conditions, we observe a broad distribution of transfer efficiencies that correspond to at least three distinct conformational states sampling long-, middle-, and short-range distances between the fluorophores (Fig. 2). When comparing donor lifetime and transfer efficiency, the population at 0.13 ± 0.04 mean transfer efficiency is compatible with a rigid region of ~7.9 nm (*SI Appendix*, Fig. S2). This population accounts for 27 ± 4% of the protein configurations and is completely destabilized in favor of the other populations above 1.2 M GdmCl. Different is the case for the population with transfer efficiency equal to 0.61 ± 0.02, whose donor lifetime follows the expected trend for a dynamic ensemble and whose relative abundance is stabilized by increasing concentrations of denaturant. Both elements point toward a population that is more flexible and, at least, partially disordered, as further supported by the continuous shift of the peak from high to low transfer efficiencies when tuning the solvent quality from a poorer solvent (aqueous buffer) to a better solvent (GdmCl). The increased broadening of the width of this population below 1 M GdmCl (*SI Appendix*, Fig. S5), which exceeds the width measured for other constructs, points to an increased heterogeneity due to structure formation. This is consistent with previous characterizations of the C-terminal region, where destabilization of the secondary structure was observed above 1 M GdmCl (25, 26). The third population at ~0.85 mean transfer efficiency represents more compact configurations of the C-terminal domain, where positions 223 and 291 are brought in close proximity. Interestingly, the small relative abundance of this population decreases above 1 M GdmCl and disappears at 2.75 M GdmCl (Fig. 3). This regime of concentrations coincides with the folding of the four-helix bundle and mirrors that observed for the hinge region, suggesting that folding of the four-helix bundle induces conformational changes in the C-terminal region.

### Proximity of the N- and C-Terminal Domains.

To better understand whether the four-helix bundle and the C-terminal region form stable contacts and to which extent they are brought in close proximity, we investigate the transfer efficiency distribution between A86C and A241C (ApoE4_86,241_). Under aqueous buffer conditions, we observe the occurrence of at least three populations with corresponding mean transfer efficiencies of 0.24 ± 0.01, 0.59 ± 0.02, and 0.87 ± 0.02 (Fig. 2). This is consistent with observation of multiple configurations in both the hinge and C-terminal regions. When comparing donor lifetime and transfer efficiency (*SI Appendix*, Fig. S2), all these populations lie on the trend expected for a dynamic ensemble, excluding the formation of stable contacts that would give rise to rigid configurations of the protein. Interestingly, a small percentage of the collapsed state, represented by the high transfer efficiency population, persists up to concentrations of denaturant that are compatible with the unfolding of the N-terminal domain. This implies the formation of a small fraction of more compact configurations of the protein that, nevertheless, retain a dynamic nature.

### MD Simulations Confirm Structural Heterogeneity.

**Fig. 4.**
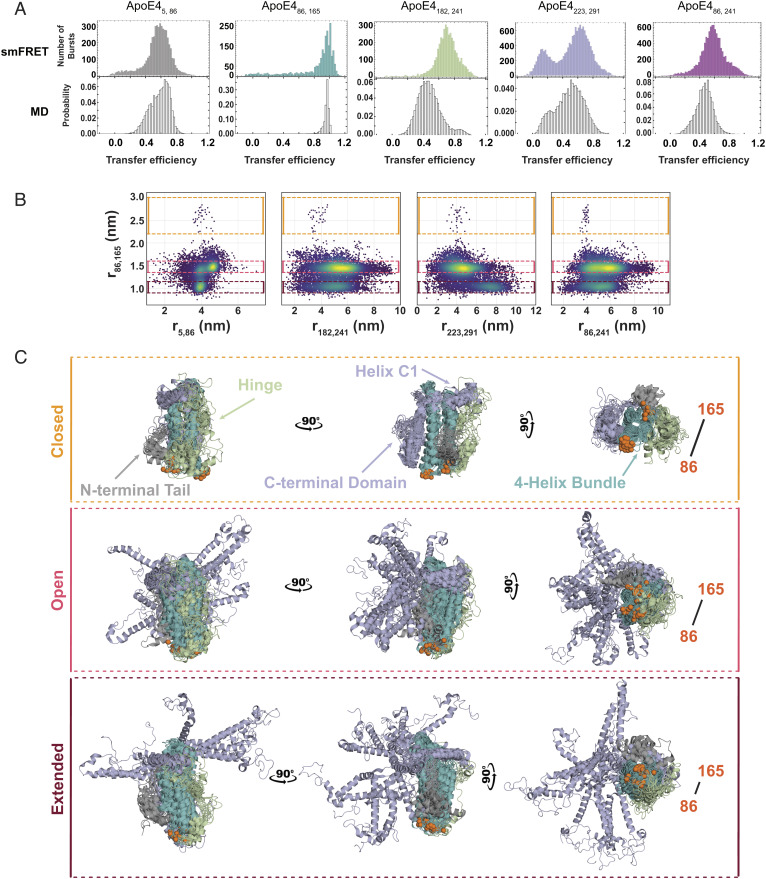
Comparison between transfer efficiency histograms in single-molecule measurements and MD simulations for lipid-free ApoE4. (*A*) Single-molecule FRET histograms of the five investigated constructs ApoE4_5, 86_ (gray), ApoE4_86, 165_ (teal), ApoE4_182, 241_ (green), ApoE4_223, 291_ (light purple), and ApoE4_86, 241_ (purple) are compared with equivalent distribution of transfer efficiencies computed from MD simulations (white). (*B*) Distance pair correlations from MD simulations contrasting the distance r_86, 165_ with the distances r_5,86,_ r_182,241_ and r_223,291,_ r_86,241._ Colored boxes (yellow, red, and brown) identify three major configuration regimes of the four-helix bundle and corresponding changes in the other protein regions. (*C*) The 15 most probable configurations for each of the three states *closed*, *open*, and *extended*, as identified from the data in panel *B*. Position of 86 and 165 fluorophores is highlighted in orange, whereas the N-terminal tail is displayed in gray, the four-helix bundle in teal, the hinge region in green, and the C-terminal domain in light purple (compare with Fig. 1).

**Fig. 5.**
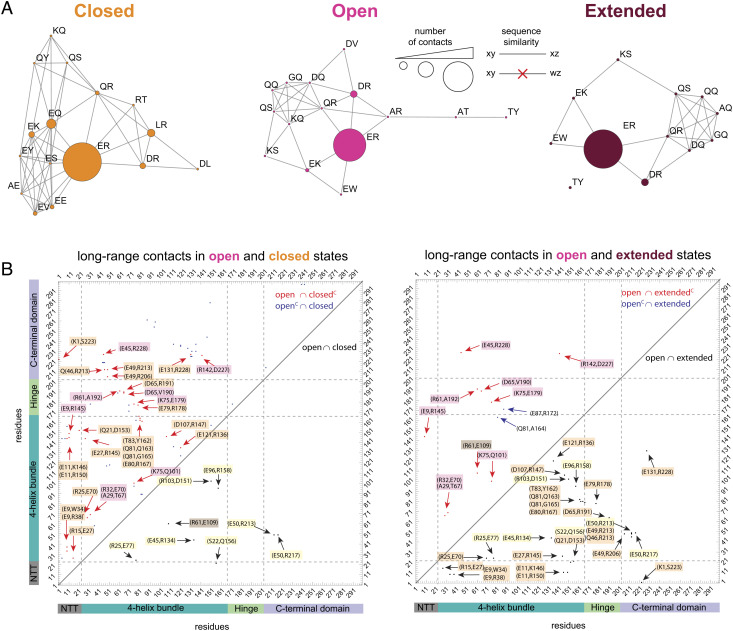
Long-range contact differences across the *closed*, *open*, and *extended* subpopulations of ApoE4. Long-range contacts here are identified residues whose centers of mass are less than 3 Å apart from each other and that are separated in sequence by at least six residues. (*A*) Interacting residues identified in the *closed*, *open*, and *extended* subpopulations. Letters represent amino acid codes. Nodes are scaled according to the number of contacts, and edges connect contacts based on sequence similarity. The majority of contacts occurs between charged residues (e.g., glutamic acid and arginine). (*B*) List of long-range contacts. *Left*: contacts that are in the *open* and *closed* configurations (*open* ⋂ *closed*, black), contacts that are in the *open* but not in the *closed* configuration (*open* ⋂ *closed*^*C*^, red), and contacts that are in the *closed* but not in the *open* configuration (*open*^C^ ⋂ *closed*, blue). *Right*: contacts that are in the *open* and *extended* configurations (*open* ⋂ *extended*, black), contacts that are in the *open* but not in the *extended* (*open* ⋂ *extended*^C^, red), and contacts that are in the *extended* but not in the *open* (*open*^C^ ⋂ *extended*, blue). Highlighted in yellow: contacts that are shared across all three states (*closed* ⋂ *open* ⋂ *extended*). Highlighted in orange: contacts that are in the *open* and *extended* but not in the *closed* configuration (*open* ⋂ *extended* ⋂ *closed*^C^). Highlighted in red: contacts that are in the *open* but not in the *extended* and *closed* configurations (*open* ⋂ *extended*^C^ ⋂ *closed*^C^). Highlighted in brown: contacts that are in both *open* and *closed* but not in the *extended* configuration (*open* ⋂ *closed* ⋂ *extended*^C^).

To gain insights into the structural details of the conformational ensemble of ApoE4, we performed all-atom MD simulations of the full-length protein on the distributed computing platform Folding@home for a total aggregated time of 3.45 ms. We then constructed a Markov state model to bin the conformational ensemble into unique states. For each observed state, we modeled fluorophores onto the labeling positions post hoc and reconstructed a set of transfer efficiency histograms that accounts for shot noise and the kinetic averaging of conformations in the observation timescale (*SI Appendix*). The comparison between simulated and measured transfer efficiency histograms is shown in Fig. 4*A*. We find good agreement between both datasets, including the occurrence of a multimodal transfer efficiency distribution for ApoE_223,291**,**_which was not captured in microsecond-long simulations, stressing the importance of an extensive sampling of the energy landscape with long simulation times (*SI Appendix*, Fig. S7). Deviations in the mean transfer efficiencies and relative abundance of populations are within experimental errors and known limits of comparing these approaches (*SI Appendix* and *SI Appendix*, Fig. S8). To better disentangle the conformations underlying the simulated transfer efficiency histograms, we analyzed the simulation data for the occurrence of correlations across all distance pairs (Fig. 4*B* and *SI Appendix*, Figs. S9–S12). This analysis reveals three subpopulations associated with the distance between positions 86 and 165 whose mean transfer efficiencies fall within the observed distribution for ApoE_86,165._ The conformational changes in these subpopulations are not restricted to these specific labeling positions but propagate across the entire protein, highlighting correlated changes in the hinge region and anticorrelated ones in the C-terminal domain. In particular, the identified subpopulations in each distance pair correlation parallel the distance and relative abundance trends observed in the experiments. All three identified subpopulations differ from the ApoE3-like NMR structure, where numerous contacts previously identified between the four-helix bundle and the C-terminal domain are not observed even in the more compact conformations (*SI Appendix*, Fig. S10). Alignment of subpopulation structures reveals how these correlative trends reflect different degrees of conformational heterogeneity in the protein (Fig. 4*C*). We refer to the three major subpopulations as *closed*, where the C-terminal domain is docked on the four-helix bundle, *open*, where the C-terminal domain is undocked, and *extended*, where the undocked C-terminal domain adopts more extended configurations. Interestingly, these conformational differences do not stem from varying degrees of secondary structure in the C-terminal domain (*SI Appendix*, Fig. S11). We further analyzed the simulations to verify whether specific residue contacts are maintained despite the extensive conformational heterogeneity. We identified a set of persistent contacts within the four-helix bundle and two additional contacts between the four-helix bundle and the HC1 helix of the C-terminal domain, which suggests that the relative position of HC1 with respect to the four-helix bundle is maintained across all the subpopulations (Fig. 5). At variance with the *closed* subpopulation, the *open* and *extended* ensembles show an increase in the number of contacts of the N-terminal tail with the four-helix bundle and the HC1 helix, which may dictate whether the C-terminal domain docks onto the four-helix bundle. Interestingly, there are no shared contacts across the three subpopulations within the N-terminal tail or the hinge region (Fig. 5, highlighted in yellow), which suggests that these regions are adopting different conformations in each state. Indeed, the position of the hinge region differs across the three subpopulations and is directed by interactions between the hH1 helix and either the N-terminal tail or the four-helix bundle (Fig. 5). Specifically, in the *closed* configuration, the hinge region mainly interacts with helices H1 and H2, whereas in the *open* and *extended* configurations, the hinge explores the surface of helices H2 and H3 with differing extent of specificity. Altogether, MD simulations confirm the experimental observation that lipid-free ApoE4 adopts a dynamic structural ensemble with at least three distinct states.

### Conformational Heterogeneity Is Maintained across Isoforms.

We then turn to investigate whether mutations at residue 112 (as in ApoE3) and at residues 112 and 158 (as in ApoE2) alter the proximity of the four-helix bundle and C-terminal region of the protein, as previously proposed (20, 21, 32), or even suppress conformational heterogeneity, as observed in the ApoE3-like NMR structure (22). To this end, we create two constructs ApoE3*_86,241_ and ApoE2*_86,241_ where we insert serine residues in either position 112 or both positions 112 and 158. Serine residues are chosen because they do not interfere with the maleimide chemistry labeling. Importantly, the substitution Arg112Ser is known to replicate the effects of ApoE3 (Arg112Cys), with similar reduced domain interaction (33), lipid- (34) and lipopolysaccharide-binding properties (35), and formation of SDS-resistant complex with A*β* that is unique to apoE3 (36).

**Fig. 6.**
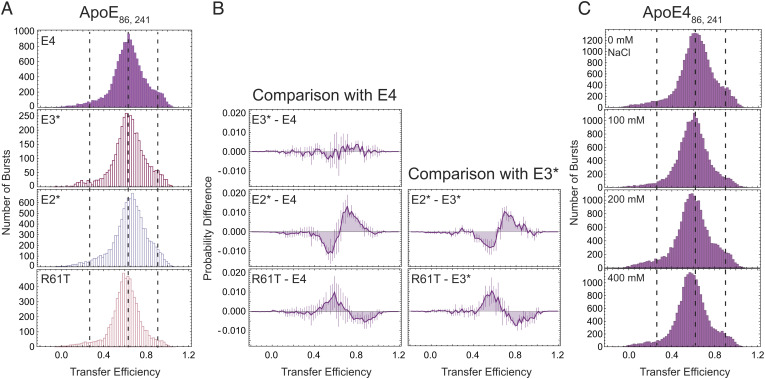
Single-molecule FRET experiments comparing effects of single point mutations on long-range conformations. (*A*) Comparison of transfer efficiency histograms for the four different ApoE variants (ApoE4, ApoE3*, ApoE2*, and ApoE4_R61T_) across the distance 86 and 241. Dashed lines are guides for the eyes to the populations observed in ApoE4. (*B*) Comparison of the normalized probability distributions of transfer efficiencies of each variant against the one of ApoE4 (first column) or ApoE3* (second column). Errors are propagation of SDs from three independent measurements of each distribution. No significative deviation between ApoE3* and ApoE4 distributions is observed; however, ApoE2* displays a small but detectable shift toward higher transfer efficiencies when compared to ApoE3* and ApoE4, whereas the R61T mutation introduces a small shift toward lower transfer efficiencies compared to E3* and E4. (*C*) Salt titration reveals a shift toward lower transfer efficiencies with increasing NaCl concentration, indicating an expansion of the long-range distance between 86 and 241 upon screening of electrostatic interactions.

In our single-molecule FRET experiments, we found that all three constructs exhibit three distinct populations, indicating that conformational heterogeneity is maintained across the isoforms (Fig. 6*A*). Comparison of lifetime vs. transfer efficiency confirms the dynamic nature of these states. While ApoE4_86,241_ and ApoE3*_86,241_ exhibit similar mean transfer efficiencies, ApoE2*_86,241_ shows a minor shift toward higher mean transfer efficiencies for the population with ~0.6 transfer efficiency (Fig. 6*B*). All three isoforms are measured from the perspective of the same interdye distance between the N- and C-terminal regions, and therefore, we conclude that ApoE2* adopts slightly more compact conformations than ApoE3* and ApoE4.

### Testing Salt Bridge Formation.

Previous experiments proposed a close proximity of residues 76 and 241 (24) in ApoE4 that is helped by a salt bridge formation between residues 61 and 255 (15, 30). Such a close configuration is expected to be altered in ApoE3, leading to more extended configurations. While we did not find the salt bridge in the simulations (*SI Appendix*, Fig. S10) and we did not observe significant changes in the distribution of transfer efficiencies for ApoE3* and ApoE4, we further tested this hypothesis by introducing a R61T mutation in ApoE4, ${\text{ApoE4}}_{86,241}^{R61T}$, which suppresses the putative salt bridge formation between 61 and 255. As shown in Fig. 6 *A* and *B*, we do observe a minimal shift toward more expanded conformations. The expansion pertains to the population with a transfer efficiency of ~0.6 and is associated with an average distance of about 5 nm. Given our labeling positions are near residues 76 and 241, our observations suggest that 76 and 241 are in close proximity for only a small fraction of sampled configurations (represented by the high transfer efficiency shoulder). If a stable close configuration of the protein was formed upon salt bridge formation, we would expect to observe a clear change in the relative fractions of each population when suppressing salt bridge formation. Interestingly, the R61T mutation only minimally decreases the fraction of molecules associated with the high transfer efficiency population, suggesting that this population does not represent a salt bridge–dependent conformation. Altogether, our results suggest that, in the monomeric form, the R61T mutation does not introduce significant changes when compared to ApoE3* and ApoE4.

### Contribution of Electrostatic Screening.

Given the large proportion of surface-exposed charged residues within the N- and C-terminal regions, we further tested the effect of salt on modulating electrostatic contribution to the conformational ensemble of the protein. Titration of increasing concentrations of NaCl on ApoE4_86,241_ does not significantly alter the proportion of the relative fractions (Fig. 6*C*), implying that the interactions between the four-helix bundle and the C-terminal domain are not exclusively of electrostatic nature. However, the mean transfer efficiency associated with the major population shifts toward lower values, indicating an expansion of the conformational ensemble upon ion screening of the electrostatic interactions. This suggests that salt concentration can modulate the distal organization of ApoE domains but does not alter the equilibrium between the three major identified states.

### Lipid Association of ApoE4.

**Fig. 7.**
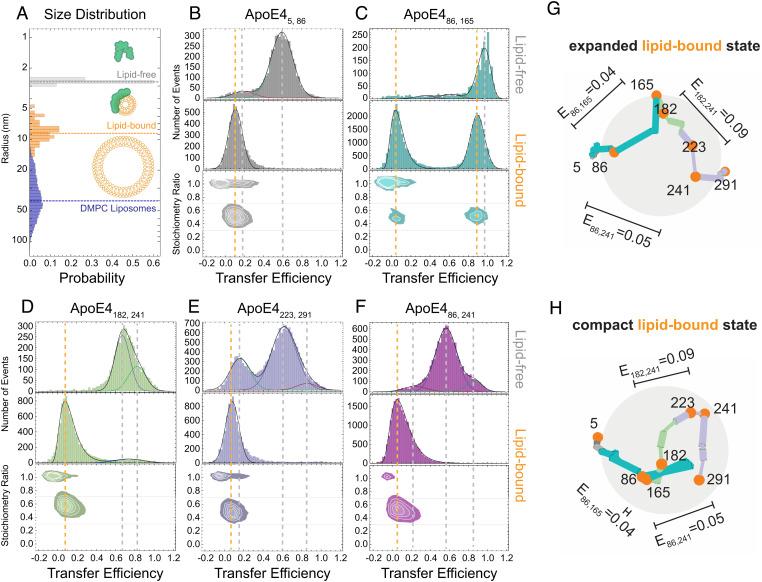
Single-molecule fluorescence experiments on lipid-bound ApoE4. (*A*) Distribution of radii for lipid-free ApoE4 (gray, df-FCS), lipid-bound ApoE4 (orange, FCS), and extruded DMPC liposomes (blue, cryo-TEM). (*B*–*F*) Comparison of transfer efficiency histograms for lipid-free and lipid-bound ApoE4 constructs. All histograms report on fluorescent species with a labeling stoichiometry ratio of 1D:1A. Lines are visual guides between the lipid-free (gray) and lipid-bound (orange) mean transfer efficiencies. (*G*) and (*H*) Representatives examples of conformations of ApoE4 in the expanded and compact lipid-bound states based on an ultracoarse-grained model that satisfies the mean transfer efficiency constraints.

Finally, we turn to investigating how the structural heterogeneity of ApoE4 is impacted by binding to lipids, which reflects the most likely populated configuration under physiological conditions. To this end, we focus on the interaction between the ApoE4 constructs and dipalmitoylphosphatidylcholine (DMPC) liposomes with an average radius of 40 ± 20 nm (Fig. 7*A* and *SI Appendix*, Fig. S13). We chose DMPC because it is a good mimic of the lipids found in lipoproteins both in terms of hydrophilic head group and average length of the fatty acid chain (37, 38). Using single-molecule FRET and a high concentration of liposomes (100 µg/mL), we tested whether the labeled constructs could bind to lipids. ApoE4_5,86_(N-terminal tail), ApoE4_223,291_(C-terminal domain), and ApoE4_86,241_(long-range contacts) all exhibit a single narrow distribution of transfer efficiencies with a clear shift of the mean toward values lower than 0.2, representing very extended states of the protein (Fig. 7 *B*, *E*, and *F* and *SI Appendix*, Fig. S14). The complete disappearance of the populations observed for lipid-free ApoE4 confirms that these three constructs are fully associated with lipids. Interestingly, the construct ApoE4_86,165_(four-helix bundle) exhibits two coexisting populations in equilibrium, one at high transfer efficiency (0.894 ± 0.004) and one at low transfer efficiency (0.037 ± 0.006) (Fig. 7*C* and *SI Appendix*, Table S4). Neither transfer efficiency is compatible with the population measured in aqueous conditions in the absence of lipids. This suggests that the four-helix bundle can undergo unpacking and restructuring when associated with lipids and that a certain degree of heterogeneity, represented by these two distributions of transfer efficiencies, is conserved even in the lipid-bound state (Fig. 7 *G* and *H*). Finally, the ApoE4_182,241_(hinge region) construct also supports the occurrence of at least two distinct configurations of ApoE4 in the lipid-bound state (Fig. 7*D*), although the relative ratio between the two bound states is different compared to that of ApoE4_86,165_. This observation further reflects how the hinge and N-terminal domains are interconnected regions that maintain a certain degree of independence. Overall, taken together, these data support that the protein is completely associated with lipids at the studied concentration. We further analyzed the change in the fluorescence stoichiometry ratio of the lipid-bound vs. lipid-free conformations for each construct and validate that we are observing one single protein per the lipid-bound state. Binding of multiple proteins in the lipid-bound state would result in a significant change in stoichiometry since a nonnegligible fraction of molecules is double-labeled with only acceptor or donor fluorophores. The negligible variation in fluorescence stoichiometry suggests that the protein is monomeric (*SI Appendix*, Fig. S15). Finally, we observed that the low transfer efficiencies measured for lipid-bound ApoE correspond to relatively short distances (<10 nm) (*SI Appendix*, Figs. S14 and S16) when compared with the liposome size, posing the question on whether the protein is bound to the liposome or some portion of the liposome. Correlating the fluorescence signal (either from donor or acceptor direct excitation) in the same single-molecule measurements, we quantified the size of the lipid-bound states. The measurements clearly reveal an increase in the hydrodynamic radius of approximately two to three times the dimension of the lipid-free protein (*SI Appendix*, Figs. S17 and S18), which has no overlap with the liposome distribution. Overall, this suggests that during the interaction with liposomes, the protein not only undergoes a partial refolding of its domains but does also extract lipids from the larger liposomes in order to create smaller lipid–protein particles (Fig. 7).

## Discussion

### Conformational Heterogeneity in Lipid-Free ApoE4.

Our single-molecule experiments and MD simulations clearly reveal that ApoE4 does not adopt a single structure but, instead, explore a complex and dynamic conformational ensemble. Using the ApoE3-like structure as a reference (22), we observe large deviations in the conformations of the hinge and C-terminal domains of the protein and dynamic fluctuations in the four-helix bundle (Fig. 4*C*). Interestingly, we do not find evidence in the experiments and simulations of previously proposed contacts between residues 76 and 241 (24) or residues 61 and 255 (15, 30) (Fig. 5 and *SI Appendix*, Figs. S19 and S20), and our experimental and computational data agree with the orientation of the N- and C-terminal domains observed in the ApoE3-like structure (22). This discrepancy with previous data can be rationalized by noting that experiments that identified these close contacts (24) were performed under conditions where the protein exists as a dimer or tetramer and therefore may be specific only to these forms of the protein. Similarly, salt bridges (15, 30) have been tested via mutational analysis in the context of lipoproteins or nonmonomeric forms of the protein and may reflect other interactions at play in those specific forms, which either do not occur or rarely occur in the monomeric case. While capturing a similar orientation of the domains, our data are at variance also with the “closed” NMR structure (22) (*SI Appendix*, Figs. S10 and S19). This observation supports that mutations along the sequence, as the ones used to monomerize ApoE3 in the NMR experiments, may alter the delicate balance between specific conformers in the structural ensemble. Indeed, the simulations suggest that the hinge region competes with the C-terminal domain for interactions with the four-helix bundle, where specific contacts involving the N-terminal tail and the four-helix bundle can sway the preference of interaction for one region or the other (Fig. 6). Study of mutations in positions 112 and 158 reveals that long-range conformations in ApoE3* resemble the one observed in ApoE4, whereas ApoE2* adopts slightly more compact configurations. This result differs from what may be expected based on the functional differences previously described for each isoform. However, our experiments only probed one long-range distance within the protein; therefore, we cannot exclude that local regions of the protein or even other long-range distances are not affected by the same mutations. In addition, although the cysteine-to-serine mutations were previously shown to not influence the function of ApoE isoforms (33–36), these amino acid substitutions may also introduce local and global conformational changes. Finally, the comparison between our work and previous observations points to a key role of oligomerization in modulating the protein conformational ensemble. These three aspects will be investigated in future works.

### Folding Equilibrium of Lipid-Free ApoE4.

Our single-molecule experiments also enable a direct quantification of the stability associated with each conformer of the monomeric protein and provide insights on the overall folding reaction. The denaturant titration suggests that structuring of the N-terminal domain proceeds from a completely unfolded state through an intermediate state where helices H1 to H4 are partially formed, followed by the subsequent packing and stabilization of the bundle (Fig. 3*C*). Observation of an intermediate configuration in the four-helix bundle confirms previous interpretation of ensemble data where an intermediate state was presumed (26). Contextually to the folding of the four-helix bundle, a perturbation occurs in the configurations of the hinge and in the N- and C-terminal tails. While folding of these domains remains largely independent, our data suggest that their structural organization is not disconnected. Indeed, even for labeling positions that do not sample the four-helix bundle, we identify transitions with a midpoint at approximately 2 M GdmCl accompanied by a similar change in free energy (from 5 to 7 RT, Fig. 3*C* and *SI Appendix*, Figs. S3 and S4). While folding of the C-terminal region is only captured by broadening of the distribution of transfer efficiencies (*SI Appendix*, Fig. S5), the observation of distinct populations in the N- and C-terminal tails and hinge region provides quantification of the energy difference between these distinct states. The similarity in the relative populations between the hinge and C-terminal regions (as measured by ApoE4_182,241_ and ApoE4_223,291_) across different denaturant concentrations and the overlap between the sequence of the two regions suggest we are monitoring the same configurational change. Therefore, the emerging picture is of a folded four-helix bundle in equilibrium with at least three distinct populations of the C-terminal domain: *closed*, *open*, and *extended*. These three distinct configurations of the C-terminal domain are characterized by internal dynamics on the hundreds of nanosecond timescale and are in a slow exchange, one with each other, on a timescale longer than milliseconds (*SI Appendix*, Fig. S21).

### Monomeric ApoE4 Forms Heterogeneous Complexes with Lipids.

Early EPR studies of ApoE4 suggested that helices in the N- and C-terminal domains remain in close contact in the lipid-bound state, whereas the four-helix bundle undergoes structural rearrangements (28). A competing model proposed that lipid binding favors a separation between the N- and C-terminal halves of the protein based on the ApoE3-like NMR structure (19, 22). Interestingly, our data indicate that such an open configuration is a constitutive state explored by the ApoE4 monomer and, therefore, does not require interaction with the lipids to occur. The *open* and *extended* configurations expose the required surface of the C-terminal domain making interaction with lipids possible (*SI Appendix*, Fig. S11). Indeed, the region between positions 165 and 270 has been identified as containing Class A amphipathic helices, which can promote lipid binding (39). Therefore, modulation of the abundance of the open state may impact the affinity of ApoE variants for lipids. Our measurements further indicate that monomeric ApoE can extract lipids and form smaller particles compared to the initial liposome preparation. This observation is compatible with previous measurements monitoring decrease of turbidity in liposome solutions upon addition of ApoE (40–42). The ability to extract lipids implies an intercalation of the amphipathic helices of the protein within the lipid bilayer. Indeed, amphipathic helices are known to play a key role in nonenzymatic membrane fission (43), where the membrane fission can be self-propelled by insertion of a first helix that favors insertion of subsequent helices (44–46). This same mechanism may be at play in the interaction of ApoE with liposomes, where insertion of the C terminus can then propagate through the hinge to the N terminus (22). This model explains how the hinge region, which locks the N-terminal domain in the four-helix bundle structure, can be displaced, leading to a rearrangement of the helices of the bundle and allowing for more expanded configurations. Our experiments indicate that the N-terminal domain adopts at least two different configurations, one where the helices H3 and H4 are in close proximity to one another and one in which the four helices are spread apart on the lipid particle (Fig. 7 *G* and *H*). This interpretation is fully compatible with the configurations identified by Henry et al. (29) using cross-linking, mass spectrometry, and simulations of ApoE4, although our data suggest a more expanded configuration of the N-terminal tail (as measured by ApoE4_5,86_) and a larger separation between the N- and C-terminal halves of the protein (as measured by ApoE4_86,241_). Interestingly, previous simulations of ApoE3 identify only a close configuration for helices H3 and H4, possibly suggesting a different structural organization of the two variants in their monomeric lipid-bound form (47). Future work will address the local organization of each ApoE region to test whether different isoforms adopt unique configurations in the lipid-bound state.

## Conclusions

The realization that ApoE isoforms do not adopt one single stable structure but an intricate conformational ensemble opens the door to new explanations for the mechanism of function of the protein and its role in the context of AD. Our results demonstrate the potential of single-molecule approaches for investigating the relationship between structural ensemble and function of monomeric ApoE. This approach bypasses experimental complications due to protein oligomerization, setting the stage for exploring the impact of sequence variations and interaction with AD factors. Understanding how and why sequence mutations and environmental factors tune ApoE from being a risk factor to having neutral effects is key to identifying appropriate therapeutic strategies that can slow down or even arrest the progression of AD.

## Materials and Methods

### Protein Expression, Purification, and Labeling.

All ApoE4 constructs were expressed in BL21-Gold (DE3) cells (Agilent). The thioredoxin–His_6_–ApoE protein fusion was purified using a HisTrap FF column (Cytiva). The tag was cleaved by Human rhinovirus (HRV) 3C protease and separated from ApoE4 using a heparin Sepharose FF column (Cytiva). Anion exchange chromatography (Q Sepharose HP FF column, Cytiva) was then used as the final polishing step. Correct mass of the constructs was analyzed using sodium dodecyl sulfate–polyacrylamide gel electrophoresis (SDS-PAGE) and/or electrospray ionization mass spectrometry. All constructs have been labeled with Alexa 488 and Alexa 594, which serve as donor and acceptor, respectively. For further details, see *SI Appendix*.

### Single-Molecule Measurements.

All single-molecule fluorescence measurements were performed on a Picoquant MT200 instrument (Picoquant). Single-molecule FRET and fluorescence correlation spectroscopy (FCS) were performed with labeled protein concentrations of 100 pM estimated from dilutions of samples with known concentration based on absorbance measurements. All single-molecule measurements were performed in 50 mM NaPi, pH 7.4, 200 mM β-mercaptoethanol (for photoprotection), 0.001% Tween 20 (for surface passivation), and GdmCl at the reported concentrations, at a room temperature of 295 ± 0.5 K. Pulsed interleaved excitation was used to ensure that each burst represents the transfer efficiency determined from a 1:1 donor:acceptor stoichiometry. Importantly, attachment of the probes across different labeling positions has a small impact on the overall protein conformations as measured by dual-focus FCS, which reveals variations across the different constructs of less than 10%. All data were analyzed using the Mathematica package “Fretica” (https://schuler.bioc.uzh.ch/wp-content/uploads/2020/09/Fretica20200915.zip) developed by Daniel Nettels and Ben Schuler. Fluorescence lifetimes (*SI Appendix*, Fig. S22) are analyzed using a convolution with the instrument response function (*SI Appendix*, Fig. S23). Comparing transfer efficiency estimates from donor lifetimes (reporting about the nanosecond timescale) and from bursts of photons (reporting on the millisecond timescale) enables distinguishing whether the associated population represents a rigid configuration or a dynamic ensemble. In the case of a rigid configuration, the same transfer efficiency is recovered on both timescales and results in a constant value that follows the linear dependence of the lifetime on the mean transfer efficiency. In the case of a dynamic ensemble, a deviation from the linear dependence occurs, which depends on the sampled conformational distribution (31). Burst variance analysis (48) and nanosecond FCS (49) further provide information on interdye dynamics (*SI Appendix*, Fig. S21). For further details, see *SI Appendix*.

### MD Simulations.

The NMR structure of ApoE3 (Protein Data Bank (PDB) ID: 2L7B) was used as a starting point for our simulations, with mutations performed in PyMOL to achieve the structures of ApoE4. We performed 20 rounds of directed sampling harnessing the FAST algorithm (50) to explore the conformational space of ApoE4 using the residue pairs: R92 and S263, G182 and A241, and S223 and A291, as a directed metric. The resulting simulations were clustered with similar simulations of ApoE2, ApoE3, and ApoE3ChristChurch (R136S) to a shared state space with rmsd of 3.5 Å into a total of 18,182 structures that represented the diversity of states explored in our simulations. Each structure was solvated in a dodecahedron box with edges 1.0 nm longer than the largest structure observed in our FAST simulations. Subsequent simulations were launched from these states on the distributed computing platform, Folding@home with five independent simulations starting from each state. Each trajectory ran for a maximum of 100 ns, in total reaching an aggregate time of 3.45 ms. Simulations were clustered using distance-based clustering for 15 residue pairs distributed throughout ApoE (5 FRET pairs plus 10 additional residue pairs, *SI Appendix*, Table S15). The Markov state model was subsequently generated using a lag time of 10 ns and enspara’s MSMBuilder. Simulations were performed using the Amber03 force field in combination with the TIP3P water model. FAST simulations were performed using GROMACS, and Folding@home simulations were performed using OpenMM. FRET histograms were calculated using the smFRET tool deployed in enspara using a rescaling time factor of 225 (*SI Appendix*, Fig. S24). For further details, see *SI Appendix*.

## Supplementary Material

Appendix 01 (PDF)

## Data Availability

The code for the kinetic Monte Carlo simulation of photon trajectories has been developed as a command line app and is distributed via the enspara GitHub (https://github.com/bowman-lab/enspara). The Markov state model used for this paper is publicly available at: https://osf.io/7jqyz/ (51). The main experimental data are available in *SI Appendix*, *Supplementary Tables*. Raw single-molecule photon trajectories and simulation data will be provided upon request. Plasmid of created constructs will be provided upon request. Code for analysis of single-molecule and computational data is publicly available through the sources indicated in the corresponding sections in *SI Appendix, Methods*.
