# Supplementary material for: Apolipoprotein E4 has extensive conformational heterogeneity in lipid-free and lipid-bound forms
Source: Proc Natl Acad Sci U S A. 2023 Feb 7;120(7):e2215371120. doi: 10.1073/pnas.2215371120 (PMC9963066; doi:10.1073/pnas.2215371120)
Supplement: Supplementary file 1 — Appendix 01 (PDF) [file pnas.2215371120.sapp.pdf]

PNAS

Supporting Information for

Apolipoprotein E4 has extensive conformational heterogeneity in lipid free and bound forms

Melissa D. Stuchell-Brereton[1,2], Maxwell I. Zimmerman[1,2], Justin J. Miller[1,2], Upasana L. Mallimadugula[1,2], J. Jeremias Incicco[1,2], Debjit Roy[1,2], Louis G. Smith[1,2], Jasmine Cubuk[1,2], Berevan Baban[1], Gregory T. DeKoster[1], Carl Frieden[1], Gregory R. Bowman[1,2], Andrea Soranno[1,2,*]

[1]Department of Biochemistry and Molecular Biophysics, Washington University in St Louis, 660 St Euclid Ave, 63110, Saint Louis, MO, USA
[2]Center for Science and Engineering of Living Systems, Washington University in St Louis, 1 Brookings Drive, 63130, Saint Louis, MO, USA

*corresponding author
Andrea Soranno
Email: soranno@wustl.edu

This PDF file includes:

Supporting text
Figures S1 to S24
Tables S1 to S15
SI References

Supplementary information.

Extended authorship description.

M.D.S.B. and A.S. conceived the project and designed the experiments. M.D.S.B. expressed, purified, labeled all the constructs, and performed all single-molecule FRET and FCS measurements. M.D.S.B. and A.S. analyzed all single-molecule measurements. M.Z. performed FAST simulations and developed the FRET histogram algorithm. J.J.M. performed MD simulations on Folding@home. J.J.M., U.M., L.G.S., and M.Z. analyzed the MD simulation data and M.D.S.B. and A.S. contributed to the interpretation. A.S. performed and analyzed ultra-coarse grained MonteCarlo simulations of lipid-bound ApoE. J.J.I and D.R. performed lifetime and anisotropy calibration measurements. D.R. prepared liposomes and performed and analyzed TEM experiments. J.J.I. performed and analyzed 2d-FCS experiments. J.C. performed nanosecond-FCS experiments and contributed reagents. B.B. and G.T.D. contributed reagents. C.F. supervised the experiments. M.D.S.B., G.B., J.J.M., and A.S. wrote the manuscript.

Methods.

Protein expression, purification, and labeling.

Plasmid construct design.

Apolipoprotein E protein (NCBI Reference Sequence: NP_000032.1 – for ε3 isoform) is expressed from a modified pET32a expression plasmid, which includes an N-terminal thioredoxin fusion protein, His_6 tag, and HRV 3C protease site followed by the Apolipoprotein E gene (excluding the first 18 residues, or signal peptide) as previously described[51]. The ApoE ε3 isoform gene was initially cloned into the BamHI EcoRI sites in the MCS of pET32a vector; however, the BamHI site was destroyed when the HRV 3C protease site was incorporated using site directed mutagenesis.

ATGAGCGATAAAATTATTCACCTGACTGACGACAGTTTTGACACGGATGTACTCAAAGCGGACGG
GGCGATCCTCGTCGATTTCTGGGCAGAGTGGTGCGGTCCGTGCAAAATGATCGCCCCGATTCTGG
ATGAAATCGCTGACGAATATCAGGGCAAACTGACCGTTGCAAAACTGAACATCGATCAAAACCCT
GGCACTGCGCCGAAATATGGCATCCGTGGTATCCCGACTCTGCTGCTGTTCAAAAACGGTGAAGT
GGCGGCAACCAAAGTGGGTGCACTGTCTAAAGGTCAGTTGAAAGAGTTCCTCGACGCTAACCTGG
CCGGTTCTGGTTCTGGCCATATGCACCATCATCATCATCATTCTTCTGGTCTGGTGCCACGCGGT
TCTGGTATGAAAGAAACCGCTGCTGCTAAATTCGAACGCCAGCACATGGACAGCCCAGATCTGGG

TACCGACGACGACGACCTGGAAGTGCTATTTCAAGGACCCAAGGTTGAACAGGCTGTTGAAACTG
AACCGGAACCCGAGCTGCGCCAGCAGACCGAGTGGCAGAGCGGCCAGCGCTGGGAACTGGCACTG
GGTCGCTTTTGGGATTACCTGCGCTGGGTGCAGACACTGTCTGAGCAGGTGCAGGAGGAGCTGCT
CAGCTCCCAGGTAACCCAGGAACTGAGGGCGCTGATGGACGAGACCATGAAGGAGTTGAAGGCCT
ACAAATCGGAACTGGAGGAACAACTGACCCCGGTGGCGGAGGAGACGCGGGCACGGCTGTCCAAG
GAGCTGCAGGCGGCGCAGGCCCGGCTGGGCGCGGACATGGAGGACGTGCGCGGCCGCCTGGTGCA
GTACCGCGGCGAGGTGCAGGCCATGCTCGGCCAGAGCACCGAGGAGCTGCGGGTGCGCCTCGCCT
CCCACCTGCGCAAGCTGCGTAAGCGGCTCCTCCGCGATGCCGATGACCTGCAGAAGCGCCTGGCA
GTGTACCAGGCCGGGGCCCGCGAGGGCGCCGAGCGCGGCCTCAGCGCCATCCGCGAGCGCCTGGG
GCCCCTGGTGGAACAGGGCCGCGTGCGGGCCGCCACTGTGGGCTCCCTGGCCGGCCAGCCGCTAC
AGGAGCGGGCCCAGGCCTGGGGCGAGCGGCTGCGCGCGCGGATGGAGGAGATGGGCAGCCGGACC
CGCGACCGCCTGGACGAGGTGAAGGAGCAGGTGGCGGAGGTGCGCGCCAAGCTGGAGGAGCAGGC
CCAACAGATACGCCTGCAGGCCGAGGCCTTCCAGGCCCGCCTCAAGAGCTGGTTCGAGCCCCTGG
TGGAAGACATGCAGCGCCAGTGGGCCGGGCTGGTGGAGAAGGTGCAGGCTGCCGTGGGCACCAGC
GCCGCCCCTGTGCCCAGCGACAATCAC

MSDKIIHLTDDSFDTDVLKADGAILVDFWAEWCGPCKMIAPILDEIADEYQGKLTVAKLNIDQNP
GTAPKYGIRGIPTLLLFKNGEVAATKVGALSKGQLKEFLDANLAGSGSGHMHHHHHHSSGLVPRG
SGMKETAAAKFERQHMDSPDLGTDDDDLEVLFQGPKVEQAVETEPEPELRQQTEWQSGQRWELAL
GRFWDYLRWVQTLSEQVQEELLSSQVTQELRALMDETMKELKAYKSELEEQLTPVAEETRARLSK
ELQAAQARLGADMEDVRGRLVQYRGEVQAMLGQSTEELRVRLASHLRKLRKRLLRDADDLQKRLA
VYQAGAREGAERGLSAIRERLGPLVEQGRVRAATVGSLAGQPLQERAQAWGERLRARMEEMGSRT
RDRLDEVKEQVAEVRAKLEEQAQQIRLQAEAFQARLKSWFEPLVEDMQRQWAGLVEKVQAAVGTS
AAPVPSDNH

Site-directed mutagenesis was performed on the pET32a Apolipoprotein E expression vector to create the ApoE (ε4 isoform) constructs (**Table S1**). All mutations were verified using Sanger sequencing.

Protein expression and purification.

All ApoE4 constructs were expressed recombinantly in BL21-Gold (DE3) cells (Agilent). 2L cultures were grown in LB medium containing carbenicillin (100 μg/mL) to OD_{600} ~0.8 and induced with 1 mM IPTG for 4 hours at 37 degrees C. Harvested cells were lysed with sonication at 4 degrees C in lysis buffer (50 mM Tris pH 8.0, 500 mM NaCl, 10 mg/mL lysozyme, 5 mM BME, and cOmplete™ EDTA-free Protease Inhibitor Cocktail (Roche)).

The supernatant was cleared by centrifugation (37000 rpm for 1 hour) and loaded onto a HisTrap FF column (Cytiva) in buffer A (20 mM Sodium Phosphate pH 7.5, 500 mM NaCl, 20 mM Imidazole, 5 mM BME). The Thioredoxin-His$_6$-ApoE protein fusion was eluted with Buffer B (buffer A + 500 mM Imidazole) and dialyzed into HRV 3C protease cleavage buffer (50 mM HEPES pH 7.5, 100 mM NaCl, 5 mM BME) after adding HRV 3C protease to pooled fractions. Thus, the Thioredoxin-His$_6$ fusion protein was cleaved yielding full length ApoE4 with two additional N-term residues (GlyPro). FL ApoE4 was then bound to a Heparin Sepharose FF column (Cytiva) and eluted using a step gradient from 0 to 100% buffer B (buffer A: 50 mM HEPES pH 7.5, 100 mM NaCl, 5 mM BME, buffer B: buffer A + 1 M NaCl) over 140 min. Heparin elution fractions of FL ApoE4 were pooled and dialyzed overnight into buffer A (20 mM Sodium Phosphate pH 7.3, 2 M Urea, 5 mM BME) for subsequent ion exchange chromatography. Dialyzed FL ApoE4 was then bound to a Q Sepharose FF column (Cytiva) and eluted using a gradient of 0-40% buffer B (buffer A: (20 mM Sodium Phosphate pH 7.3, 2 M Urea, 5 mM BME, buffer B: buffer A + 1 M NaCl) over 80 min, then 40-100% buffer B over 60 min. Purified FL ApoE4 variants were analyzed using SDS-PAGE and verified by electrospray ionization mass spectrometry (LC-MS). Concentrations were determined spectroscopically in 50 mM Sodium Phosphate pH 7.5, 2 M Urea, 200 mM NaCl, 5 mM BME using an extinction coefficient = 44950 M^{-1} cm^{-1}.

Choice of labeling positions.

The choice of labeling positions has been designed as a compromise between flanking regions of interest (hinge, C-terminal domain, four-helix bundle) and a series of different criteria regarding the structural properties of the protein. As there are no full-length structures available for either monomeric ApoE4 or ApoE4 oligomers, the ApoE3-like NMR structure (PDB: 2L7B) was used as a guide for identifying structural similarities with the resolved structure. In particular, we avoided altering amino acids clearly involved in structurally relevant interactions (such as aromatic residues) or previously identified functional mutations of the protein. When choosing labeling positions within folded domains, we focused on surface exposed residues to maximize accessibility of the cysteine residues during labeling. The spacing of the fluorophores was optimized to ensure use of the entire FRET dynamic range when the protein is unfolded, so that a contrast between the distance in the folded structure and in a disordered state would provide different transfer efficiencies. We also attempted to reduce the effects of

quenching due to proximity between fluorophores and aromatic residues[52,53]. With tryptophan residues identified as major quenchers for Alexa Fluor 488 and Alexa Fluor 594, fluorophores were positioned at least 10 residues away from its nearest tryptophan in the sequence.

Protein labeling.

All ApoE variants were labeled with Alexa Fluor 488 maleimide (Invitrogen Molecular Probes) under denaturing conditions in buffer A (20 mM Sodium Phosphate pH 7.3, 2 M Urea, 5 mM BME) at a dye/protein molar ratio of 0.7/1.0 at 4 degrees Celsius overnight. Single labeled ApoE protein was isolated using ion-exchange chromatography (Mono Q 5/50 GL, Cytiva – protein bound in buffer A and eluted with 0-40% buffer B (buffer A + 1 M NaCl) gradient over 80 min, then 40-100% buffer B over 20 min). UV-Vis spectroscopic analysis was used to identify fractions with 1:1 dye:protein labeling. Single labeled Alexa Fluor488 maleimide labeled N protein was then subsequently labeled with Alexa Fluor 594 maleimide at a dye/protein molar ratio of 1.2/1.0 overnight at 4 degrees Celsius. Double labeled (488:594) protein was then further purified using ion-exchange chromatography (Mono Q 5/50 GL, Cytiva –as listed above).

Liposome preparation.

Unilamellar vesicles were prepared from 1,2-dimyristoyl-sn-glycero-3-phosphocholine (DMPC) (Avanti Polar Lipids, USA) dissolved in Chloroform (25 mg/mL). The DMPC was used as received without further purification. DMPC solution containing 12.5 mg lipid was dried under a stream of dry nitrogen in glass scintillation vials. This lipid thin film was kept under vacuum overnight to remove traces of organic solvent. Liposomes were prepared by hydrating the film with 1 ml of 50 mM sodium phosphate buffer (pH 7.4) at 42 ± 1°C. The vial was shaken to form a rough liposomal suspension followed by five freeze-thaw cycles to obtain homogeneous preparation. Freezing was achieved by submersion into liquid nitrogen followed by thawing the sample in a water bath at 42 ± 1° C. The prepared vesicles were then extruded through an extruder set (Avanti Polar Lipids, USA) containing polycarbonate membranes with a pore size of 0.2 μm (Whatman plc., Cytiva Life Sciences). Finally, the extruded vesicles were flash frozen using liquid nitrogen and stored at -80°C for future use. Before the experiment, the frozen liposomes were thawed in a

water bath at 42 ± 1°C followed by five freeze/thaw cycles, according to the protocol discussed earlier. The liposomes were then re-extruded using Avanti Polar Lipids extruder set at room temperature. The extruded liposomes were kept at 4°C and used within 2 days after the preparation. In parallel to single-molecule experiments, 50 µL of the prepared liposome solutions were used for cryo-TEM imaging using JEOL JEM-1400(Plus) (JEOL USA Inc., USA) 120 kV Transmission Electron Microscope. The images were captured using an AMT XR111 CCD camera and analyzed using a Graphical User Interface developed in Mathematica (Wolfram research).

Data analysis.

Experimental setup and procedure for single-molecule fluorescence experiments. Single-molecule fluorescence measurements were performed on a Picoquant MT200 instrument (Picoquant, Germany). For single-molecule FRET measurements, a Pulsed Interleaved Excitation (PIE) scheme was obtained by synchronizing a diode laser (LDH-D-C-485, PicoQuant, Germany) and a supercontinuum laser (SuperK Extreme, NKT Photonics, Denmark), filtered by a z582/15 band pass filter (Chroma) and pulsed at 20 MHz. Emitted photons were collected through a 60x1.2 UPlanSApo Superapochromat water immersion objective (Olympus, Japan). For nanosecond FRET-FCS measurements, the same diode laser was used in continuous-wave mode to excite the donor dye. Photons emitted from the sample were collected through a 60x1.2 UPlanSApo Superapochromat water immersion objective (Olympus, Japan), passed through a dichroich mirror (ZT568rpc, Chroma, USA), a long-pass filter (HQ500LP, Chroma Technology) to suppress scattering light, and a confocal pinhole (100 µm diameter). The emitted photons were then distributed into four channels, first by a polarizing beam splitter and then by a dichroic mirror (585DCXR, Chroma) for each polarization. Donor and acceptor emission was filtered (ET525/50m or HQ642/80m, respectively, Chroma Technology) and then focused on SPAD detectors (Excelitas, USA). The arrival time of every detected photon was recorded with a HydraHarp 400 TCSPC module (PicoQuant, Germany). FRET experiments were performed by exciting the donor dye with a laser power of 100 µW (measured at the back aperture of the objective). For PIE experiments, the power used for exciting the acceptor dye was adjusted to match a total emission intensity after acceptor excitation to the one observed upon donor excitation (between 50 and 70 µW). Single-molecule FRET efficiency histograms were acquired from samples

with protein concentrations between 50 pM and 100 pM, estimated from dilutions of samples with known concentration based on absorbance measurements.

All measurements were performed in 50 mM NaPi pH 7.4, 143 mM β-mercaptoethanol (for photoprotection), 0.001% Tween 20 (for surface passivation) and GdmCl or TMAO at the reported concentrations. Tween 20 at this concentration is below the critical micelle concentration and we observe no evidence of interaction of Tween with the protein, neither interference with its ability of binding lipids. Measurements under aqueous buffer conditions are performed with PolyEthyleneGlycol (PEG)-passivated cuvettes[54], which significantly prevents adhesion of the protein to the surface. The exact concentration of denaturant is determined from measurement of the solution refractive index with an Abbe refractometer (Bausch & Lomb, USA).

Each sample was measured for at least 10 min at room temperature (295 ± 0.5 K).

FRET efficiency histograms.

Fluorescence bursts were identified by time-binning photons in bins of 1 ms and accepting bursts whose total number of photons after donor excitation was larger than at least 15 photons in each bin and contiguous bins were merged if the total number of photons was larger than at least 20 photons. The exact threshold was selected based on the background contribution identified in the photon counting histograms with 1 ms binning and a minimum common threshold across constructs has been used to minimize contributions to the width of transfer efficiency distributions due to different thresholds[30]. Transfer efficiencies for each burst were calculated according to

$$E = n_A/(n_A + n_D) \qquad \textbf{Eq. S1}$$

where n_D and n_A are the numbers of donor and acceptor photons, respectively.

Corrections for background, acceptor direct excitation, channel crosstalk, differences in detector efficiencies, and quantum yields of the dyes were applied[55]. The labeling stoichiometry ratio S was computed accordingly to:

$$S = I_D/(\gamma_{PIE} I_A + I_D) \qquad \textbf{Eq. S2}$$

where I_D and I_A represent the total intensities observed after donor and acceptor excitation and γ_{PIE} provides a correction factor to account for differences in the detection efficiency and laser intensities. Bursts with stoichiometry corresponding to 1:1 donor:acceptor labeling (in contrast to donor and acceptor only populations) were selected according to the criterion 0.3 < S < 0.7. The final histogram of transfer efficiencies was constructed from these described selected bursts. Variations in the selection criteria for the stoichiometry ratio do not impact significantly the observed mean transfer efficiency (within experimental errors).

To estimate the mean transfer efficiency and extract multiple populations from the transfer efficiency histograms, each population was approximated with either a Gaussian or a LogNormal distribution function. For fitting more than one peak, the histogram was analyzed with a sum of the abovementioned functions. When analyzing multiple overlapping populations, in order to limit the model parameters and potential overfitting, we favored the use of global fit analysis where some parameters are shared across multiple or all concentrations. A list of used parameters is provided in the following.

For **$ApoE4_{182,241}$**, data were globally fitted with the same width for each population across all concentrations of GdmCl. While this does not account for small deviations in the width due to the transfer efficiency dependence of the shot noise[30] , this option was made necessary because of the strong overlap between the two main populations across a large range of denaturant concentrations. The width was constrained between 0 and 0.09 based on the main width determined in high denaturant concentrations, where the region is completely unfolded. The upper limit value aligns with analogous values observed for other constructs.

For **$ApoE4_{223,291}$**, we performed a global fit across all GdmCl concentrations with the following constraints: the mean transfer efficiency of the population at low transfer efficiency was shared across multiple GdmCl concentrations and constrained within a range of 0.06<E<0.18; the mean transfer efficiency of the population at high transfer efficiency was shared across multiple GdmCl concentrations and constrained within a range of E>0.85; the width of the distribution associated with each population was constrained for the population at intermediate transfer efficiencies between 0.06 and 0.13, whereas the one for the population at high transfer efficiency was constrained between 0 and 0.1.

For **ApoE4$_{86, 241}$**, no global fit was necessary but the mean transfer efficiencies of the three populations were constrained such that $0.1<E_1<0.25$, $0.3<E_2<0.75$, and $0.8<E_3<0.9$ for the low, intermediate, and high transfer efficiency population, respectively. All the widths were constrained between 0 and 0.12.

For **ApoE4$_{86, 165}$**, we initially fit the data using two Gaussian distributions and a Log-Normal distribution. After the initial fit suggests an almost constant value for the folded and intermediate population, the model has been further restrained to account for these fixed positions (though corrected for the refractive index change due to the increasing denaturant. Given the small amplitude, the width of the intermediate distribution has been enforced to be 0.1 across all denaturant concentration. Similarly, the width of the unfolded state has been constrained to 0.1 up to 2 M GdmCl, where the width of the distribution can start to be determined more robustly.

For **ApoE4$_{5,86}$**, we use a single Gaussian distribution without any constraint for each denaturant concentration above 1 M GdmCl. From 1 M GdmCl, we use a combination of a Log-Normal and a Gaussian distribution. Based on an iterative fitting procedure, further constraints have been enforced, requiring that $E_1 < 0.15$ and fixing the asymmetry of the Log-Normal distribution to 1.875. This is made necessary to limit the error in the estimate of the area of the low transfer efficiency population.

Folding equilibrium.

Folding equilibrium across the identified populations is described in terms of two-state and three-state models depending on the observable of the specific construct. Here, we exemplify the two-state model for the case of an unfolded (U) and folded (F) states and the three-state model as the equilibrium between native (N), intermediate (I) and unfolded (U) states. The same models can be applied to describe the folding equilibrium between different native states, as in the case of **ApoE4$_{223, 291}$**.

For the two-state equilibrium $\mathrm{N} \rightleftharpoons \mathrm{U}$, the corresponding fraction folded and unfolded can be written in terms of the equilibrium constant K_{UN} as

$$f_U = \frac{1}{1+K_{UN}}, f_N = \frac{K_{UN}}{1+K_{UN}} \qquad \textbf{Eq. S3}$$

where $K_{UN} = \exp[-\Delta G_0^{UN}/RT + m\,c] = \exp[m/RT(c - c_{1/2})] = \exp[\Delta G_0^{UN}/c_{1/2}(c - c_{1/2})]$, where c is the denaturant concentration, $c_{1/2}$ represents the denaturant concentration at which the folding and unfolding fraction curves cross each other, e.g., when the two states have an equal abundance, ΔG_0^{UN} is the free energy difference between the N and U states extrapolated at zero denaturant concentration, R is the ideal gas constant and T is the temperature.

For the three-state equilibrium $\mathrm{N} \rightleftharpoons \mathrm{I} \rightleftharpoons \mathrm{U}$, the corresponding fraction folded, intermediate, and unfolded can be written in terms of the equilibrium constant K_{UI} and K_{IN} as

$$f_U = \frac{1}{1+K_{UI}+K_{UI}K_{IN}}, f_I = \frac{K_{UI}}{1+K_{UI}+K_{UI}K_{IN}}, f_N = \frac{K_{UI}K_{IN}}{1+K_{UI}+K_{UI}K_{IN}} \qquad \textbf{Eq. S4}$$

where $K_{UI} = \exp[\Delta G_0^{UI}/c_{1/2}^{UI}(c - c_{1/2}^{UI})]$ and $K_{IN} = \exp[\Delta G_0^{IN}/c_{1/2}^{IN}(c - c_{1/2}^{IN})]$ with ΔG_0^{UI} and ΔG_0^{IN} represent the free energy difference extrapolated to aqueous buffer conditions between the U and I and I and N states, respectively, whereas $c_{1/2}^{UI}$ and $c_{1/2}^{IN}$ are the concentration where the corresponding fraction curves cross each other. In this case, it is important to notice that the crossing point does not necessarily occur in the midpoint (50%) of the transition.

Finally, when the folding transition is not reported in terms of fractions but is estimated based on a specific signal (e.g., variation in mean transfer efficiency or width of the transfer efficiency distribution), the total signal is described as combination of specific fraction with the corresponding signal of each population, where the signals associated to each population are considered further parameters.

IRF determination. Instrument response function (IRF) for each 4 detectors and for the 2 lasers was obtained recording the TCSPC histogram for the fluorescence of Rhodamine B (RhB, 100 µM) in presence of high concentration of potassium ferricyanide (95% of a saturated solution of $K_3Fe(CN)_6$ at 23°C in water), an electron-transfer fluorescence quencher[56].

As the rate of non-radiative relaxation pathways of the fluorophore increases with addition of quencher, the overall fluorescence time decay gets faster and eventually it is fast enough in comparison with the IRF -effectively a delta function-, the observed TCSPC histogram approaches the actual IRF[57]. This is due to the fact the observed TCSPC

histogram is a time convolution of the IRF and the fluorescence relaxation kinetics of the fluorophore,

$$f(t) = \int_0^t IRF(t-\tau)I(\tau)d\tau \quad \textbf{Eq. S5}$$

and the convolution of a function with a δ-function is equal to the function itself.

Laser power was set at 0.5 μW after the main dichroic at a repetition rate of 20 MHz and decreased 100 times before measurement (final power ~ 5 nW). Data was collected with 16 ps resolution. The collected TCSPC histograms were corrected by subtracting dark counts - which appear uniformly distributed in the histogram - and normalized over the total number of counts.

For consistency, we checked if at the ferricyanide concentration used its quenching effect on the TCSPC histogram was already maximum in the conditions of our measurements. On the one hand, we found that the histograms converged and became sharper as the concentration was increased and from 0.8 M up, they were indistinguishable. On the other hand, we also found that, as the mean fluorescence intensity decreased, the computed steady-state anisotropy converged to a value of 0.37-coincident with previously reported values of fundamental anisotropy of RhB derivatives [58], as expected for emission taking place much faster than rotational relaxation from randomly oriented fluorophores with almost parallel excitation and emission transition dipolar moments[59].

We found stronger quenching and faster fluorescence decay of RhB with ferricyanide than with potassium iodide, a quencher commonly used for this purpose, both tested at the concentration of a 95% saturated solution at room temperature (~23°C). Additionally, in presence of quencher, we found no detectable differences in the normalized TSCPC histograms obtained with Alexa594 upon 589 nm excitation with 640 nm detection, but slightly slower fluorescence decay with Alexa488 in comparison to RhB, upon 485 nm excitation and 525 nm detection.

Correction factors for quantitative anisotropy determination. In order to obtain estimates of the correction coefficients L_p and L_s accounting for scrambling of polarization due to tight focusing optics we employed the technique developed by Koshioka and coworkers[60]. The methodology consists in performing a measurement of fluorescence intensity in two solutions of a fluorophore, in low and high viscosity media, recording the

signal in 2 detectors collecting light polarized parallel (p) and perpendicular (s) to that of the excitation laser. The time correlated single photon counting (TCSPC) histogram of p- and s- polarization detection for each medium is fitted as a convolution with the Instrument Response Function (IRF) and a fluorescence lifetime function containing fluorescence and rotational relaxation kinetic terms. The parameters L_p and L_s are obtained through global fitting of the TCSPC histograms from the two solutions and two polarizations.

To this end, we used maleimide-C5 Alexa488 to determine L_p and L_s in detectors for donor dye upon excitation at 485 nm, and maleimide-C5 Alexa594 dyes to determine L_p and L_s in acceptor-channel detectors upon excitation at 590 nm. The concentration of dye solutions was 5 μM in water for the low viscosity solution and in 59% v/v glycerol in water (high viscosity solution), supplemented with 240 mM beta-mercaptoethanol. Measurements were performed at 23 ± 1°C. Laser power was set at 0.5 μW after the main dichroic at a repetition rate of 20 MHz and decreased 100 times before measurement (final power ~ 5 nW). Data was collected with 16 ps resolution.

The following equations for parallel (p) and perpendicular (s) detection were simultaneously fit to the corresponding pair of experimental TCSPC histograms:

$$f_p^d(t) = \Delta\tau \sum_{j=1(\tau_j=0)}^{k(\tau_k=t)} IRF_p^{d,\lambda}(\tau_j - t)\; I_p(\tau_j)$$
$$f_s^d(t) = \Delta\tau \sum_{j=1(\tau_j=0)}^{k(\tau_k=t)} IRF_s^{d,\lambda}(\tau_j - t)\; I_s(\tau_j) \quad \textbf{Eq. S6}$$

where d denotes the detector, λ denotes the excitation laser employed, $IRF^{d,\lambda}$ is the corresponding experimentally determined instrument response function, and I_p and I_s are given by,

$$I_p(\tau) = a_p\, e^{-\frac{\tau}{\tau_F}}\left[1 + r_0(2 - 3L_p)\, e^{-\frac{\tau}{\tau_R}}\right]$$
$$I_s(\tau) = a_s\, e^{-\frac{\tau}{\tau_F}}\left[1 - r_0(1 - 3L_s)\, e^{-\frac{\tau}{\tau_R}}\right] \quad \textbf{Eq. S7}$$

where a_p and a_s are amplitude coefficients, τ_F is the fluorescence emission lifetime parameter, τ_R is a rotational diffusion characteristic time parameter, r_0 is the fundamental anisotropy parameter, and L_p and L_s are the corresponding scrambling correction coefficients for each polarization. Parameters a_p, a_s, τ_F, τ_R, L_p and L_s were floating fitting

parameters while r_0 was fix at the previously reported value for Alexa488 and Alexa594, ~ 0.38 [61]. Simultaneous fitting of **Eq. S7** to the pairs of TCSPC histograms obtained in water and in 59% v/v glycerol was performed sharing only parameters L_p and L_s, employing weighted least squares, using 1/*f*(t) as weights.
Computing the time resolved anisotropy, *r*(t),

$$r(t) = \frac{F_p(t) - G\,F_s(t)}{\left(1 - 3L_s\right)F_p(t) + \left(2 - 3L_p\right)G\,F_s(t)} \qquad \textbf{Eq. S8}$$

also requires estimation of the relative efficiencies of detection in both detectors, parallel and perpendicular, denoted by the *G* factor, i.e., the efficiency of detection compared between parallel and perpendicular polarization.

To this end, for each pair of detectors, we used 3 different approaches which show consistency with each other:
- the ratio of amplitude parameters a_p and a_s in **Eq. S7**;
- solving **Eq. S8** for *G* using independent measurements of steady-state anisotropy together with measurements in our microscope of time-average fluorescence intensity $\langle F_p \rangle$ and $\langle F_s \rangle$ and parameters L_p and L_s obtained as described above.
- fitting the value of *G* such that the time trace of $r(t)$ in **Eq. S8** obtained for free Alexa dyes in water at room temperature, decreases asymptotically to 0 and effectively reaches that value in less than 10 ns.

The values obtained of L_p, L_s and *G* for each excitation laser and each pair of detectors and employed in the analysis of the results in this work are shown in **Table S5**.

Fluorescence lifetimes analysis. We estimated the fluorescence lifetime of the donor and acceptor by globally fitting the histogram of photon arrival times with **Eq. S7**, where a_p and a_s are treated as free parameters, r_0 is fixed to 0.38 as an average value based on previous estimates for Alexa Fluor 488 and Alexa Fluor 594[61] and τ_F and τ_r are the lifetime of the fluorophore and the rotational component of the dye, respectively. To this end, we time-gate the fluorescence arrival time to separate photons derived from donor and acceptor excitation and we select for stoichiometry and transfer efficiency to restrict the analysis to each subpopulation. We found that addition of an additional fitting parameter

representing background counts is particularly useful when analyzing high transfer efficiency populations, such as for **$ApoE_{86,165}$**. Obtained values are reported in **Table S6** and **S7** for lipid-free and lipid-bound constructs. The lifetime of donor in presence or absence of acceptor is used to further compute the FRET rate. This provides an overall measurement of the characteristic timescales at play, enabling to compare whether the energy transfer occurs on timescale longer than the rotation of the fluorophores, which is commonly assumed to interpret transfer efficiency according to:

$$E = \int_{b_0}^{l_c} E(r)P(r)dr \qquad \textbf{Eq. S9}$$

Where b_0 is the shortest contact distance and l_c is the contour length of the chain assuming all amino acids are in part of a disordered polymer (i.e., equal to the number of amino acids times the C_α-C_α distance)[62].

Anisotropy analysis. Anisotropy of the donor-only, acceptor-only, and acceptor after donor excitations are computed based on **Eq. S8** from photon arrival times histograms detected on the *p*- and *s*- polarization. Time-resolved anisotropy decays are then fitted to a double exponential decay according to:

$$\left((r_0 - r_\infty)\, e^{-\frac{t}{\tau_r}} + r_\infty\right) e^{-\frac{t}{\tau_M}} \qquad \textbf{Eq. S10}$$

where r_0 is fixed to 0.38 as described above, r_∞ is the residual anisotropy at long times (as indicated by the limiting time between two excitations pulses), τ_r represents the rotational component of the dye, and τ_M reports about tumbling of the overall molecule. Paralleling the analysis of lifetime fluorescence, values for r_∞ and τ_r are reported in **Tables S8** and **S9.** τ_r can be compared to the analogous quantity obtained from lifetime fits and is always faster than the corresponding inverse of the FRET rate or donor lifetime. r_∞ are used to compute the orientation factor κ^2. Finally, we estimated also steady-state anisotropies by computing anisotropy based on the burst-determined photons in the different polarizations. The final calculation is equivalent to the one presented in **Eq. S8**, where the time-dependent factors are replaced by the number of photons in each selected burst.

***Orientation Factor κ²*.** While commonly assumed to be equal to 2/3, the orientation factor κ^2 can be estimated using residual fluorescence anisotropies, which provide boundaries on the angles sampled by fluorophores. Though this is subject to various approximations, it does provide a useful test to quantify whether the accessible space sampled by the dyes is hindered by a folded domain. Following previous treatments where tumbling of the dye is described as a wobble-in-a-cone [61,63], κ^2 can be estimate as:

$$\kappa^2 = \left(1 - \sqrt{\frac{r_A}{r_0}}\right)\left(\sqrt{\frac{r_D}{r_0}}\left(\cos\frac{\theta_+ - \theta_-}{2}\right)^2 + \frac{1}{3}\right) +$$
$$\left(1 - \sqrt{\frac{r_D}{r_0}}\right)\left(\sqrt{\frac{r_A}{r_0}}\left(\cos\frac{\theta_+ + \theta_-}{2}\right)^2 + \frac{1}{3}\right) + \frac{1}{r_0}\sqrt{r_A r_D}\left(\frac{3}{2}(\cos\theta_+ + \cos\theta_-) - \cos\beta\right)$$

Eq. S11

where $\cos\beta = \sqrt{\frac{2}{3}\frac{r_{A(D)}}{r_A r_D} + \frac{1}{3}}$. Based on previous determinations, r_0 is set to 0.38. The only two unknowns are the angles θ_+ and θ_-, whose boundaries are estimated as:

$$\cos^{-1}(\min(\cos\beta, 1)) < \theta_+ < 2\pi - \cos^{-1}(\min(\cos\beta, 1))$$ **Eq. S12a**

$$-\cos^{-1}(\min(\cos\beta, 1)) < \theta_- < \cos^{-1}(\min(\cos\beta, 1))$$ **Eq. S12b**

with $\min$ indicating the smaller among the two values. Evaluating the solution across all the possible angles in these two intervals enables reconstructing a distribution of the possible κ^2 values, which deviates from the expected distribution for freely rotating fluorophores. Given that the tumbling of fluorophores occurs on a time scale faster the fluorophore lifetime and that of the inverse of the rate of energy transfer (see **Table S6-S9**), we can compute a mean value of κ^2 from the distribution, which can be compared to the expected value of 2/3. All the measured mean values are within the range of 0.74-1.01, with a bias toward higher values (stronger hindrance) for the lipid-bound states. It is important to note that κ^2 enters in the Forster radius at the power of 1/6 and therefore such deviations from 2/3 have an impact of about 10% on the estimate value of R_0 (**Table S10-S11**).

From the distributions of κ^2, we can further evaluate the precision and accuracy associated to the estimate of mean κ^2, as well as the minimum and maximum of the distribution:

$$\text{Precision} = \int_{\kappa^2_{min}}^{\kappa^2_{max}} d\kappa^2 \left(1 - \left(\frac{3}{2}\kappa^2\right)^{-1/6}\right) P(\kappa^2) \Big/ \int_{\kappa^2_{min}}^{\kappa^2_{max}} d\kappa^2 P(\kappa^2) \qquad \textbf{Eq. S13}$$

$$\text{Accuracy} = \text{var}\left(\left(\frac{3}{2}\kappa^2\right)^{-1/6}\right) \qquad \textbf{Eq. S14}$$

$$\kappa^2_{min} = \frac{2}{3}\left(1 - \left(\sqrt{\frac{r_D}{r_0}} + \sqrt{\frac{r_A}{r_0}}\right)/2\right) \qquad \textbf{Eq. S15}$$

$$\kappa^2_{max} = \frac{2}{3}\left(1 + \sqrt{\frac{r_D}{r_0}} + \sqrt{\frac{r_A}{r_0}} + 3\sqrt{\frac{r_D}{r_0}}\sqrt{\frac{r_A}{r_0}}\right) \qquad \textbf{Eq. S16}$$

The minimum and maximum of κ^2 are analytically computed assuming there is no knowledge of $r_{\infty-A(D)}$. A computational value can be obtained by evaluating all the possible angles allowed by **Eq. S12**[61].

The terms $r_A, r_D, r_{A(D)}$ reflects the residual anisotropy of the acceptor only, donor only and acceptor after donor excitation, respectively, and can be estimated from the values of residual anisotropy associated with the dye relaxation $r_{\infty-Aonly}, r_{\infty-Donly}, r_{\infty-A(D)}$ (see previous section). As validation, we further compared the values obtained with this method with analogous estimates obtained from the corresponding steady-state anisotropies, which is often used as a first approximation for the estimate of the residual anisotropies. Importantly, steady-state anisotropies are computed as an average across population specific bursts (donor-only, acceptor-only, donor-acceptor) and therefore provide an independent estimate compared to the time resolved ones. We found a general good agreement between these two methods of estimate, suggesting that the quantification of anisotropies and κ^2 are robust.

For the conversion of transfer efficiency to distances, we then use the value of the Förster radius for Alexa Fluor 488 and Alexa Fluor 594 previously determined and reported in literature, R_0 = 5.4 nm[64], corrected by the variation in solution refractive index and κ^2.

Lifetime vs FRET. The fluorescence lifetime is related to the mean transfer efficiency[30] through:

$$\tau_{DA}/\tau_D = 1 - \langle E \rangle + \frac{\sigma^2}{1-\langle E \rangle} \qquad \textbf{Eq. S17}$$

where σ^2 is related to the variance of the sampled distribution *via*:

$$\sigma^2 = \int_0^\infty E(r)^2 P(r) dr - \langle E \rangle^2 \qquad \textbf{Eq. S18}$$

For σ^2 equal to zero, Eq. S17 reduces to the linear trend expected for a rigid distance. For fitting experimental data, we choose the $P(r)$ of a wormlike chain[62], as described by:

$$P_{WLC}(r, l_p, l_c) = \frac{4\pi (r/l_c)^2 C(l_p, l_c)}{l_c (1-(r/l_c)^2)^{9/2}} \mathrm{Exp}\left[-\frac{3\, l_c}{4 l_p (1-(r/l_c)^2)}\right] \qquad \textbf{Eq. S19a}$$

$$C(l_p, l_c) = \frac{1}{\pi^{3/2} e^{-\alpha} \alpha^{-3/2} (1 + 3/\alpha + 15/(4\alpha^2))} \qquad \textbf{Eq. S19b}$$

with $\alpha = \frac{3}{4}\frac{l_c}{l_p}$, where l_p is the persistence length. In analyzing the lifetime vs transfer efficiency data, both l_p and l_c are varied. Whereas for a given polymer the contour length is fixed, using the contour length as a fitting parameter for a region in which some parts are folding reflects the inherent change in the nature of polymer: the polymer cannot be anymore stretched to the say contour length because of the folded regions. It is an approximation used to provide an empirical quantification of the underlying distance distribution as well as to provide a qualitative measurement that accounts for the conformational changes within the protein. The limit of a completely dynamic chain is recovered for a Gaussian distribution:

$$P_G(r, \langle r^2 \rangle) = 4\pi r^2 \left(\frac{3}{2\pi \langle r^2 \rangle}\right)^{3/2} \mathrm{Exp}\left[-\frac{3}{2}\frac{r^2}{\langle r^2 \rangle}\right] \qquad \textbf{Eq. S20}$$

Nanosecond Fluorescence Correlation Spectroscopy. Autocorrelation curves of acceptor and donor channels and cross-correlation curves between acceptor and donor channels were calculated as described previously[65]. All samples have been measured at

a concentration between 100 pM and 1 nM and bursts with a transfer efficiency larger than the donor only state have been selected to eliminate the contribution of donor-only molecules to the correlation amplitude. Finally, the correlation was computed over a time window of 5 μs and characteristics timescales were extracted according to:

$$g_{ij}(\tau) = 1 + 1/N(1 - c_{AB}Exp[-(\tau - \tau_0)/\tau_{AB}])(1 + c_b Exp[-(\tau - \tau_0)/\tau_b])(1 + c_T Exp[-(\tau - \tau_0)/\tau_T]) \quad \textbf{Eq. S21}$$

where N is the mean number of molecules in the confocal volume and i and i indicate the type of signal (either from the Acceptor or Donor channels). This allows to estimate the timescale and amplitudes related to photon antibunching τ_{AB} and c_{AB}, chain dynamics τ_b and c_b, and triplet blinking of the dyes τ_T and c_T.

Burst Variance analysis. To further test for dynamics within the C-terminal region, where we observe a broadening of the distribution of distances for **ApoE4$_{223,291}$**, we performed burst variance analysis[48]. In short, we compute for define a *proximity ratio* E* by substituting in Eq. 1 the uncorrected counts n_A^u and n_D^u from the acceptor and donor channel:

$$E^* = \frac{n_A^u}{n_A^u + n_A^u} \quad \textbf{Eq. S22a}$$

and a standard deviation by using:

$$\sigma E^* = \sqrt{\frac{E^*(1-E^*)}{n}} \quad \textbf{Eq. S22b}$$

where $n = n_A^u + n_A^u$, i.e., the total number of selected photons.

These two equations provide the theoretical expectations for the proximity ratio and its standard deviation based on a given photon-window of n consecutives photons.

We then compute the experimental expectations for each burst (E_i^*, s_i) by defining:

$$E_i^* = \frac{1}{M_i}\sum_{j=1}^{M_i} E_{ij}^* \quad \textbf{Eq. S23a}$$

$$s_i = \sqrt{\frac{1}{M_i}(E_{ij}^* - E_i^*)^2} \quad \textbf{Eq. S23b}$$

where E_{ij}^* is the proximity ratio of the photon-window j in the burst i, M_i is the number of photon-windows of n photons in the burst i.

We use a photon-window of 5 photon and report the estimated values from each burst as well as the average based on binning all proximity ratios in bins of width 0.02. See **Figure S21.**

Small deviations from the theoretical line suggests some degree of dynamics on the microsecond timescale for the intermediate transfer efficiency population of **ApoE4$_{223,291}$**. To understand whether these dynamics propagates also to the hinge, we compared the results with the ones for construct **ApoE4$_{182,241}$** finding a similar change in the main population.

MD simulations of lipid-free ApoE4. The NMR structure of ApoE3 (2L7B) was used as a starting model for all our simulations. Using pymol's mutagenesis wizard, we restored the native, mature, sequence of ApoE3 and made subsequent mutations from this model to acquire structures for ApoE2 (R158C), ApoE4 (C112R), and ApoE3 Christchurch (R136S). Each model was solvated in a dodecahedron box with 2.1 nm between the protein and the edge of the box. Systems were subsequently solvated, Na^+ and Cl^- ions were added to a final concentration of 0.1 M with no net system charge, and the system was energy minimized with a steepest descents algorithm until the maximum force fell below 100 kJ mol^{-1} nm^{-1}, using a step size of 0.01 nm and a cut-off distance of 1.2 nm for the Coulomb interactions, van der Waals interactions, and neighbors list. The AMBER03 force field[66] and explicit TIP3P solvent[67] were used for all simulations. Systems were subsequently equilibrated for 1.0 ns, where all bonds were constrained with the LINCS algorithm[68] and virtual sites to allow for a 4.0 fs timestep. Cut-offs of 1.1 nm were used for the neighbor list with 0.9 for Coulomb and van der Waals interactions. We deployed the particle mesh Ewald method for treatment of long-range interactions with a Fourier spacing of 0.12 nm. The Verlet cut-off scheme was used for the neighbor list. The stochastic velocity rescaling (v-rescale) thermostat[69] was used to hold the temperature at 300K. All simulations were prepared using Gromacs[70] 2020.

We ran our FAST adaptive sampling algorithm[50] on each of these models to explore distances between the following residue pairs: R92-W264, P183-K242, and R224-A292. The FAST algorithm balances directed exploration and unbiased simulations to efficiently explore conformational space of proteins. The algorithm proceeds as follows: 1) run initial simulations, 2) build a Markov State Model (MSM) for the aggregate simulation time, 3) rank each observed state in the MSM based on the exploration parameter, 4) restart

simulations from the top ranked states, 5) repeat steps 2-4 until the specified number of FAST rounds is complete. For each model, we ran our FAST-sampling algorithm at 300 K for 12 rounds, followed by another 8 rounds of FAST-string (20 total) with 10 simulations per round[71]. Each simulation was 40 ns in length, for a total simulation time of 8 μs for each variant. For ApoE4 we performed 2 independent rounds of the above FAST sampling. All FAST simulations were performed using Gromacs.

As ApoE is highly flexible, we wanted to ensure we had robust sampling to adequately calculate the probability of each state. Using enspara's clustering app[72], we clustered the FAST simulations from all mutants into a shared state space model coarse-grained to a final RMSD of 3.5 Å. This clustering resulted in a total of 18,182 discrete states. As each cluster center could come from any ApoE variant, we utilized MODELLER[73] to mutate all sequences back to ApoE4. Subsequently, we solvated each state in a dodecahedron box whose edges extend 1.0 nm beyond ApoE. The remainder of the system preparation followed that of the FAST system preparation. We next launched five independent simulations from each structure on the distributed computing platform, Folding@home. Each trajectory ran for a maximum of 100 ns, though the average trajectory length was 37 ns, for an aggregate simulation time of 3.43 ms. Simulations were performed using OpenMM[74], using the Langevin Integrator, a step size of 2 fs, and a temperature of 300K. During our simulations, some trajectories resulted in protein unfolding and caused ApoE to interact with its periodic image. These trajectories, along with trajectories that had improper periodic boundary condition removal, were excluded from our analysis resulting in a total of 3.37 ms of aggregate simulation time analyzed.

As the C-terminal domain of ApoE is highly flexible, we were concerned that clustering on all-atom features such as RMSD would yield a poorly connected state-space. Accordingly, we clustered ApoE on the distances between 15 different residue pairs distributed throughout ApoE. We selected these pairs to both ensure that we had an overall understanding of various aspects of ApoE movement, including extension of the C-terminal domain, movement of the N-terminal domain, motions between each domain, and the five distances measured in our single-molecule FRET experiments. The total list of residue pairs is in **Table S15**. Following coarse-graining, we generated a Markov State Model (MSM) using enspara's MSMBuilder[72] with a lag time of 10 ns (**Figure S13**)

Post-hoc calculation of FRET histograms. FRET histograms were calculated through a kinetic Monte Carlo simulation using the constructed Markov State Model (MSM). We designed this simulation to replicate the experimental process of single molecule FRET. First, we ran nearly a microsecond of MD simulations for both cysteine-Alexa 488 and cysteine-Alexa 594 using the same parameters as our all-atom ApoE simulations. Next, we aligned the cysteine backbones for all frames and the atomic coordinates of the center of mass for the dye's photon acceptor/donor region were taken as coordinates to form a dye point cloud. Accordingly, each point in the point cloud represents a position that the dye head was located in the simulation. Then, we align the center of the dye point clouds onto each structure in the MSM at both the labeling sites for a given FRET construct, removing any dye positions that touch or overlap with the protein. Finally, we compute the distribution of distances between each potential dye position.

Next, we simulate smFRET by recoloring an experimental photon burst. To do this, we select the photon arrival times from an experimental FRET trace and simulate a synthetic trajectory from our MSM of length αt, where α is a time rescaling factor and t is the total length of time of the experimental FRET burst. The states visited during the synthetic trajectory are governed by the transition probabilities of our MSM model. At each photon arrival time in the experimental burst, we select the corresponding state in our synthetic trajectory and randomly choose a position of the FRET dyes based on the previously calculated distribution probability for that state. To determine whether the observed photon is the result of a FRET event, or a donor emission, we convert the inter-dye distance into a FRET transfer probability based on the following equation:

$$E(r) = R_0^6/(R_0^6 + r^6) \qquad \textbf{Eq. S24}$$

In each case, the transfer probability is compared to a random number between 0 and 1 to determine if the photon was emitted as a donor or acceptor photon. This process is repeated for each photon event in the trajectory, and the resulting acceptor photons are summed and divided by the total number of photons to yield the FRET efficiency for the simulated burst. This process is repeated for all bursts in our experimental photon trace and the resulting FRET efficiencies are summed and plotted as the displayed FRET histograms. As smFRET is highly sensitive to changes in timescale and MD simulations can be faster than experimental time, we fit the time rescaling factor, α, using the

experimental FRET distributions (see **Figure S24).** The code for this kinetic Monte Carlo simulation has been developed as a command line app and is distributed via the enspara github (https://github.com/bowman-lab/enspara).

Interpretation of discrepancies between experimental and simulated single-molecule FRET data. As described in the previous section, calculation of single-molecule FRET data based upon MD simulations requires important assumptions. It is important to note that large discrepancies in transfer efficiencies do not directly transform in large discrepancies on distances, especially with transfer efficiency near 0.5. For example, assuming a fixed distance and a Förster radius of 5.4 nm, the difference between a mean value of transfer efficiency of 0.4 and 0.62 is equal to a change in distance from 5 to 5.7 nm. The following list represents current challenges in experiment and simulations that are commonly recognized in the field and that may lead to minor discrepancies such as the ones observed in our experiments. Indeed, it is often common to rescale simulations for matching experimental results or to use experimental results to constrain simulated conformational ensembles. While these are both valid alternatives, they introduce a bias due to the experimentally determined quantities. Few cases report an unbiased comparison of simulated transfer efficiencies and experiments and provide examples where mean transfer efficiencies can be as far apart even of 0.15 or 0.2, such as in ref. [75].
The role of κ^2. Not only does the mean transfer efficiency per burst depend on the average of multiple photons detected from different conformations of the protein, but the value of the Förster radius itself can be influenced by various factors. In **Fig. 3**, we compared simulated and experimental transfer efficiencies assuming the same Förster radius for all the constructs (5.4 nm). However, we know that the dipole orientation term κ^2 can vary across each construct and even across each subpopulation. We report the effect of variations of κ^2 across the experimentally determined boundaries (see corresponding section) on the simulated histogram in **Fig. S8.** Note that the distribution of values of κ^2 represents a distribution of possible solutions to **Eq. S11-S16**, but do not indicate a measure of correctness of a given solution. In other words, the experimental constrains can be skewed toward one of the extremes of the distribution. Variations in κ^2 represent a change in the accessible volume and relative orientation of the dyes. Overall, alterations in κ^2 are sufficient to explain all the mean transfer efficiencies discrepancies observed between experiments and simulations (**Fig. S8).** Small alterations in the Förster radius due to κ^2 can explain the larger overlap of populations observed in the experiments for

ApoE$_{5,86}$ compared to simulations. The more significant deviations occur for constructs **ApoE$_{182,241}$** and **ApoE$_{86,241}$** and suggest that the shared position 241 may be the one involved in a significant alteration of the angles explored by the dyes.

<u>Donor quantum yield and dye quenching.</u> Another element entering in the Forster radius is the quantum yield of the dye. Though we observe no significant variations in the lifetime, we cannot exclude modulations of the quantum yield, which would directly impact the estimate of the Forster radius. In addition, in ns-FCS experiments (see **Fig. S21**), we observe minor correlated amplitude in the donor-acceptor cross correlation for **ApoE4$_{182,241}$** and **ApoE4$_{223,291}$** that hint to a possible contribution of quenching due to dye-quencher or dye-dye interactions. This contribution would result in a lower experimental transfer efficiency than the one expected in absence of quenching, though the contribution is probably minor.

<u>The role of cysteine mutations.</u> Introduction of dyes via cysteine-maleimide chemistry required the mutation of amino acids in the wild-type sequence to cysteines. Some of these mutations may alter some of the torsional angle properties of the chain. Indeed, while we chose residues that are part of unstructured regions, substitution of a glycine to cysteine (e.g., G165C and G182C) may result in an alteration of the dihedral angles and impact corresponding conformations. This can contribute to the larger discrepancy observed between experimental and simulated **ApoE$_{182,241}$** data (explaining why this construct requires a larger κ^2 than **ApoE$_{86,241}$**), while it has no impact on **ApoE$_{86,165}$** because position 165 is at the bottom of the four-helix bundle.

<u>The role of the physico-chemical properties of the fluorophores.</u> Addition of explicit dyes in the simulation is not a feasible strategy, largely because it requires running 5 independent simulations (of several millisecond each) to test the effect of dyes on each position. We opted for a more effective strategy to insert dyes post-hoc. However, this simplification neglect to account for any specific chemistry and physical properties of the dyes (such as electrostatics).

<u>Simulations force-field.</u> While force-fields are constantly improved, there is still a lack of convergence on force-field that correctly reproduce disordered conformational ensembles. Small variations in the interaction energies between residues may influence the local organization of the protein and introduce discrepancies with the measured data, which can impact both conformations and the relative abundance of a specific subpopulation. Indeed, previous test of various force fields and water models on disordered proteins have reported very different results compared to experimentally determined configuration by

FRET (or other methods)[76]. Though ApoE is not completely unfolded, the choice of the specific force field may play a substantial role. Finally, while the simulation explored a large portion of the energy landscape, it may not have explored all the very low populated states, such as the intermediate and unfolded state reported for **ApoE$_{86,165}$**.

Coarse-grained model of lipid-bound states. While an atomically-detailed description of the protein requires all-atom simulations that are beyond the scope of this work, to provide a possible interpretation of the lipid-bound states observed with FRET measurements, we performed high coarse-grained MonteCarlo simulations of the protein that satisfy the FRET constraints. The scope of these simulations is purely illustrative and does not account for specific interactions with lipids or within the protein; instead, we aim only to provide a possible visual interpretation of the FRET data. To this end, we simplify the structure of the protein to a set of random coils (corresponding to the disordered regions) and rigid sticks (corresponding to the alpha helices), under the assumption that these elements are maintained in the lipid-bound state. Random coils are simulated by identifying the length of the segment from a distribution that follows the statistics of:

$$P(r,N) = (1/0.18\,N^{-3/2})4\pi r^2 \mathrm{Exp}[-3/2\,r^2/(0.38*0.4*N)] \qquad \textbf{Eq. S23}$$

based on previous estimates for a disordered region[77]. Since disordered segments are often significantly shorts, their main effect is to contribute to protein flexibility and, therefore, we neglect effects of local compaction or expansion of disordered regions due to differences in sequence composition. To enforce bound geometries, we reject all configurations that are outside a spherical corona of radius 8.67 nm and thickness 0.1 nm. For disordered regions this entails checking whether the beginning or end of the segment are within the spherical corona. For the rigid regions, this is performed for the end, middle point, and beginning of the rigid region. We further allow for overlapping configurations of the protein to facilitate the search of configurations in which helices could come in contact. We start the simulation by simulating the protein region between 86 and 241, since it displays heterogeneity in the experimental data and entails three experimental constraints (as given by **ApoE4$_{86,165}$**, **ApoE4$_{182,241}$**, and **ApoE4$_{86,241}$**). Out of 500,000 productive results of the simulation, only 1 configuration is found to satisfy the experimental results for the expanded state of **ApoE4$_{86,165}$** and only 2 configurations satisfy the other state. We

then append to the resulting states an additional 50,000 configurations of the residues between 1 and 86 and 50’000 configurations of the residues between 241 and 299. By using the additional experimental constraints, we obtain a total of 39249 configurations of the protein, where 4361 elements differ for the N-terminal tail and 9 for the C-terminal domain. The lowest number of configurations identified in the C-terminal domain stems from the larger number of constraints imposed on position 241. Two examples of configurations are reported in **Fig. 5** to provide an interpretative model of the lipid-bound state of the protein.

ApoE oligomerization.

Previous work on oligomerization kinetics of ApoE using ensemble FRET has provided estimates for the dimerization and tetramerization rates of ApoE4 and corresponding equilibrium dissociation constants for dimer and tetramer[18]. The dissociation constant between the monomer and dimer state is determined to be $K_D^{mon-dim}$ ~ 80 nM, whereas the dissociation constant between dimers and tetramers is $K_D^{dim-tet}$ ~ 20 nM. As a result, previous analysis (see **Fig. S1a** and compare with **Fig. 8d** in [18]) suggests that:

- at 10 nM of total protein concentration, ~20% of ApoE4 molecules form dimers;
- at 100 nM of total protein concentration, ~30% of ApoE4 molecules form dimers and an additional ~30% is assembled in tetramers;
- at 1 μM of total protein concentration, ~80% and ~15% of ApoE4 molecules are in the tetramer and dimer forms, respectively;
- at 10 μM of total protein concentration, ~95% of ApoE4 molecules are part of tetramer species.

To verify whether our measurements are performed in a regime under which the protein is monomeric, we evaluate changes of the labeling stoichiometry ratio as function of GdmCl. Increasing denaturant concentration shifts the equilibrium stability of dimer and tetramers and at high GdmCl concentration the protein is only monomeric. We quantify changes in labeling stoichiometry across five different regions of the stoichiometry ratio: below 0.125 (acceptor-only), between 0.125 and 0.375 (3A:1D), between 0.375 and 0.625 (1D:1A), between 0.625 and 0.875 (3D:1A), and above 0.875 (acceptor-only). In all measurements (e.g., **Fig. S1b**) across all denaturant concentrations we observe only three populations centered at 0.5 stoichiometry ratio (1D:1A) and at donor- and acceptor-only stoichiometry ratio.

Note that for the labeling procedure, we do expect to have saturated labeling of the cysteines for donor and acceptor only populations (2 donor dyes for donor-only, 2 acceptor dyes for acceptor-only) and therefore we expect 4 fluorophores in a labeled dimer. Based on the labeling procedure, the fraction of unlabeled molecules is negligible.

Fraction of molecules at different stoichiometries (3D:1A or 3A:1D) represents symmetric tails of the central distribution at 0.5 stoichiometry and the small deviations reported in **Fig. S1c** should be interpreted as modulations in the brightness or quantum yield of the labeled species. If oligomers where present in solution, we would expect to observe a decrease of intermediate stoichiometries in favor of other stoichiometries, in particular donor- and acceptor-only. If any percentage of oligomers is present is within the ~5% variation reported in the fluctuations in **Fig. S1c**.

Supplementary Figures.

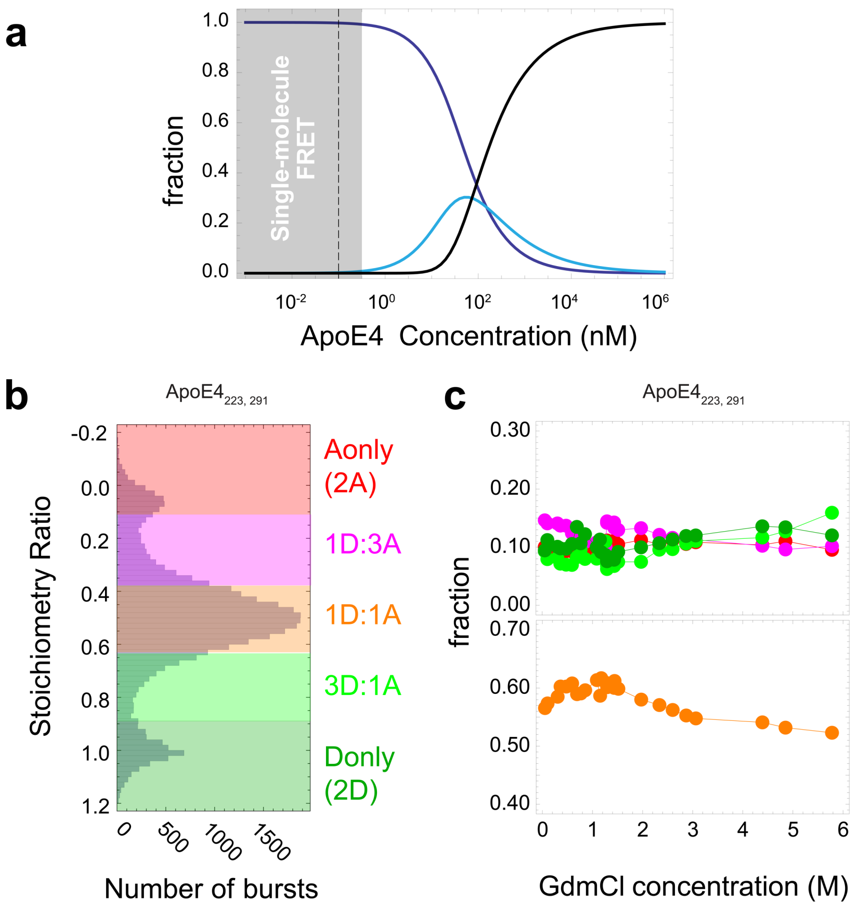

Figure S1: **Oligomerization of Apolipoprotein E. a.** Estimates of the concentration-dependent fractions of monomer (blue), dimer (cyan), and tetramer (black) for ApoE4 based on ref. [18]. **b.** Example of labeling stoichiometry ratio for $ApoE4_{223,291}$. **c.** fractions associated with the different labeling stoichiometry ratios across multiple GdmCl concentration. Across all concentrations of denaturant, the fraction of population with 1D:1A is mainly constant supporting the absence of oligomer formation. In the case of oligomer formation, dimers and tetramers of acceptor-only (Aonly) or donor-only (Donly) population would contribute to an increase in the 1D:1A, which is not observed (overall deviations are within a few percent).

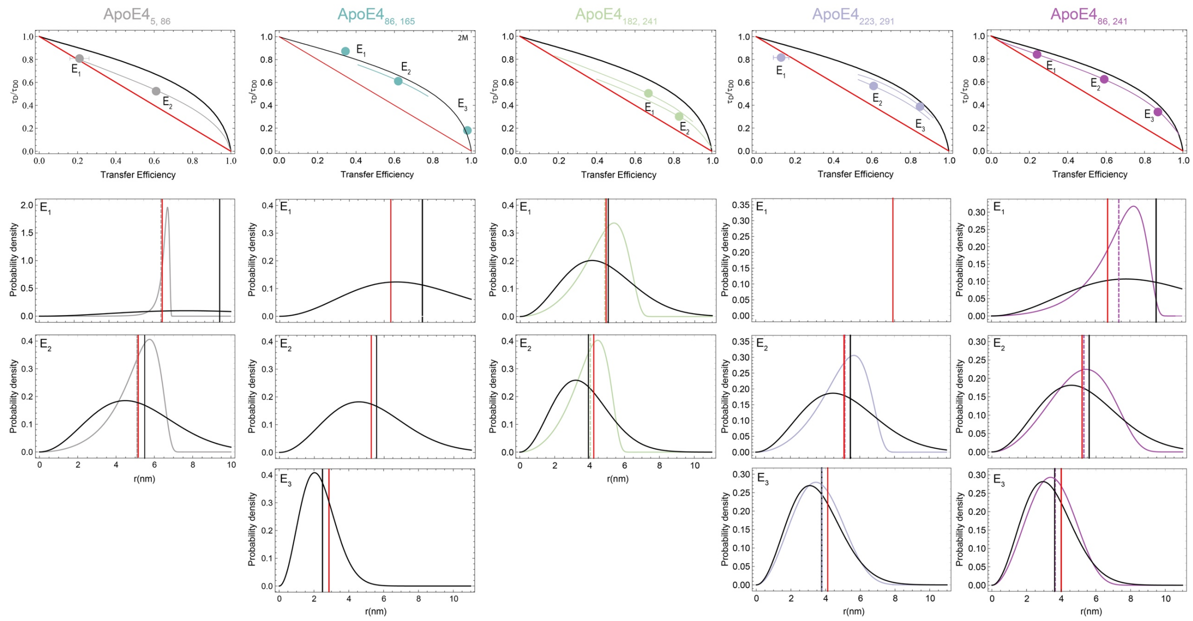

Figure S2: **Lifetime *vs* transfer efficiency plot and corresponding distance distributions for each of the five ApoE4 constructs measured in aqueous buffer conditions (**50 mM NaPi pH 7.4). The solid red line indicates the expected result for a rigid distance. The solid black line reports the expected trend for a Gaussian chain distribution whose mean square end-to-end distance satisfies the measured mean transfer efficiency. The colored line represents the best fit to a wormlike chain, where both persistence length and contour length are fitted to satisfy the constraints imposed by the measured mean transfer efficiency and mean lifetime.

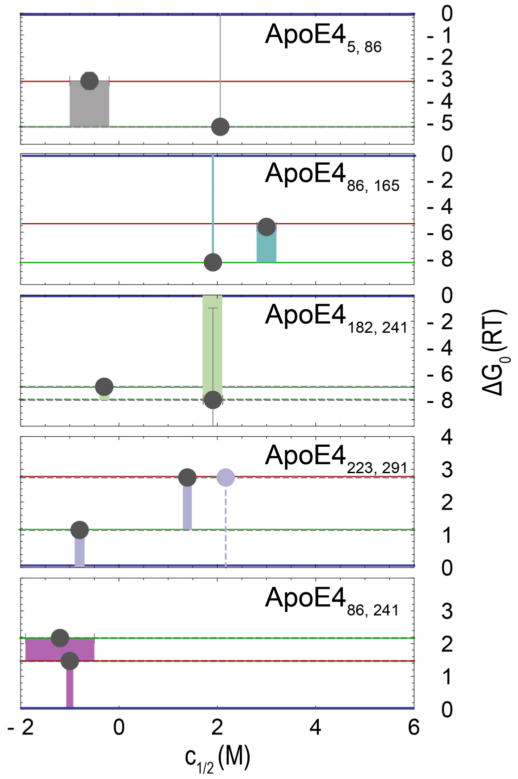

Figure S3. Folding free energy and midpoint of the transition determined from the folding equilibrium analysis. Plot of fitting parameters in **Table S2** obtained from the folding equilibrium analysis of **Fig. 2** with **Eq. S4**. Blue, red, and green line represent the corresponding states from which the ΔG_0 are computed. Gray (**ApoE4$_{5,86}$**), teal (**ApoE4$_{86,165}$**), green (**ApoE4$_{182,241}$**), purple (**ApoE4$_{223,291}$**) and violet bars (**ApoE4$_{86,241}$**) connect the states associated with the computed ΔG_0. Width of the bars represents the associated error on the corresponding $c_{1/2}$, whereas vertical error bars quantify the error (from the fit) associated with ΔG_0. In some cases, error bars may be occluded by the data points. Dashed vertical line in **ApoE4$_{223,291}$** identifies the transition in **Fig. S5** when analyzed with a two-state model.

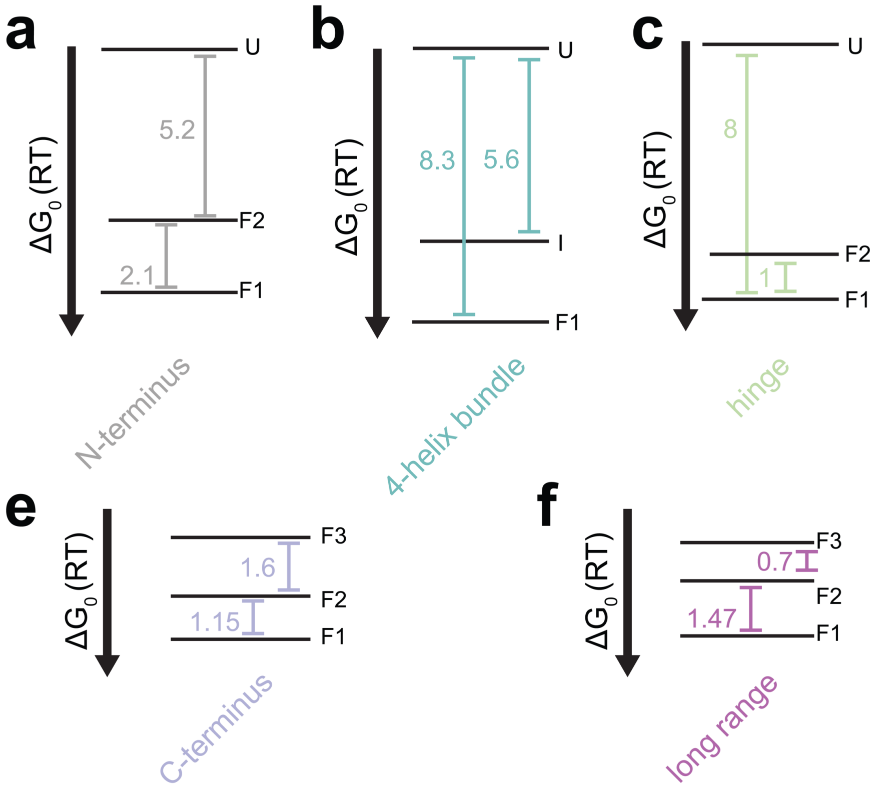

Figure S4. **Folding stability of individual constructs.** Free energy diagram of identified states in the 4-helix bundle, hinge, N- and- C- terminus, and from long range measurements. Source for the composed picture reported in **Fig. 3c.**

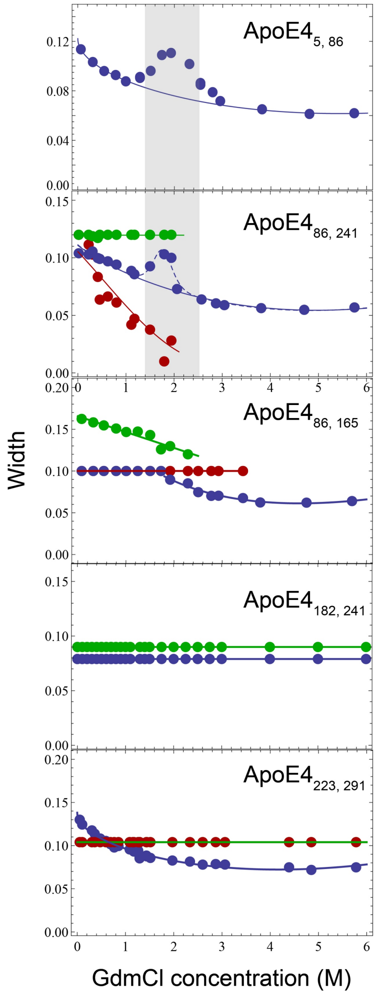

Figure S5: Widths of transfer efficiency distributions attributed to independent populations in each construct. Solid lines are polynomial fits to help guiding the eyes. The widths of **ApoE4$_{5,86}$** and **ApoE4$_{86,241}$** exhibit an increase between 1.25 and 2.5 M GdmCl suggesting coexistence of two independent configurations under the same peak, which we interpret as a conformational shift occurring during the folding of the four-helix bundle. In **ApoE4$_{182,241}$** the widths of the two populations are best described using a shared parameter for each population in the global fit of the denaturant titration. In **ApoE4$_{223,291}$**, the width of the main population increases when decreasing GdmCl concentration from 1 M to 0 M, which is compatible with the range where previous experiments identified formation of structure in the C-terminal domain. Due to the small amplitudes of the low and high transfer efficiencies populations in **ApoE4$_{223,291}$**, the corresponding widths were fixed across all concentrations. Similarly, in **ApoE4$_{86,165}$**, due to the small amplitude of the unfolded and intermediate state, below 3 M GdmCl the widths of the corresponding transfer efficiency distribution were fixed. Errors associated with repeated measurements are reported in **Table S1**.

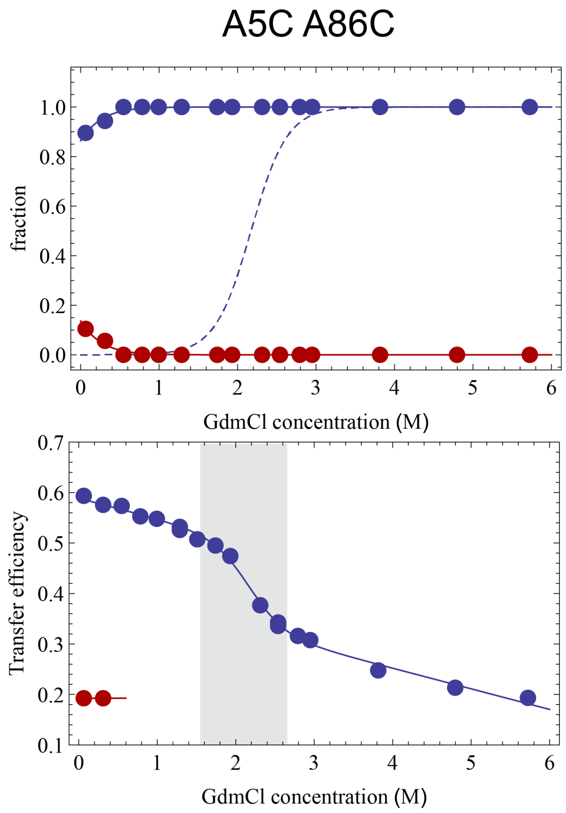

Figure S6. Analysis of the **ApoE4$_{5,86}$** construct using one single population in the range from 0.5 and 6 M GdmCl. When fitting the transfer efficiency with one single population, we observe a jump in transfer efficiencies (lower panel) and a broadening of the width (see **Fig. S5**) in the range between 1.5 and 2.5 M GdmCl. The range of the transition corresponds to the folding transition of the four-helix bundle. Corresponding values of the fit are reported in **Table S2**.

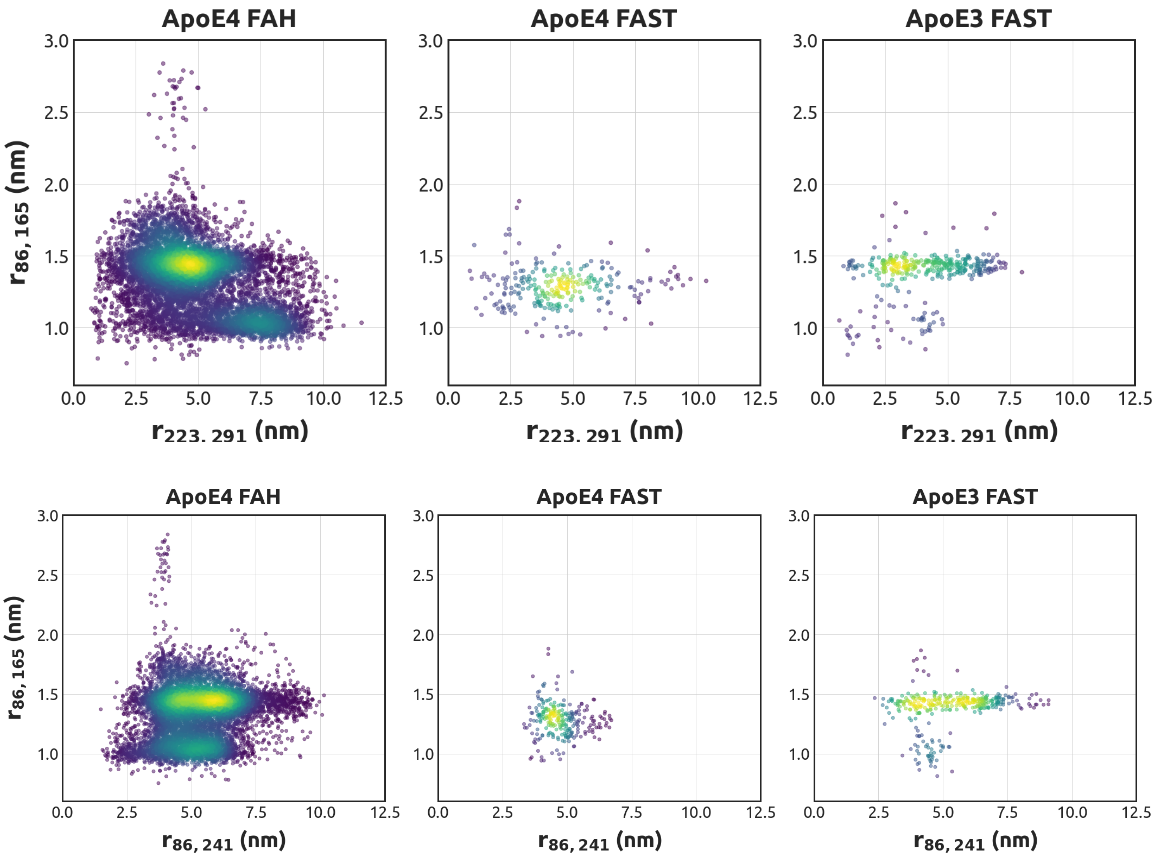

Figure S7: Comparison of FAST simulations of ApoE4 and ApoE3. Distance distributions obtained from MSMs constructed by clustering the simulations on distances between 15 residue pairs as described in Methods for A) 3.43 ms of aggregate simulations of ApoE4 performed on Folding@Home with a lag time of 10 ns. B) 8 us of FAST simulations of ApoE4 performed as described in Methods with a lag time of 10 ns and C) 8 us of FAST simulations of ApoE3 performed in the same method as ApoE4. **Upper panels: $ApoE4_{223,291}$ Lower panels: $ApoE4_{86,241}$** FAST simulations capture only a small portion of the complex energy landscape of the protein observed in Folding@Home simulations.

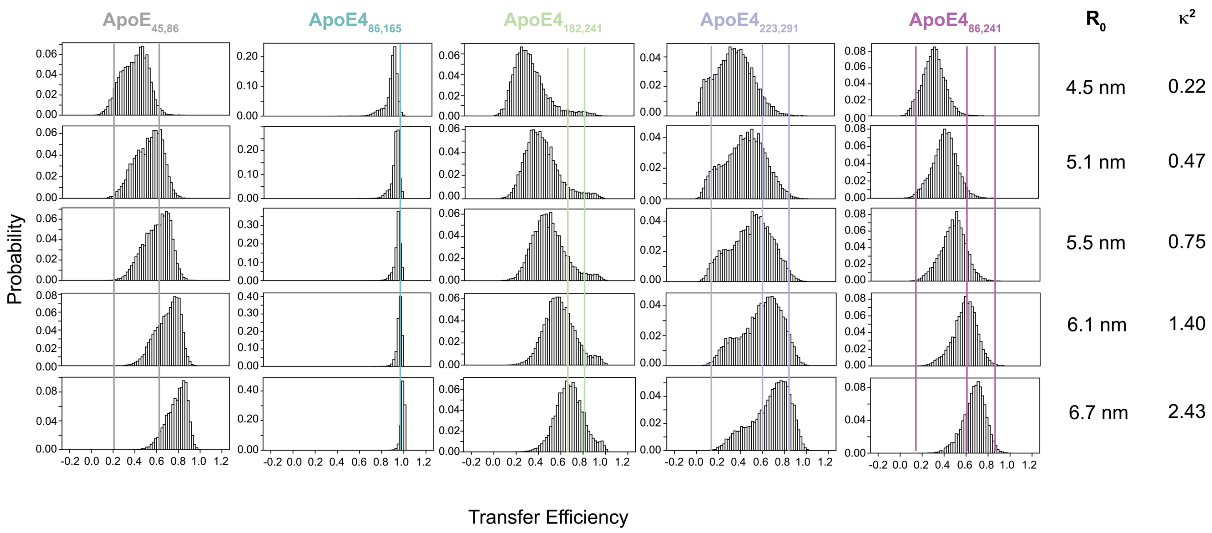

Figure S8. Simulated transfer efficiency histograms as function of κ^2. Simulated transfer efficiency histograms with κ^2 values across the range established from anisotropy measurements. Colored vertical lines identify mean values of transfer efficiency for each population in single-molecule FRET experiments. Note that for each subpopulation measured across the same construct, a different κ^2 can be adopted.

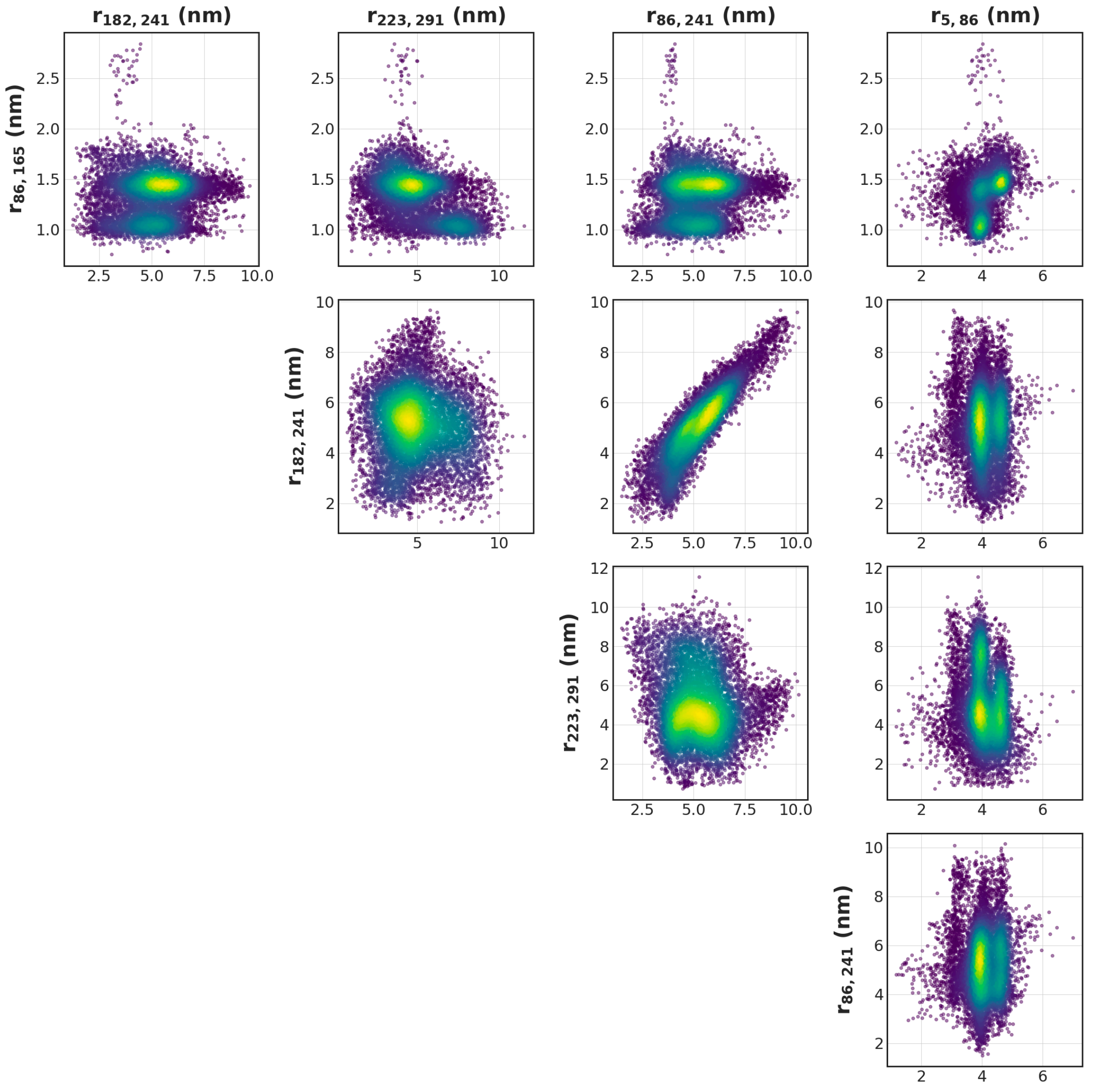

Figure S9. Correlations across different pair distances in lipid-free ApoE4. Distance pair correlations from MD simulations contrasting all labeled distance pairs.

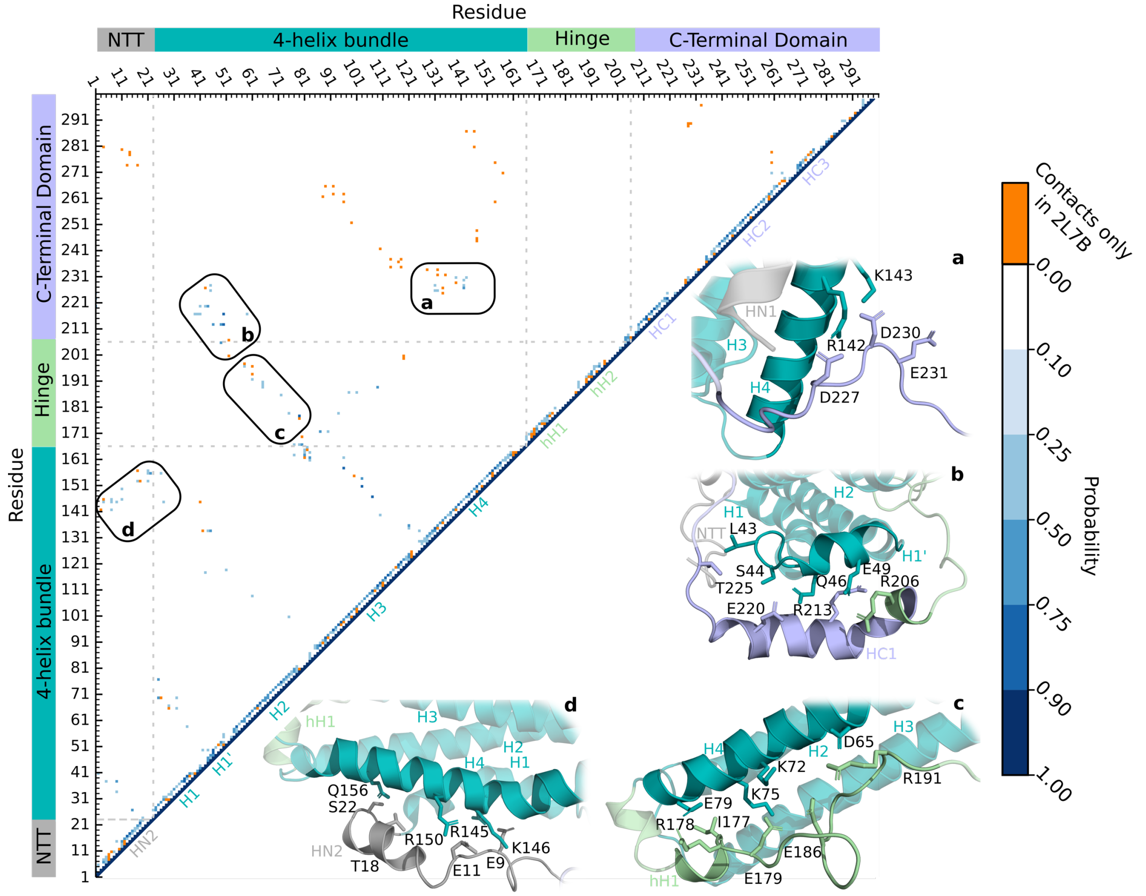

Figure S10. Contacts in lipid-free ApoE4. Probability contact map from all structures from MD simulations contrasted against contacts found in the ApoE3-like NMR structure (PDB:2L7B). Contacts defined as any heavy atom between two residues being within 3 Å. Contacts that are present only in the NMR structure are reported in orange. Black boxes identify contacts that are due to domain-domain interactions.

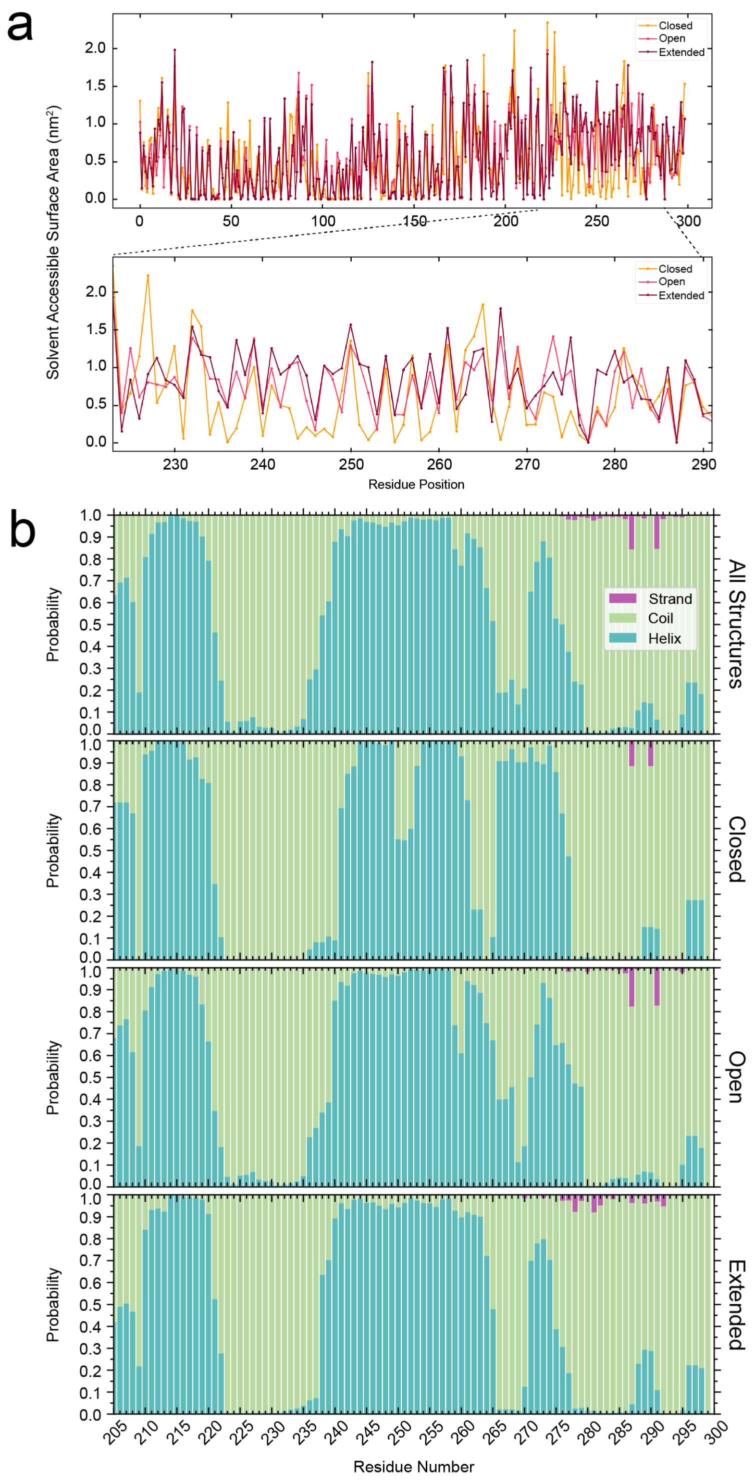

Figure S11. Solvent Accessible Surface Area and structural content of the C-terminal region. a. Solvent Accessible Surface Area. Specific residues between 230 and 280 are more protected in the *extended* conformation. **b.** Secondary structure content in the C-terminal domain across the three *closed*, *open*, and *extended* subpopulations supports the presence of a structured helix with some degree of flexibility because of disordered regions.

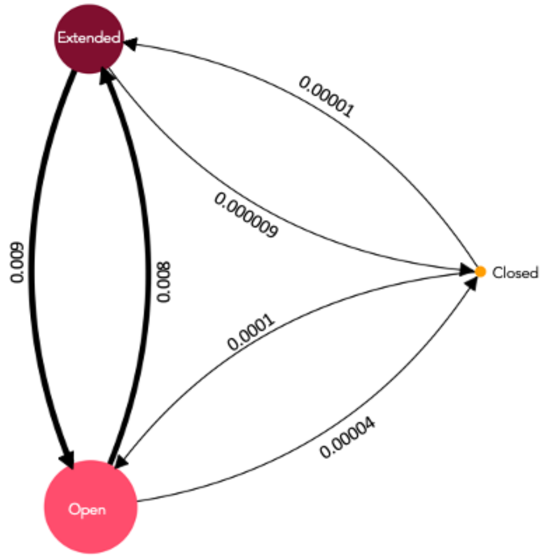

Figure S12. Graphical representation of the MSM states belonging to the open, closed and extended configurations identified in Fig 4. The nodes representing each state are sized proportional to the sum of the equilibrium probabilities of all the MSM states belonging to that node. The arrows indicate aggregate transition probabilities between the nodes.

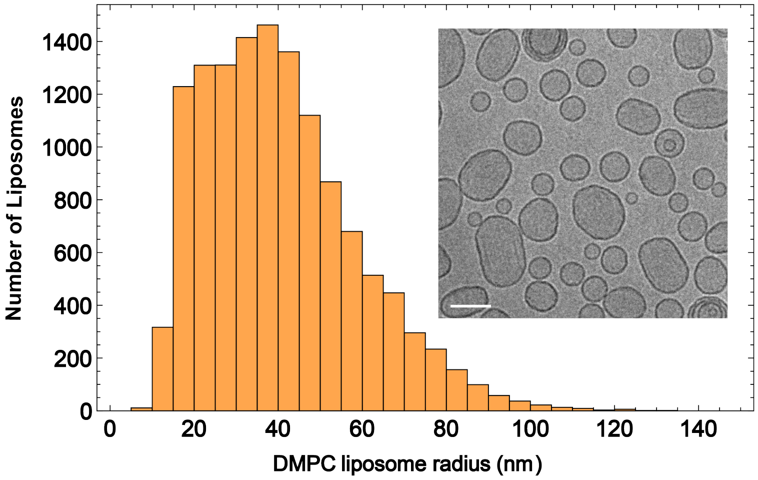

Figure S13: Size distribution of extruded liposomes determined from cryo-TEM images (inset). The bar on the inset figure represents 100 nm.

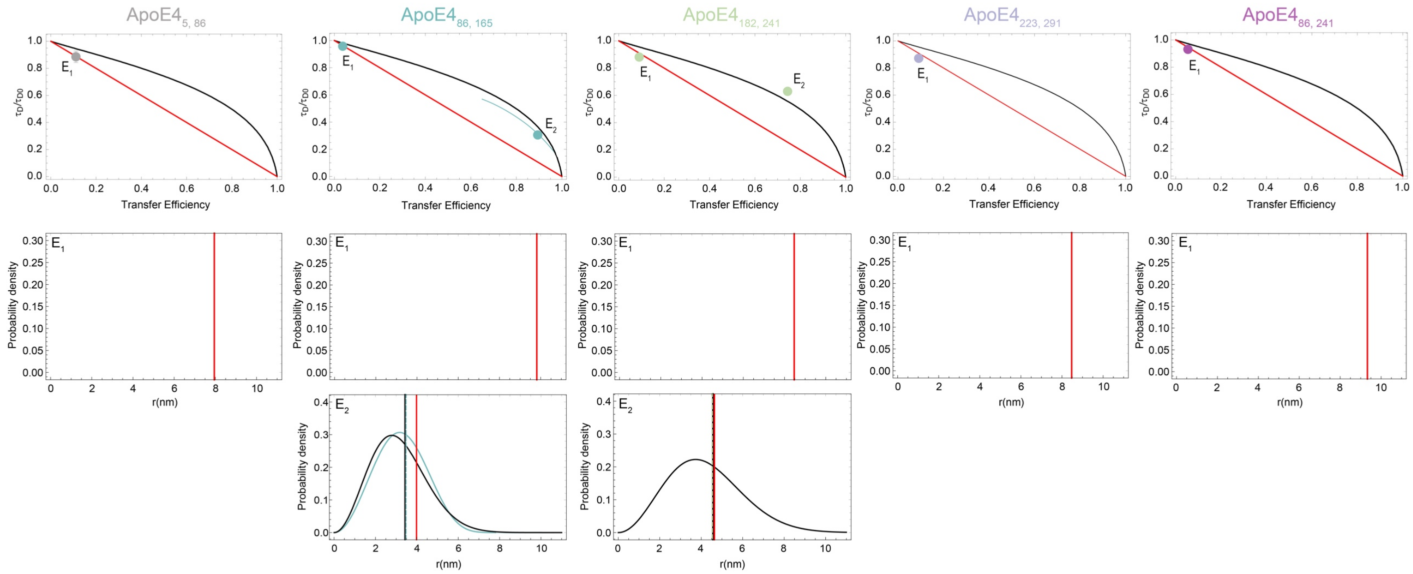

Figure S14: **Lifetime *vs* transfer efficiency plot and corresponding distance distributions for each of the five ApoE4 constructs measured in the lipid-bound state** (100 µg/mL extruded liposomes, 50 mM NaPi pH 7.4). The solid red line indicates the expected result for a rigid distance. The solid black line reports the expected trend for a Gaussian distribution whose mean square end-to-end distance satisfies the measured mean transfer efficiency. The colored line represents the best fit to a wormlike chain, where both persistence length and contour length are fitted to satisfy the constraints imposed by the measured mean transfer efficiency and mean lifetime.

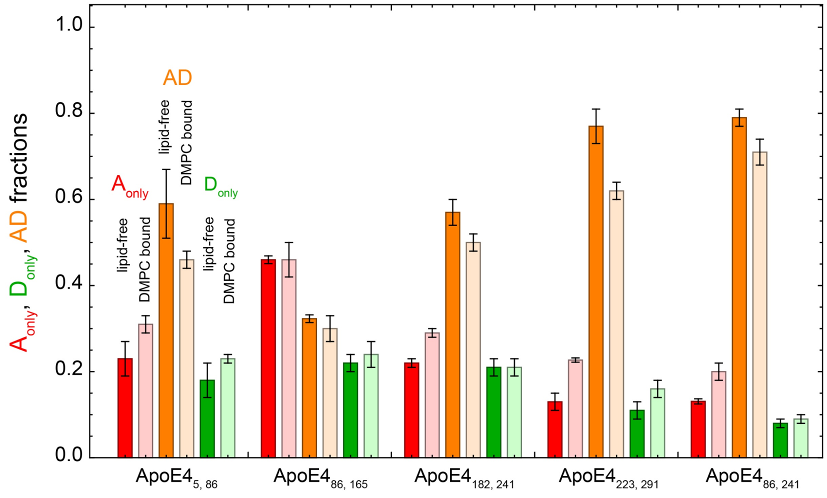

Figure S15: Fractions of acceptor only (red), donor only (green), and acceptor-donor (orange) labeled molecules as identified by Pulsed Interleaved Excitation (PIE) of lipid-free (dark shaded areas) and DMPC-bound (light shaded areas) full-length ApoE constructs. Error bars are standard deviations from multiple measurements (see **Table S13** for details). In all cases, binding to lipids results in a small decrease (up to about 15%) of the acceptor-donor labeled molecules often accompanied by a small increase in the fraction of donor and acceptor only population. These data support that only one labeled molecule of ApoE4 is binding to lipids since binding of two molecules would results in an increase of the donor-acceptor population.

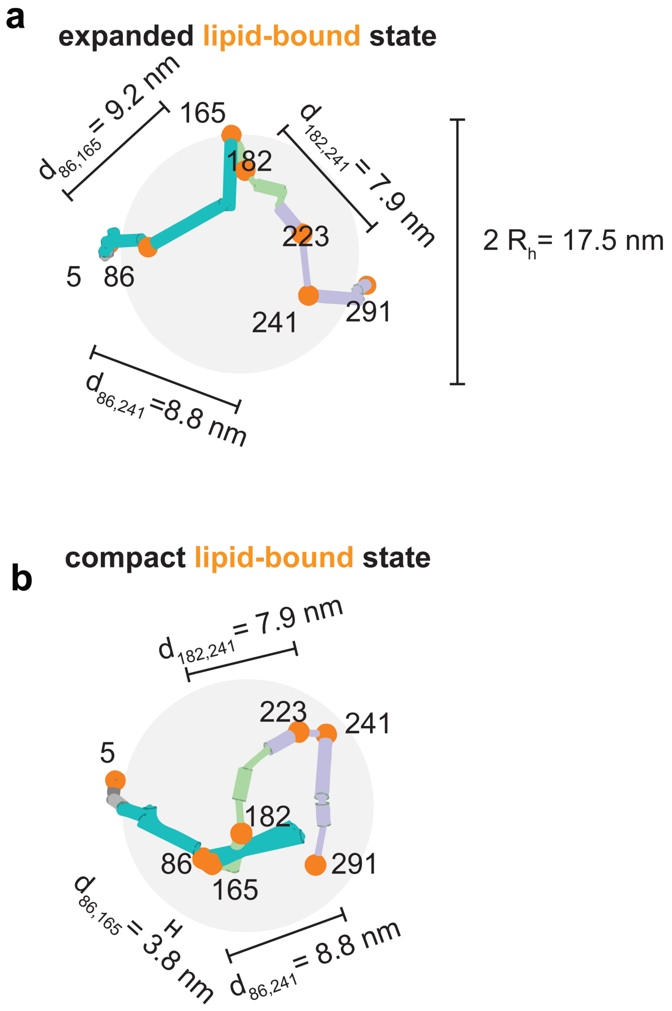

Figure S16: Estimation of distances for the lipid-bound state assuming a fixed distance is adopted.

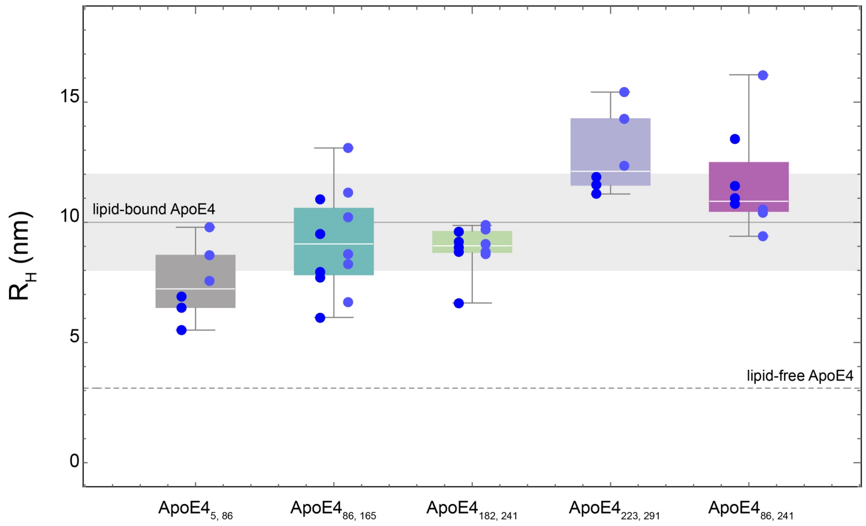

Figure S17: Hydrodynamic radii (R_H) of lipid-bound full-length ApoE4 constructs as measured by FCS. Mean R_H calculated from both donor-donor and acceptor$_{PIE}$-acceptor$_{PIE}$ correlations for each full-length construct assuming one diffusing species and two triplet components. Donor-donor and acceptor$_{PIE}$-acceptor$_{PIE}$ correlated data points are represented by dark and light blue dots, respectively. Box whisker plots report the median value as a white line, the 25% and 75% quantile, and the max and min values of the distribution for the combined donor-donor and acceptor$_{PIE}$-acceptor$_{PIE}$ datasets. Mean R_H values for lipid-bound (solid gray line) and lipid-free (dashed gray line) full length ApoE4 (compare with **Figure S18**).

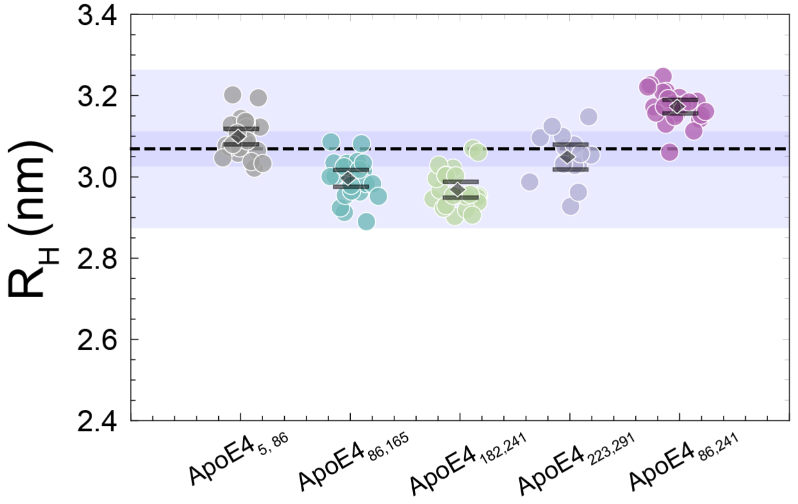

Figure S18: Hydrodynamic radii of lipid-free full-length ApoE4 constructs as measured by df-FCS assuming one diffusing species and two triplet components. Variations across each construct are within a 10% of relative error and they are indistinguishable from each other according to a one-tail t-student test with significance level ≤ 0.1. Black diamonds indicate the mean R_H value for each construct, and the error bars span the standard error of the mean. Dashed line denotes the overall weighted mean, and the darker shaded area spans the overall weighted SE of the mean –using individual $1/SE^2$ values as weights. Lighter shaded area denotes the overall 90% confidence interval around the overall weighted mean.

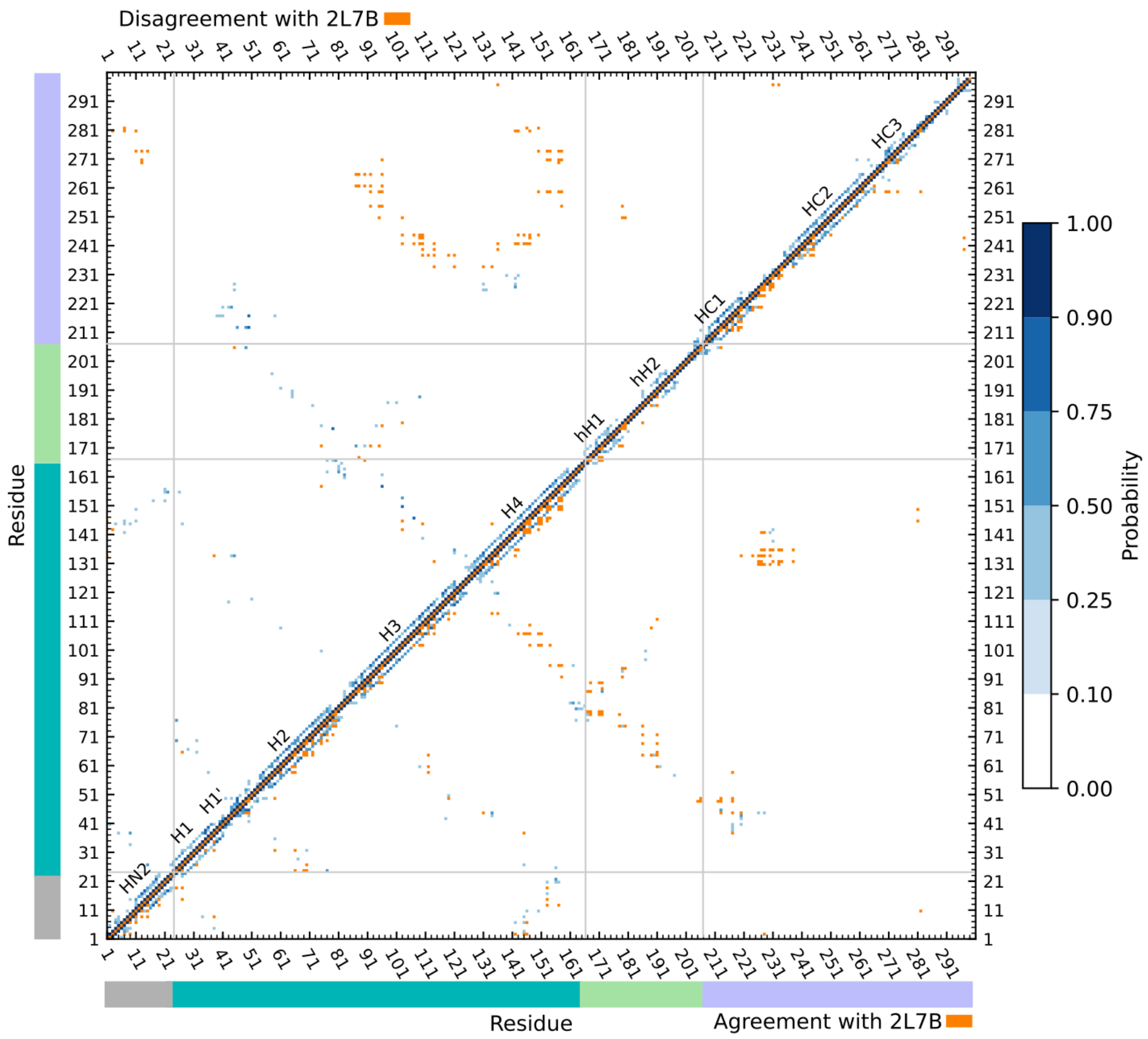

Figure S19. Comparison of Folding@home simulation results ApoE3-like NMR structure, 2L7B. Displayed are the probabilities of a contact (<3 Å between any heavy atom on the two sidechains) occurring between each pairwise residue in ApoE4 according to the Folding@home equilibrium probabilities. Above the diagonal, orange marks indicate a distance predicted in the ApoE3-like NMR structure, 2L7B, but are less than 10% likely in our simulations. Below the diagonal any contacts that occur in 2L7B and are more than 10% likely in our simulations are displayed in orange.

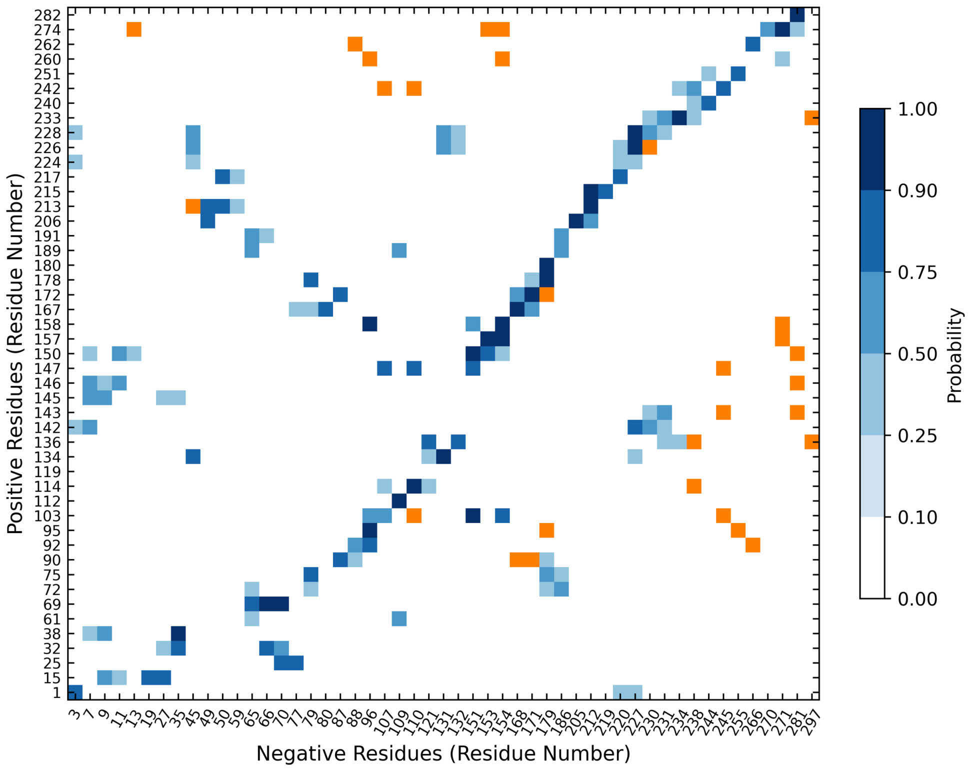

Figure S20. Predicted salt bridge interactions observed in our simulations. In blue are the equilibrium probabilities of any heavy atom in the residue sidechains being within 6Å of the other sidechain. In orange are interactions predicted by the ApoE3-like NMR structure, 2L7B, which are less than 10% likely in our simulations.

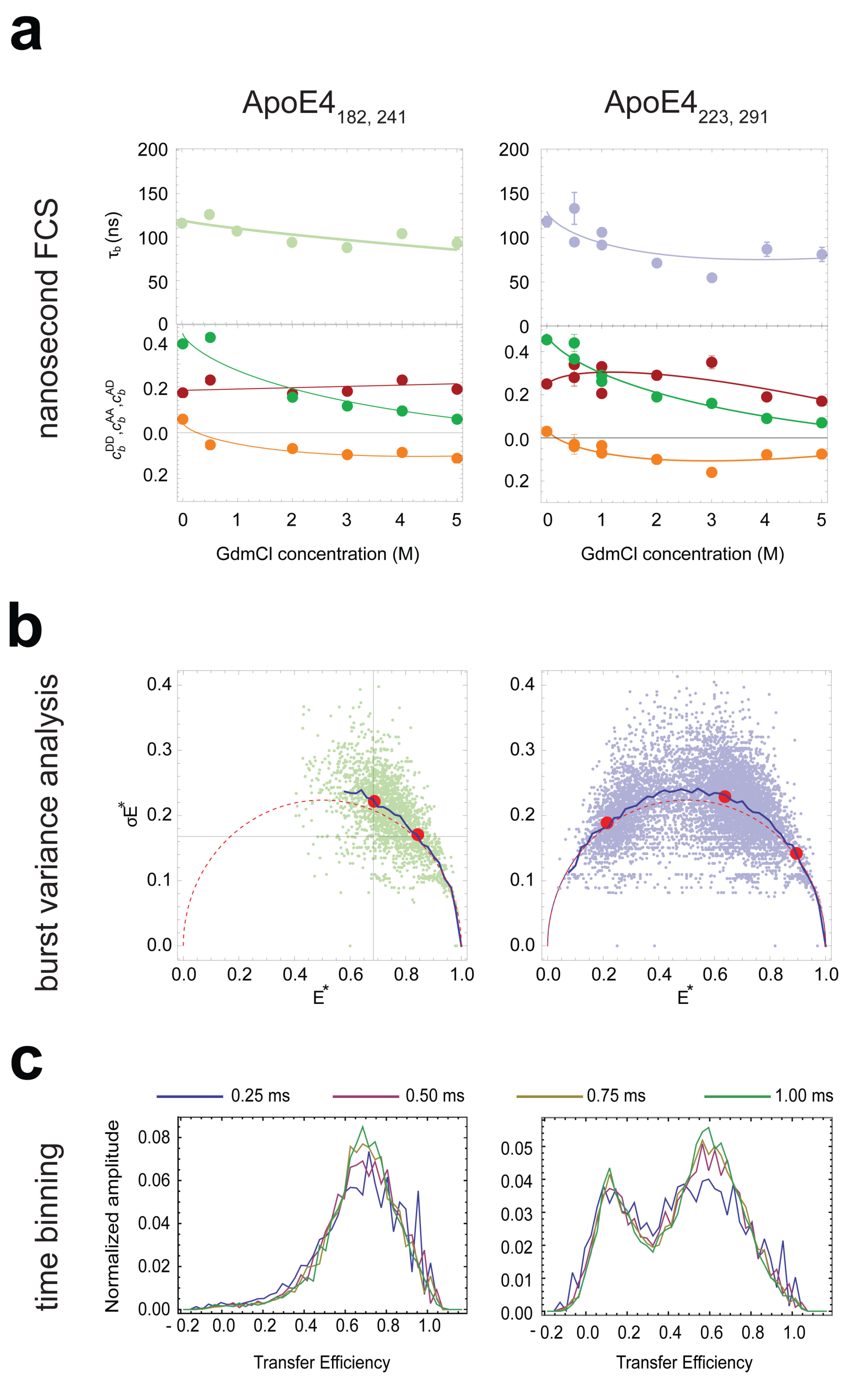

Figure S21. Dynamics in the C-terminal tail. a. Correlation time and amplitudes from global fit of nanosecond FCS data for **ApoE4$_{182,241}$** and **ApoE4$_{223,291}$**. The measurements highlight dynamics in the 100 ns timescale. Note that at 0M a positive amplitude is observed for the cross-correlation of both constructs suggesting a possible contribution of quenching of the fluorophores. The small effect disappears at 0.5 M GdmCl and higher

denaturant concentrations. The associated timescale appears on trend with the ones measured at 0 M GdmCl. Error bars represents the error of the fit. **b.** Burst variance analysis of **ApoE4$_{182,241}$** and **ApoE4$_{223,291}$**, as described in the **Supplementary Information**. Red dashed line represents the theoretical expectation of the standard deviation of the proximity ratio E*, blue solid line represents the mean value in proximity ration (binned in steps of 0.02), and red dots represents the mean value of the three major identified populations. Small deviations are observed for the low transfer efficiency peak of **ApoE4$_{182,241}$** and for the intermediate population of **ApoE4$_{223,291}$**, suggesting a possible contribution of dynamics in the microsecond timescale. **c.** Analysis of identified transfer efficiency distribution as function of different time binning choices, from 0.25 ms (blue), to 0.50 ms (magenta), 0.75 ms (yellow), and 1.00 ms (green). No significant variation is observed in the normalized histogram of transfer efficiencies suggesting that interconversion dynamics between the different states would occur on longer timescales, larger than 1 ms.

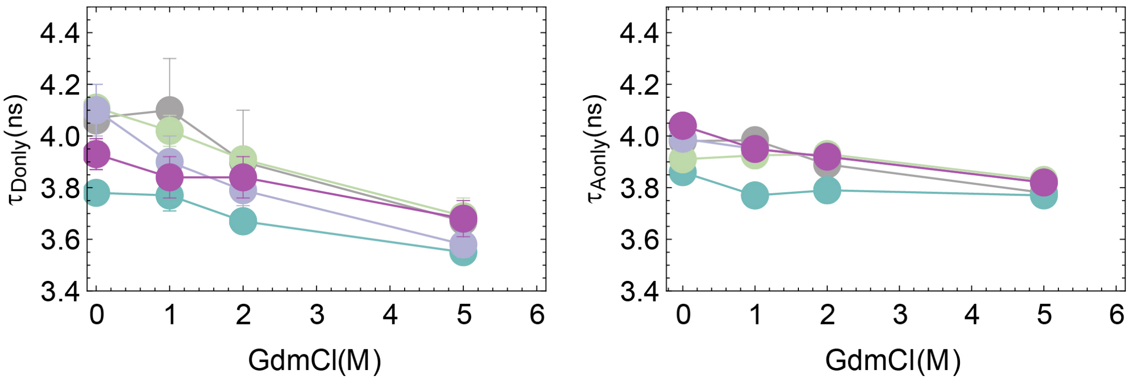

Figure S22: GdmCl dependent changes in donor-only and acceptor-only lifetimes. Error bars are standard deviation from multiple measurements (**Table S6**).

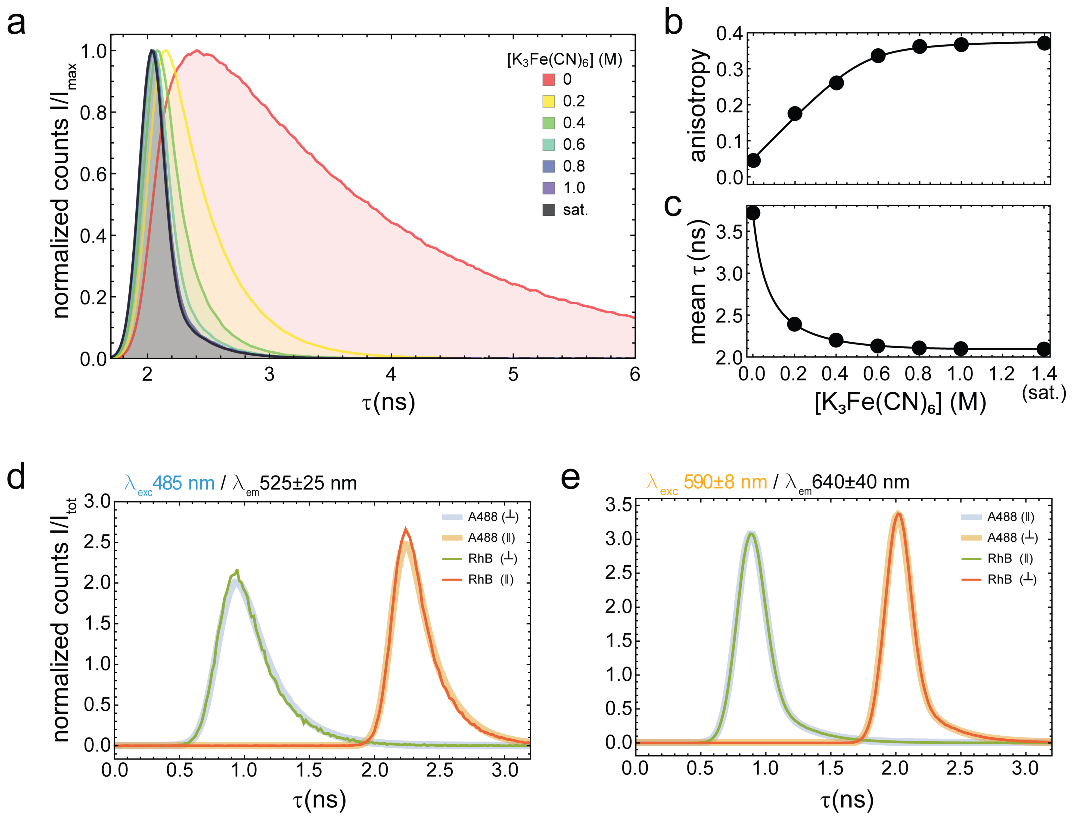

Figure S23: **Instrument Response Function (IRF) determination for donor and acceptor detection. a-c.** Fluorescence lifetime histogram of Rhodamine B in water with normalized counts against the maximum of the distribution (I/I_{max}) at different concentrations of Potassium Ferrocyanide (**a**) and corresponding steady-state anisotropies (**b**) and mean photon arrival time τ (**c**). **d-e**. Normalized fluorescence lifetime histograms of Alexa Fluor dyes and Rhodamine B in presence of saturated $K_3Fe(CN)_6$ with normalized counts against the total area I/I_{tot} for donor (**d**) and acceptor (**e**) excitations wavelengths.

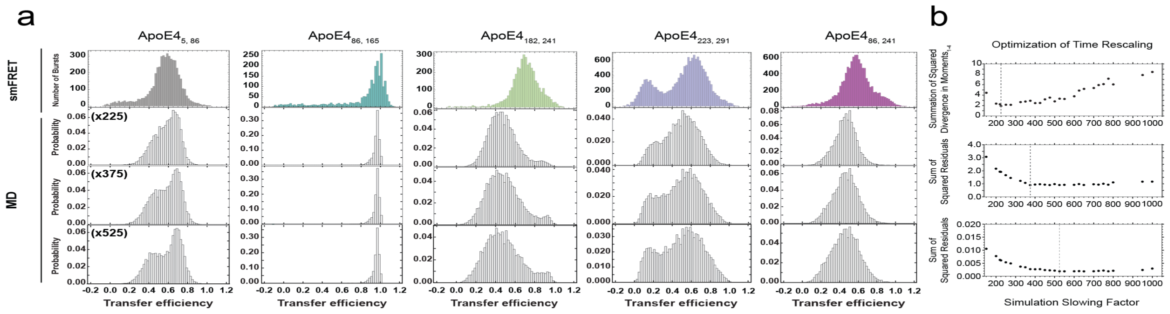

Figure S24. Optimization of time correction for simulations. a. Comparison between experimental and simulated transfer efficiency histograms with different time correction factors. **b.** The time correction factors in **a.** represent the minima obtained by optimizing the factor using the sum of the residuals for the first four moments of the distributions (upper panel, time factor equals to 225), the sum of the squared residuals across each histogram normalized by maximum excursion (central panel, time factor equals to 375), and the sum of the squared residuals for only the **ApoE4$_{223,291}$** construct (lower panel, time factor equals to 525).

Supplementary Tables

Table S1. Mean transfer efficiencies, widths, and relative areas obtained from the fit of lipid-free ApoE4 smFRET histograms. E_1, E_2, and **E_3** represent the mean value of transfer efficiencies for the each identified population in the transfer efficiency histograms determined at 0, 1, 2, and 5 M GdmCl (compare with **Fig. 2**) for lipid-free ApoE4. **W_1, W_2,** and **W_3** and **A_1, A_2,** and **A_3** reports about the corresponding width of the distribution and relative area. Errors are standard deviation of "n" independent repeats of the experiments.

**Lipid-free ApoE4$_{5, 86}$**										
**GdmCl** (M)		**E_1**	**E_2**	**E_3**	**W_1**	**W_2**	**W_3**	**A_1 (%)**	**A_2 (%)**	**A_3 (%)**
0	n=8	0.21±0.05	0.61±0.02	-	0.14±0.01	0.118±0.008	-	12±5	88±5	-
1	n=5	0.46±0.05	0.57±0.04	-	0.097±0.009	0.097±0.009	-	9±5	91±5	-
2*	n=5	-	0.5±0.1	-	-	0.113±0.006	-	-	100	-
5	n=5	-	0.23±0.05	-	-	0.074±0.003	-	-	100	-

* fit obtained assuming a single population for the main transfer efficiency

**Lipid-free ApoE4$_{86, 165}$**											
**GdmCl** (M)		**E_1**	**E_2**	**E_3**	**W_1**	**W_2**	**W_3**	**$Asym_3$**	**A_1 (%)**	**A_2 (%)**	**A_3 (%)**
0	n=4	0.345	0.62	0.98±0.01	0.10	0.10	0.14±0.03	0.84	5.6±0.2	9±2	85±2
1	n=5	0.345	0.608	0.978±0.005	0.10	0.10	0.15±0.01	0.84	6±2	12±3	82±6
2	n=8	0.41±0.02	0.63±0.02	0.973±0.005	0.099±0.003	0.10	0.15±0.02	0.84	51±10	24±3	26±10
5	n=6	0.23±0.02	-	-	0.076±0.009	-	-	-	100	-	-

**Lipid-free ApoE4$_{182, 241}$**										
**GdmCl** (M)		**E_1**	**E_2**	**E_3**	**W_1**	**W_2**	**W_3**	**A_1 (%)**	**A_2 (%)**	**A_3 (%)**
0	n=6	0.67±0.02	0.83±0.02	-	0.078	0.09	-	61 ± 6	38 ± 6	-
1	n=6	0.50±0.02	0.69±0.03	-	0.079	0.09	-	77 ± 6	23 ± 6	-
2	n=5	0.44±0.02	0.62±0.04	-	0.074±0.009	0.09	-	89 ± 1	11 ± 1	-
5	n=5	0.32±0.02	0.56±0.02	-	0.074±0.009	0.09	-	94 ± 3	6 ± 3	-

**Lipid-free ApoE4$_{223, 291}$**										
**GdmCl** (M)		**E_1**	**E_2**	**E_3**	**W_1**	**W_2**	**W_3**	**A_1 (%)**	**A_2 (%)**	**A_3 (%)**
0	n=12	0.13±0.04	0.61±0.02	0.851±0.003	0.105±0.004	0.13	0.104±0.001	27 ± 4	58 ± 4	15 ± 5
1	n=6	0.15±0.06	0.46±0.02	0.85	0.104	0.095±0.003	0.104	10 ± 6	85 ± 6	5 ± 1
2	n=6	-	0.37±0.02	-	-	0.093±0.009	-	-	100	-
5	n=5	-	0.27±0.02	-	-	0.079±0.006	-	-	100	-

**Lipid-free ApoE4$_{86, 241}$**

GdmCl (M)		E_1	E_2	E_3	W_1	W_2	W_3	A_1 (%)	A_2 (%)	A_3 (%)
0	n=5	0.24±0.01	0.59±0.02	0.87±0.02	0.12	0.113±0.007	0.09±0.01	7 ± 1	81 ± 1	12 ± 1
1	n=5	0.16±0.02	0.40±0.03	0.80	0.06±0.02	0.089±0.003	0.12	2.2 ± 0.3	92 ± 1	6 ± 1
2	n=4	-	0.25±0.04	-	-	0.106±0.008	-	-	100	-
5	n=4	-	0.10±0.03	-	-	0.06±0.01	-	-	100	-

Table S2. Folding free energy and midpoint of the transition determined from folding equilibrium analysis.

3-state model	
	$ApoE4_{86,165}$
$c_{1/2\ UN}$ (M)	1.91±0.015
ΔG^0_{UN} (RT)	-8.3±0.42
$c_{1/2\ NI}$ (M)	3±0.2
ΔG^0_{NI} (RT)	2.7±0.2

3-state model				
	$ApoE4_{5,86}$	$ApoE4_{182,241}$	$ApoE4_{223,291}$	$ApoE4_{86,241}$
$c_{1/2\ UN1}$ (M)	2.06±0.01	1.9±0.2	-0.8±0.1	-1.00±0.07
ΔG^0_{UN1} (RT)	-5.2±0.2	-8±7	1.15±0.08	1.47±0.04
$c_{1/2\ N1N2}$ (M)	-0.6±0.4	-0.3±0.1	1.40±0.09	-1.2±0.7
ΔG^0_{N1N2} (RT)	2.1±0.4	1.0±0.2	1.6±0.1	0.7±0.1

2-state model	
	$ApoE4_{5,86}$
$c_{1/2\ UI}$ (M)	2.07±0.03
ΔG^0_{UI} (RT)	-5.4±0.4
$c_{1/2\ N1N2}$ (M)	-0.41±0.06
ΔG^0_{N1N2} (RT)	1.76±0.08

Table S3. Folding of ApoE4 as reported in literature. $c_{1/2}$ and ΔG^0_{UN} previously reported in literature.

Region	Residues	$c_{1/2}$ (M)	ΔG^0_{UN} (kcal mol^{-1})	**reference**
Four-helix bundle	1-183	2	-	Weers et al. 2003[27]
Four-helix bundle	1-191	2	-	Morrow et al. 2000[25]
N-terminal domain	full-length	2.6 ± 1.3	5.25 ± 0.20	Dolai et al. [26]
C-terminal domain	full-length	0.73 ± 0.07	3.29 ± 0.20	Dolai et al. [26]
N-terminal domain (1-191)	fragment	-	4.56 ± 0.03	Dolai et al. [26]
C-terminal domain (192-299)	fragment	-	1.58 ± 0.08	Dolai et al. [26]

Table S4. Mean transfer efficiencies, widths, and relative areas obtained from the fit of lipid-free ApoE4 smFRET histograms. E_1, E_2, and **E_3** represent the mean value of transfer efficiencies for the each identified population in the transfer efficiency histograms determined at 0, 1, 2, and 5 M GdmCl (compare with **Fig. 7**.) for lipid-bound ApoE4. **W_1, W_2,** and **W_3** and **A_1, A_2,** and **A_3** reports about the corresponding width of the distribution and relative area. Errors are standard deviation of "n" independent repeats of the experiments.

Lipid-bound ApoE4										
**Construct**		**E_1**	**E_2**	**E_3**	**W_1**	**W_2**	**W_3**	**A_1 (%)**	**A_2 (%)**	**A_3 (%)**
5, 86	n=3	0.111±0.006	-	-	0.073±0.002	-	-	100	-	-
86, 165	n=6	0.037±0.006	0.894±0.004	-	0.17±0.01	0.080±0.009	-	51 ± 16	49 ± 16	-
182, 241	n=4	0.09±0.01 (1.72±0.07)	0.744	-	0.21±0.03	0.137	-	91 ± 2	9 ± 2	-
223, 291	n=3	0.091±0.008	-	-	0.072±0.002	-	-	100	-	-
86, 241	n=3	0.054±0.003 (1.57±0.04)	-	-	0.215±0.007	-	-	100	-	-

()=Asymmetry of lognormal distribution

Table S5. Correction parameters for anisotropy determinations. Anisotropy determination through confocal detection requires correction factors accounting for the detection efficiency on different parallel and perpendicular detectors (**G**-factor) as well as for scrambling of polarization due to tight focusing optics (L_p and L_s factors). Each parameter has been determined for Donor and Acceptor excitation and for corresponding detection.

**Excitation**	**Detection**	**L_s**	**L_p**	**G**
Donor	Acceptor	0.036	0.218	0.776
	Donor	0.072	0.126	0.992
Acceptor	Acceptor	0.042	0.169	1.29

Table S6. Subpopulation-specific analysis of time-resolved fluorescence decays for lipid-free ApoE4. Lifetime components obtained by fitting lifetime decays for donor only subpopulation, donor-acceptor population, and acceptor only population after PIE excitation. For each subpopulation, the lifetime component τ and the rotational component τ_{rot} are identified. From the comparison between the lifetime decay τ_{DA} decay and the lifetime of the donor in absence of acceptor, τ_{Donly} , we estimated the energy transfer characteristic time τ_{FRET}. Data are further subdivided based on the corresponding mean transfer efficiency (as identified in **Table S1**) and error are standard deviations of "n" independent repeats of the measurement.

ApoE4$_{5,\ 86}$

GdmCl (M)			τ_{Donly} (ns)	$\tau_{rot\text{-}Donly}$(ns)	τ_{DA}(ns)	$\tau_{rot\text{-}DA}$(ns)	τ_{FRET}(ns)	τ_{Aonly}(ns)	$\tau_{rot\text{-}Aonly}$(ns)
0	E_1	n=6	4.06±0.08	0.43±0.07	3.28±0.08	0.7±0.1	17±3	3.98±0.02	0.54±0.04
0	E_2	n=6	4.06±0.08	0.44±0.06	2.13±0.07	0.62±0.06	4.5±0.3	3.98±0.02	0.53±0.04
1	E_1	n=3	4.1±0.2	0.52±0.06	2.28±0.04	0.58±0.02	5.1±0.4	3.984±0.004	0.7±0.1
2	E_1	n=3	3.9±0.2	0.51±0.01	2.45±0.03	0.61±0.02	6.6±0.6	3.89±0.03	0.79±0.03
5	E_1	n=3	3.67±0.08	0.60±0.03	2.90±0.01	0.58±0.01	16±2	3.78±0.01	0.99±0.09

ApoE4$_{86,\ 165}$

GdmCl (M)			τ_{Donly} (ns)	$\tau_{rot\text{-}Donly}$(ns)	τ_{DA}(ns)	$\tau_{rot\text{-}DA}$(ns)	τ_{FRET}(ns)	τ_{Aonly}(ns)	$\tau_{rot\text{-}Aonly}$(ns)
0	E_3	n=3	3.78±0.03	0.32±0.04	0.68±0.05	0.225±0.008	0.83±0.07	3.86±0.01	0.40±0.01
1	E_3	n=3	3.77±0.06	0.48±0.02	0.82±0.02	0.30±0.01	1.05±0.03	3.77±0.02	0.51±0.02
1	E_2	n=3	3.77±0.06	0.48±0.02	2.68±0.01	0.62±0.09	9.3±0.4	3.77±0.02	0.51±0.02
2	E_3	n=2	3.67±0.02	0.61±0.03	0.86±0.09	0.29±0.01	1.1±0.2	3.79±0.02	0.75±0.08
2	E_2	n=2	3.67±0.02	0.61±0.03	2.30±0.04	0.578±0.009	6.2±0.3	3.79±0.02	0.75±0.08
2	E_1	n=2	3.67±0.02	0.61±0.03	2.782±0.007	0.64±0.02	11.5±0.2	3.79±0.02	0.75±0.08
5	E_1	n=3	3.55±0.03	0.724±0.009	3.030±0.006	0.56±0.01	21±1	3.77±0.01	1.03±0.05

ApoE4$_{182,\ 241}$

GdmCl (M)			τ_{Donly} (ns)	$\tau_{rot\text{-}Donly}$(ns)	τ_{DA}(ns)	$\tau_{rot\text{-}DA}$(ns)	τ_{FRET}(ns)	τ_{Aonly}(ns)	$\tau_{rot\text{-}Aonly}$(ns)
0	E_2	n=4	4.11±0.04	0.62±0.02	1.24±0.07	0.31±0.01	1.8±0.1	3.91±0.02	0.52±0.03
0	E_1	n=4	4.11±0.04	0.62±0.02	2.07±0.04	0.52±0.02	6.2±0.2	3.91±0.02	0.52±0.03
1	E_2	n=3	4.02±0.05	0.68±0.06	2.47±0.02	0.65±0.05	6.4±0.2	3.925±0.008	0.9±0.1
1	E_1	n=3	4.02±0.05	0.68±0.06	2.63±0.01	0.74±0.04	7.6±0.2	3.925±0.008	0.9±0.1
2	E_2	n=3	3.91±0.04	0.71±0.05	2.55±0.03	0.63±0.03	7.3±0.3	3.93±0.03	1.0±0.1
2	E_1	n=3	3.91±0.04	0.71±0.05	2.77±0.02	0.775±0.009	9.5±0.3	3.93±0.03	1.0±0.1
5	E_1	n=3	3.69±0.07	0.75±0.04	2.85±0.01	0.72±0.01	13±1	3.83±0.04	1.15±0.05

ApoE4$_{223,\ 291}$

GdmCl (M)			τ_{Donly} (ns)	$\tau_{rot\text{-}Donly}$(ns)	τ_{DA}(ns)	$\tau_{rot\text{-}DA}$(ns)	τ_{FRET}(ns)	τ_{Aonly}(ns)	$\tau_{rot\text{-}Aonly}$(ns)
0	E_3	n=4	4.1±0.1	0.6±0.2	1.59±0.08	0.40±0.04	2.6±0.2	3.99±0.02	0.68±0.02
0	E_2	n=4	4.1±0.1	0.6±0.2	2.33±0.01	0.59±0.03	5.4±0.2	3.99±0.02	0.68±0.02
0	E_1	n=4	4.1±0.1	0.6±0.2	3.35±0.02	0.6±0.1	18±2	3.99±0.02	0.68±0.02
1	E_2	n=3	3.9±0.1	0.7±0.1	2.65±0.03	0.74±0.02	8.3±0.5	3.95±0.02	0.92±0.03

2	E_2	n=3	3.79±0.06	0.60±0.05	2.793±0.006	0.744±0.007	10.6±0.5	3.92±0.01	0.99±0.02
5	E_2	n=3	3.58±0.05	0.69±0.01	2.92±0.02	0.71±0.02	16±1	3.82±0.03	1.2±0.1

**ApoE4$_{86, 241}$**									
**GdmCl** (M)			τ_{Donly} (ns)	$\tau_{rot\text{-}Donly}$(ns)	τ_{DA}(ns)	$\tau_{rot\text{-}DA}$(ns)	τ_{FRET}(ns)	τ_{Aonly}(ns)	$\tau_{rot\text{-}Aonly}$(ns)
0	E_3	n=3	3.93±0.06	0.51±0.08	1.33±0.04	0.37±0.03	2.01±0.09	4.04±0.01	0.34±0.01
0	E_2	n=3	3.93±0.06	0.51±0.08	2.45±0.01	0.64±0.03	6.5±0.2	4.04±0.01	0.34±0.01
0	E_1	n=3	3.93±0.06	0.51±0.08	3.30±0.04	0.70±0.02	21±2	4.04±0.01	0.34±0.01
1	E_2	n=3	3.84±0.08	0.53±0.04	2.92±0.02	0.63±0.02	12.2±0.6	3.95±0.02	0.8±0.2
2	E_2	n=3	3.84±0.08	0.56±0.02	3.18±0.01	0.65±0.02	18.5±1.9	3.92±0.01	0.89±0.06
5	E_2	n=3	3.68±0.07	0.537±0.005	3.32±0.02	0.64±0.01	34±6	3.82±0.03	0.95±0.03

Table S7. Subpopulation-specific analysis of time-resolved fluorescence decays for lipid-bound ApoE4. Lifetime components obtained by fitting lifetime decays for donor only subpopulation, donor-acceptor population, and acceptor only population after PIE excitation. For each subpopulation, the lifetime component τ and the rotational component τ_{rot} are identified. From the comparison between the lifetime decay τ_{DA} decay and the lifetime of the donor in absence of acceptor, τ_{Donly}, we estimated the energy transfer characteristic time τ_{FRET}. Data are further subdivided based on the corresponding mean transfer efficiency (as identified in **Table S4**) and error are standard deviations of “n” independent repeats of the measurement.

**Construct**	**E**		τ_{Donly} (ns)	$\tau_{rot\text{-}Donly}$(ns)	τ_{DA}(ns)	$\tau_{rot\text{-}DA}$(ns)	τ_{FRET}(ns)	τ_{Aonly}(ns)	$\tau_{rot\text{-}Aonly}$(ns)
ApoE4$_{5, 86}$	E_1	n=3	4.2±0.2	0.31±0.02	3.72±0.02	0.43±0.01	33±12	4.01±0.07	0.46±0.07
ApoE4$_{86, 165}$	E_1	n=5	4.06±0.05	0.33±0.02	3.89±0.04	0.33±0.03	93±35	4.04±0.06	0.29±0.06
ApoE4$_{86, 165}$	E_2	n=5	4.06±0.05	0.33±0.02	1.24±0.06	0.31±0.02	1.79±0.12	4.04±0.06	0.29±0.06
ApoE4$_{182, 241}$	E_1	n=4	4.13±0.06	0.29±0.02	3.64±0.04	0.34±0.03	31±4	4.07±0.06	0.33±0.04
ApoE4$_{182, 241}$	E_2	n=4	4.13±0.06	0.29±0.02	2.6±0.1	0.40±0.02	7.0±0.7	4.07±0.06	0.33±0.04
ApoE4$_{223, 291}$	E_1	n=3	4.21±0.09	0.30±0.01	3.67±0.02	0.37±0.03	29±4	4.27±0.03	0.43±0.03
ApoE4$_{86, 241}$	E_1	n=4	4.08±0.04	0.31±0.04	3.8±0.1	0.37±0.04	52±25	4.10±0.04	0.16±0.04

Table S8. Steady-state and time resolved anisotropies in aqueous buffer conditions. Steady-state anisotropy $\mathbf{r_{ss}}$, $\mathbf{r_\infty}$ and rotational component τ_{rot} for donor-only, acceptor-only, and acceptor-donor subpopulations (compare **Eq. S10** and corresponding section). Errors represent standard deviations from “n” independent repeats of the measurement.

**Construct**	**E**		$r_{ss\text{-}Donly}$	$r_{\infty\text{-}Donly}$	$\tau_{rot\text{-}Donly}$ **(ns)**	$r_{ss\text{-}Aonly}$	$r_{\infty\text{-}Aonly}$	$\tau_{rot\text{-}Aonly}$ **(ns)**	$r_{ss\text{-}A(D)}$	$r_{\infty\text{-}A(D)}$
ApoE4$_{5, 86}$	E_2	n=6	0.09±0.01	0.09±0.02	0.47±0.04	0.08±0.01	0.07±0.03	0.45±0.06	0.062±0.004	0.06±0.01
ApoE4$_{5, 86}$	E_1	n=6	0.09±0.01	0.09±0.02	0.47±0.04	0.08±0.01	0.07±0.03	0.45±0.06	0.087±0.007	0.10±0.02
ApoE4$_{86, 165}$	E_3	n=5	0.136±0.003	0.143±0.009	0.36±0.06	0.097±0.005	0.102±0.003	0.37±0.01	0.049±0.003	0.043±0.006
ApoE4$_{182, 241}$	E_2	n=4	0.131±0.005	0.15±0.01	0.50±0.06	0.099±0.009	0.10±0.01	0.43±0.04	0.060±0.006	0.061±0.005
ApoE4$_{182, 241}$	E_1	n=4	0.131±0.005	0.15±0.01	0.50±0.06	0.099±0.009	0.10±0.01	0.43±0.04	0.057±0.003	0.064±0.004
ApoE4$_{223, 291}$	E_3	n=4	0.13±0.02	0.16±0.02	0.48±0.06	0.13±0.01	0.146±0.009	0.50±0.05	0.065±0.004	0.069±0.009
ApoE4$_{223, 291}$	E_2	n=4	0.13±0.02	0.16±0.02	0.48±0.06	0.13±0.01	0.146±0.009	0.50±0.05	0.063±0.001	0.060±0.004
ApoE4$_{223, 291}$	E_1	n=4	0.13±0.02	0.16±0.02	0.48±0.06	0.13±0.01	0.146±0.009	0.50±0.05	0.112±0.007	0.15±0.02
ApoE4$_{86, 241}$	E_3	n=4	0.142±0.007	0.146±0.003	0.54±0.01	0.142±0.003	0.148±0.007	0.42±0.05	0.063±0.003	0.058±0.006
ApoE4$_{86, 241}$	E_2	n=4	0.142±0.007	0.146±0.003	0.54±0.01	0.142±0.003	0.148±0.007	0.42±0.05	0.0604±0.0007	0.069±0.003
ApoE4$_{86, 241}$	E_1	n=4	0.142±0.007	0.146±0.003	0.54±0.01	0.142±0.003	0.148±0.007	0.42±0.05	0.080±0.004	0.08±0.01

Table S9. Steady-state and time resolved anisotropies for lipid-bound ApoE4. Steady-state anisotropy r_{ss}, r_{∞} and rotational component τ_{rot} for donor-only, acceptor-only, and acceptor-donor subpopulations (compare **Eq. S10** and corresponding section). Errors represent standard deviations from "n" independent repeats of the measurement.

Construct	E		$r_{ss\text{-}Donly}$	$r_{\infty\text{-}Donly}$	$\tau_{rot\text{-}Donly}$ (ns)	$r_{ss\text{-}Aonly}$	$r_{\infty\text{-}Aonly}$	$\tau_{rot\text{-}Aonly}$ (ns)	$r_{ss\text{-}A(D)}$	$r_{\infty\text{-}A(D)}$
$ApoE4_{5,\ 86}$	E_1	n=3	0.078±0.002	0.10±0.02	0.34±0.02	0.07±0.01	0.053±0.007	0.66±0.02	0.121±0.002	0.14±0.01
$ApoE4_{86,\ 165}$	E_1	n=5	0.16±0.01	0.149±0.009	0.64±0.09	0.10±0.02	0.11±0.03	0.57±0.02	0.17±0.01	0.24±0.03
$ApoE4_{86,\ 165}$	E_2	n=5	0.16±0.01	0.149±0.009	0.64±0.09	0.10±0.02	0.11±0.03	0.57±0.02	0.050±0.006	0.051±0.004
$ApoE4_{182,\ 241}$	E_1	n=4	0.134±0.009	0.14±0.01	0.47±0.08	0.139±0.006	0.147±0.009	0.71±0.04	0.131±0.003	0.17±0.03
$ApoE4_{182,\ 241}$	E_2	n=4	0.134±0.009	0.14±0.01	0.47±0.08	0.139±0.006	0.147±0.009	0.71±0.04	0.083±0.006	0.085±0.004
$ApoE4_{223,\ 291}$	E_1	n=3	0.13±0.01	0.15±0.01	0.44±0.04	0.14±0.01	0.16±0.01	0.75±0.04	0.141±0.002	0.208±0.009
$ApoE4_{86,\ 241}$	E_1	n=4	0.14±0.02	0.14±0.02	0.54±0.06	0.17±0.02	0.20±0.01	0.61±0.04	0.152±0.003	0.22±0.01

Table S10. Estimates of deviations in the orientational κ^2 factor in aqueous buffer conditions. See corresponding section in the Supplementary Information for definition of the different terms.

Construct	E	κ^2_{min}	κ^2_{max}	κ^2_{mean}	precision	accuracy	$R_{0,mean}$
$ApoE4_{5,\ 86}$	E_2	0.36 (0.35)	1.75 (1.77)	0.79 (0.79)	0.01 (0.01)	0.06 (0.07)	5.55
$ApoE4_{5,\ 86}$	E_1	0.36 (0.35)	1.75 (1.77)	0.88 (0.91)	0.03 (0.03)	0.07 (0.07)	5.66
$ApoE4_{86,\ 165}$	E_3	0.29 (0.30)	2.17 (2.12)	0.73 (0.74)	0.006 (0.002)	0.07 (0.07)	5.48
$ApoE4_{182,\ 241}$	E_2	0.29 (0.30)	2.22 (2.09)	0.76 (0.76)	0.0002 (0.002)	0.07 (0.07)	5.52
$ApoE4_{182,\ 241}$	E_1	0.29 (0.30)	2.22 (2.09)	0.77 (0.76)	0.0008 (0.0008)	0.07 (0.07)	5.53
$ApoE4_{223,\ 291}$	E_3	0.24 (0.28)	2.35 (2.13)	0.76 (0.76)	0.005 (0.0006)	0.08 (0.08)	5.52
$ApoE4_{223,\ 291}$	E_2	0.24 (0.28)	2.35 (2.13)	0.75 (0.76)	0.008 (0.001)	0.08 (0.08)	5.51
$ApoE4_{223,\ 291}$	E_1	0.24 (0.28)	2.35 (2.13)	0.98 (0.88)	0.03 (0.02)	0.1 (0.08)	5.76
$ApoE4_{86,\ 241}$	E_3	0.25 (0.26)	2.26 (2.23)	0.74 (0.76)	0.007 (0.004)	0.08 (0.08)	5.49
$ApoE4_{86,\ 241}$	E_2	0.25 (0.26)	2.26 (2.23)	0.76 (0.75)	0.004 (0.005)	0.08 (0.08)	5.52
$ApoE4_{86,\ 241}$	E_1	0.25 (0.26)	2.26 (2.23)	0.78 (0.79)	0.0004 (0.0009)	0.08 (0.08)	5.54

Table S11. Estimates of deviations in the orientational κ^2 factor for lipid-bound ApoE4. See corresponding section in the Supplementary Information for definition of the different terms.

Construct	E	κ^2_{min}	κ^2_{max}	κ^2_{mean}	precision	accuracy	$R_{0,mean}$
$ApoE4_{5,\ 86}$	E_1	0.37 (0.37)	1.78 (1.66)	0.85 (0.86)	0.03 (0.03)	0.05 (0.06)	5.62
$ApoE4_{86,\ 165}$	E_1	0.28 (0.28)	2.23 (2.28)	0.92 (0.95)	0.03 (0.03)	0.07 (0.08)	5.70
$ApoE4_{86,\ 165}$	E_2	0.28 (0.28)	2.23 (2.28)	0.74 (0.74)	0.005 (0.005)	0.07 (0.07)	5.49
$ApoE4_{182,\ 241}$	E_1	0.26 (0.26)	2.16 (2.21)	0.98 (0.92)	0.03 (0.02)	0.09 (0.09)	5.76
$ApoE4_{182,\ 241}$	E_2	0.26 (0.26)	2.22 (2.21)	0.80 (0.79)	0.002 (0.003)	0.08 (0.08)	5.56
$ApoE4_{223,\ 291}$	E_1	0.24 (0.27)	2.30 (2.15)	0.99 (0.99)	0.03 (0.03)	0.09 (0.09)	5.77
$ApoE4_{86,\ 241}$	E_1	0.22 (0.24)	2.29 (2.25)	1.01 (0.99)	0.03 (0.03)	0.10 (0.10)	5.79

()=values estimated from steady-state anisotropies.

Table S12. σ^2 values (Eq. 18) for ApoE4 under aqueous buffer and lipid-bound conditions. Comparison of σ^2 computed with Eq. 18 between measured values and estimates from Gaussian distribution. The Gaussian distribution serves as a reference for a completely disordered and highly dynamic ensemble.

Construct			$E_{1\text{-Measured}}$	$E_{1\text{-Gaussian}}$	$E_{2\text{-Measured}}$	$E_{2\text{-Gaussian}}$	$E_{3\text{-Measured}}$	$E_{3\text{-Gaussian}}$
$ApoE4_{5, 86}$	lipid-free	n=6	0.02±0.01	0.08±0.02	0.055±0.007	0.107±0.004	-	-
	DMPC	n=3	0.01±0.01	0.050±0.002	-	-	-	-
$ApoE4_{86, 165}$	lipid-free	n=3	0.14±0.03	0.115	0.09±0.01	0.107	0.0010±0.0006	0.0003±0.0004
	DMPC	n=6	0.003±0.003	0.017±0.003	0.021±0.001	0.028±0.001	-	-
$ApoE4_{182, 241}$	lipid-free	n=4	0.061±0.002	0.089±0.002	0.022±0.003	0.035±0.007	-	-
	DMPC	n=4	0	0.039±0.005	0.095±0.005	0.0769	-	-
$ApoE4_{223, 291}$	lipid-free	n=4	0.01±0.01	0.0761±0.0003	0.075±0.007	0.104±0.001	0.035±0.004	0.040±0.004
	DMPC	n=3	0	0.041±0.003	-	-	-	-
$ApoE4_{86, 241}$	lipid-free	n=3	0.07±0.01	0.097	0.091±0.003	0.1094±0.0003	0.0255±0.0009	0.0297±0.0007
	DMPC	n=3	0	0.025±0.001	-	-	-	-

Table S13. Fraction of molecules corresponding to acceptor only Aonly, donor-acceptor (DA) and donor only (D_{only}) for ApoE4 under aqueous buffer and lipid-bound conditions. All errors represent standard deviations based on n independent replicates of the experiments.

Construct			A_{only}	DA	D_{only}
$ApoE4_{5, 86}$	lipid-free	n=6	0.23±0.04	0.59±0.08	0.18±0.04
	DMPC	n=3	0.31±0.02	0.46±0.02	0.23±0.01
$ApoE4_{86, 165}$	lipid-free	n=3	0.460±0.009	0.323±0.009	0.22±0.02
	DMPC	n=6	0.46±0.04	0.30±0.03	0.24±0.03
$ApoE4_{182, 241}$	lipid-free	n=4	0.22±0.01	0.57±0.03	0.21±0.02
	DMPC	n=4	0.29±0.01	0.50±0.02	0.21±0.02
$ApoE4_{223, 291}$	lipid-free	n=4	0.13±0.02	0.77±0.04	0.11±0.02
	DMPC	n=3	0.227±0.005	0.62±0.02	0.16±0.02
$ApoE4_{86, 241}$	lipid-free	n=3	0.131±0.006	0.79±0.02	0.08±0.01
	DMPC	n=3	0.20±0.02	0.71±0.03	0.09±0.01

Table S14. Analysis of Fluorescence Correlation Spectroscopy under lipid-bound conditions. Hydrodynamic radius $\mathbf{R_h}$ of ApoE4 constructs bound to DMPC liposomes as determined by donor-donor (DD) and acceptor-acceptor (AA) correlation. **N** reports about the corresponding number of molecules as determined by the amplitude of the correlation. All errors represent standard deviations based on "n" independent replicates of the experiments.

Lipid-bound (DMPC) ApoE4 constructs					
**Construct**	$\mathbf{R_h^{DD}}$**(nm)**	$\mathbf{R_h^{AA}}$**(nm)**	$\mathbf{N^{DD}}$	$\mathbf{N^{AA}}$	
$ApoE4_{5,\ 86}$	6.3±0.7	9±1	0.4±0.1	0.13±0.02	n=3
$ApoE4_{86,\ 165}$	9±2	10±2	0.25±0.04	0.097±0.003	n=6
$ApoE4_{182,\ 241}$	9±1	9.2±0.5	0.3±0.1	0.100±0.006	n=4
$ApoE4_{223,\ 291}$	11.5±0.4	14±2	0.27±0.04	0.109±0.009	n=4
$ApoE4_{86,\ 241}$	12±1	12±3	0.212±0.009	0.17±0.04	n=3

Table S15. Residue pairs used for computing distances used in clustering protein conformations.

Residue pairs		
5,86	48,129	32,67
86,165	67,149	108,149
182,241	129,234	32,149
223,291	32,108	67,108
86,241	191,215	11,280

REFERENCES

s

1 Mahley, R. W. Apolipoprotein E: from cardiovascular disease to neurodegenerative disorders. *J Mol Med (Berl)* **94**, 739-746, doi:10.1007/s00109-016-1427-y (2016).

2 Verghese, P. B., Castellano, J. M. & Holtzman, D. M. Apolipoprotein E in Alzheimer's disease and other neurological disorders. *Lancet Neurol* **10**, 241-252, doi:10.1016/S1474-4422(10)70325-2 (2011).

3 Zannis, V. I. *et al.* Proposed nomenclature of apoE isoproteins, apoE genotypes, and phenotypes. *J Lipid Res* **23**, 911-914 (1982).

4 Weisgraber, K. H., Rall, S. C., Jr. & Mahley, R. W. Human E apoprotein heterogeneity. Cysteine-arginine interchanges in the amino acid sequence of the apo-E isoforms. *J Biol Chem* **256**, 9077-9083 (1981).

5 Saunders, A. M. *et al.* Association of apolipoprotein E allele epsilon 4 with late-onset familial and sporadic Alzheimer's disease. *Neurology* **43**, 1467-1472 (1993).

6 Corder, E. H. *et al.* Gene dose of apolipoprotein E type 4 allele and the risk of Alzheimer's disease in late onset families. *Science* **261**, 921-923 (1993).

7 Strittmatter, W. J. *et al.* Apolipoprotein E: high-avidity binding to beta-amyloid and increased frequency of type 4 allele in late-onset familial Alzheimer disease. *Proceedings of the National Academy of Sciences of the United States of America* **90**, 1977-1981 (1993).

8 Roses, A. D. Apolipoprotein E alleles as risk factors in Alzheimer's disease. *Annual review of medicine* **47**, 387-400, doi:10.1146/annurev.med.47.1.387 (1996).

9 Liu, C. C., Liu, C. C., Kanekiyo, T., Xu, H. & Bu, G. Apolipoprotein E and Alzheimer disease: risk, mechanisms and therapy. *Nature reviews. Neurology* **9**, 106-118, doi:10.1038/nrneurol.2012.263 (2013).

10 Yamazaki, Y., Zhao, N., Caulfield, T. R., Liu, C. C. & Bu, G. Apolipoprotein E and Alzheimer disease: pathobiology and targeting strategies. *Nat Rev Neurol* **15**, 501-518, doi:10.1038/s41582-019-0228-7 (2019).

11 Neu, S. C. *et al.* Apolipoprotein E Genotype and Sex Risk Factors for Alzheimer Disease: A Meta-analysis. *JAMA Neurol* **74**, 1178-1189, doi:10.1001/jamaneurol.2017.2188 (2017).

12 Li, Z., Shue, F., Zhao, N., Shinohara, M. & Bu, G. APOE2: protective mechanism and therapeutic implications for Alzheimer's disease. *Mol Neurodegener* **15**, 63, doi:10.1186/s13024-020-00413-4 (2020).

13 Mahley, R. W., Weisgraber, K. H. & Huang, Y. Apolipoprotein E4: a causative factor and therapeutic target in neuropathology, including Alzheimer's disease. *Proc Natl Acad Sci U S A* **103**, 5644-5651, doi:10.1073/pnas.0600549103 (2006).

14 Shi, Y. *et al.* ApoE4 markedly exacerbates tau-mediated neurodegeneration in a mouse model of tauopathy. *Nature* **549**, 523-527, doi:10.1038/nature24016 (2017).

15 Dong, L. M. & Weisgraber, K. H. Human apolipoprotein E4 domain interaction. Arginine 61 and glutamic acid 255 interact to direct the preference for very low density lipoproteins. *J Biol Chem* **271**, 19053-19057, doi:10.1074/jbc.271.32.19053 (1996).

16 Garai, K., Baban, B. & Frieden, C. Dissociation of apolipoprotein E oligomers to monomer is required for high-affinity binding to phospholipid vesicles. *Biochemistry* **50**, 2550-2558, doi:10.1021/bi1020106 (2011).

17 Liao, F. *et al.* Targeting of nonlipidated, aggregated apoE with antibodies inhibits amyloid accumulation. *J Clin Invest* **128**, 2144-2155, doi:10.1172/JCI96429 (2018).

18 Garai, K. & Frieden, C. The association-dissociation behavior of the ApoE proteins: kinetic and equilibrium studies. *Biochemistry* **49**, 9533-9541, doi:10.1021/bi101407m (2010).

19 Frieden, C., Wang, H. & Ho, C. M. W. A mechanism for lipid binding to apoE and the role of intrinsically disordered regions coupled to domain-domain interactions. *Proc Natl Acad Sci U S A* **114**, 6292-6297, doi:10.1073/pnas.1705080114 (2017).

20 Wilson, C., Wardell, M. R., Weisgraber, K. H., Mahley, R. W. & Agard, D. A. Three-dimensional structure of the LDL receptor-binding domain of human apolipoprotein E. *Science* **252**, 1817-1822, doi:10.1126/science.2063194 (1991).

21 Dong, J. *et al.* Interaction of the N-terminal domain of apolipoprotein E4 with heparin. *Biochemistry-Us* **40**, 2826-2834 (2001).

22 Chen, J., Li, Q. & Wang, J. Topology of human apolipoprotein E3 uniquely regulates its diverse biological functions. *Proc Natl Acad Sci U S A* **108**, 14813-14818, doi:10.1073/pnas.1106420108 (2011).

23 Weisgraber, K. H. Apolipoprotein-E - Structure-Function-Relationships. *Adv Protein Chem* **45**, 249-302 (1994).

24 Hatters, D. M., Budamagunta, M. S., Voss, J. C. & Weisgraber, K. H. Modulation of apolipoprotein E structure by domain interaction: differences in lipid-bound and lipid-free forms. *J Biol Chem* **280**, 34288-34295, doi:10.1074/jbc.M506044200 (2005).

25 Morrow, J. A. *et al.* Differences in stability among the human apolipoprotein E isoforms determined by the amino-terminal domain. *Biochemistry* **39**, 11657-11666, doi:10.1021/bi000099m (2000).

26 Dolai, S., Cherakara, S. & Garai, K. Apolipoprotein E4 exhibits intermediates with domain interaction. *Biochim Biophys Acta Proteins Proteom* **1868**, 140535, doi:10.1016/j.bbapap.2020.140535 (2020).

27 Weers, P. M. *et al.* Lipid binding ability of human apolipoprotein E N-terminal domain isoforms: correlation with protein stability? *Biophys Chem* **100**, 481-492, doi:10.1016/s0301-4622(02)00300-9 (2003).

28 Hatters, D. M., Voss, J. C., Budamagunta, M. S., Newhouse, Y. N. & Weisgraber, K. H. Insight on the molecular envelope of lipid-bound apolipoprotein E from electron paramagnetic resonance spectroscopy. *J Mol Biol* **386**, 261-271, doi:10.1016/j.jmb.2008.12.040 (2009).

29 Henry, N. *et al.* Lipidated apolipoprotein E4 structure and its receptor binding mechanism determined by a combined cross-linking coupled to mass spectrometry and molecular dynamics approach. *PLoS Comput Biol* **14**, e1006165, doi:10.1371/journal.pcbi.1006165 (2018).

30 Alston, J. J., Soranno, A. & Holehouse, A. S. Integrating single-molecule spectroscopy and simulations for the study of intrinsically disordered proteins. *Methods* **193**, 116-135, doi:10.1016/j.ymeth.2021.03.018 (2021).

31 Dong, L. M. *et al.* Human apolipoprotein E. Role of arginine 61 in mediating the lipoprotein preferences of the E3 and E4 isoforms. *J Biol Chem* **269**, 22358-22365 (1994).

32 Weisgraber, K. H. Apolipoprotein E: structure-function relationships. *Adv Protein Chem* **45**, 249-302, doi:10.1016/s0065-3233(08)60642-7 (1994).

33 Petrlova, J. *et al.* A differential association of Apolipoprotein E isoforms with the amyloid-beta oligomer in solution. *Proteins* **79**, 402-416, doi:10.1002/prot.22891 (2011).

34 Gupta, V. *et al.* Lipid-induced extension of apolipoprotein E helix 4 correlates with low density lipoprotein receptor binding ability. *J Biol Chem* **281**, 39294-39299, doi:10.1074/jbc.M608085200 (2006).

35 den Hartigh, L. J. *et al.* Postprandial apoE isoform and conformational changes associated with VLDL lipolysis products modulate monocyte inflammation. *Plos One* **7**, e50513, doi:10.1371/journal.pone.0050513 (2012).
36 Bentley, N. M., Ladu, M. J., Rajan, C., Getz, G. S. & Reardon, C. A. Apolipoprotein E structural requirements for the formation of SDS-stable complexes with beta-amyloid-(1-40): the role of salt bridges. *Biochem J* **366**, 273-279, doi:10.1042/BJ20020207 (2002).
37 Dashti, M. *et al.* A Phospholipidomic Analysis of All Defined Human Plasma Lipoproteins. *Sci Rep-Uk* **1**, doi:ARTN 139
10.1038/srep00139 (2011).
38 Fonteh, A. N., Cipolla, M., Chiang, J. R., Arakaki, X. H. & Harrington, M. G. Human Cerebrospinal Fluid Fatty Acid Levels Differ between Supernatant Fluid and Brain-Derived Nanoparticle Fractions, and Are Altered in Alzheimer's Disease. *Plos One* **9**, doi:ARTN e100519
10.1371/journal.pone.0100519 (2014).
39 Segrest, J. P. *et al.* The amphipathic helix in the exchangeable apolipoproteins: a review of secondary structure and function. *J Lipid Res* **33**, 141-166 (1992).
40 Peters-Libeu, C. A., Newhouse, Y., Hall, S. C., Witkowska, H. E. & Weisgraber, K. H. Apolipoprotein E*dipalmitoylphosphatidylcholine particles are ellipsoidal in solution. *J Lipid Res* **48**, 1035-1044, doi:10.1194/jlr.M600545-JLR200 (2007).
41 Schneeweis, L. A., Koppaka, V., Lund-Katz, S., Phillips, M. C. & Axelsen, P. H. Structural analysis of lipoprotein E particles. *Biochemistry* **44**, 12525-12534, doi:10.1021/bi050872j (2005).
42 Segall, M. L. *et al.* Influence of apoE domain structure and polymorphism on the kinetics of phospholipid vesicle solubilization. *J Lipid Res* **43**, 1688-1700, doi:10.1194/jlr.m200157-jlr200 (2002).
43 Zhukovsky, M. A., Filograna, A., Luini, A., Corda, D. & Valente, C. Protein Amphipathic Helix Insertion: A Mechanism to Induce Membrane Fission. *Front Cell Dev Biol* **7**, 291, doi:10.3389/fcell.2019.00291 (2019).
44 Gallop, J. L. *et al.* Mechanism of endophilin N-BAR domain-mediated membrane curvature. *EMBO J* **25**, 2898-2910, doi:10.1038/sj.emboj.7601174 (2006).
45 Miller, S. E. *et al.* CALM regulates clathrin-coated vesicle size and maturation by directly sensing and driving membrane curvature. *Dev Cell* **33**, 163-175, doi:10.1016/j.devcel.2015.03.002 (2015).
46 Martyna, A. *et al.* Membrane remodeling by the M2 amphipathic helix drives influenza virus membrane scission. *Sci Rep* **7**, 44695, doi:10.1038/srep44695 (2017).
47 Prakashchand, D. D. & Mondal, J. Conformational Reorganization of Apolipoprotein E Triggered by Phospholipid Assembly. *J Phys Chem B* **125**, 5285-5295, doi:10.1021/acs.jpcb.1c03011 (2021).
48 Torella, J. P., Holden, S. J., Santoso, Y., Hohlbein, J. & Kapanidis, A. N. Identifying Molecular Dynamics in Single-Molecule FRET Experiments with Burst Variance Analysis. *Biophys J* **100**, 1568-1577, doi:10.1016/j.bpj.2011.01.066 (2011).
49 Nettels, D., Hoffmann, A. & Schuler, B. Unfolded protein and peptide dynamics investigated with single-molecule FRET and correlation spectroscopy from picoseconds to seconds. *J Phys Chem B* **112**, 6137-6146, doi:10.1021/jp076971j (2008).
50 Zimmerman, M. I. & Bowman, G. R. FAST Conformational Searches by Balancing Exploration/Exploitation Trade-Offs. *J Chem Theory Comput* **11**, 5747-5757, doi:10.1021/acs.jctc.5b00737 (2015).

51 Mondal, T. *et al.* ApoE: In Vitro Studies of a Small Molecule Effector. *Biochemistry* **55**, 2613-2621, doi:10.1021/acs.biochem.6b00324 (2016).
52 Zosel, F., Haenni, D., Soranno, A., Nettels, D. & Schuler, B. Combining short- and long-range fluorescence reporters with simulations to explore the intramolecular dynamics of an intrinsically disordered protein. *Journal of Chemical Physics* **147**, doi:Artn 152708
10.1063/1.4992800 (2017).
53 Haenni, D., Zosel, F., Reymond, L., Nettels, D. & Schuler, B. Intramolecular distances and dynamics from the combined photon statistics of single-molecule FRET and photoinduced electron transfer. *J Phys Chem B* **117**, 13015-13028, doi:10.1021/jp402352s (2013).
54 Cubuk, J. *et al.* The SARS-CoV-2 nucleocapsid protein is dynamic, disordered, and phase separates with RNA. *Nat Commun* **12**, 1936, doi:10.1038/s41467-021-21953-3 (2021).
55 Holmstrom, E. D. *et al.* Accurate Transfer Efficiencies, Distance Distributions, and Ensembles of Unfolded and Intrinsically Disordered Proteins From Single-Molecule FRET. *Methods Enzymol* **611**, 287-325, doi:10.1016/bs.mie.2018.09.030 (2018).
56 Periasamy, N., Doraiswamy, S., Maiya, G. B. & Venkataraman, B. Diffusion Controlled Reactions - Fluorescence Quenching of Cationic Dyes by Charged Quenchers. *Journal of Chemical Physics* **88**, 1638-1651, doi:Doi 10.1063/1.454143 (1988).
57 Szabelski, M. *et al.* Evaluation of instrument response functions for lifetime imaging detectors using quenched Rose Bengal solutions. *Chem Phys Lett* **471**, 153-159, doi:10.1016/j.cplett.2009.02.001 (2009).
58 Johansson, L. B. A. Limiting Fluorescence Anisotropies of Perylene and Xanthene Derivatives. *J Chem Soc Faraday T* **86**, 2103-2107, doi:DOI 10.1039/ft9908602103 (1990).
59 Lakowicz, J. R. *Principles of fluorescence spectroscopy*. 3rd edn, (Springer, 2006).
60 Koshioka, M., Sasaki, K. & Masuhara, H. Time-Dependent Fluorescence Depolarization Analysis in 3-Dimensional Microspectroscopy. *Appl Spectrosc* **49**, 224-228, doi:Doi 10.1366/0003702953963652 (1995).
61 Sindbert, S. *et al.* Accurate distance determination of nucleic acids via Forster resonance energy transfer: implications of dye linker length and rigidity. *J Am Chem Soc* **133**, 2463-2480, doi:10.1021/ja105725e (2011).
62 Schuler, B., Soranno, A., Hofmann, H. & Nettels, D. Single-Molecule FRET Spectroscopy and the Polymer Physics of Unfolded and Intrinsically Disordered Proteins. *Annual Review of Biophysics, Vol 45* **45**, 207-231, doi:10.1146/annurev-biophys-062215-010915 (2016).
63 Ivanov, V., Li, M. & Mizuuchi, K. Impact of emission anisotropy on fluorescence spectroscopy and FRET distance measurements. *Biophys J* **97**, 922-929, doi:10.1016/j.bpj.2009.05.025 (2009).
64 Muller-Spath, S. *et al.* Charge interactions can dominate the dimensions of intrinsically disordered proteins. *P Natl Acad Sci USA* **107**, 14609-14614, doi:10.1073/pnas.1001743107 (2010).
65 Soranno, A. *et al.* Quantifying internal friction in unfolded and intrinsically disordered proteins with single-molecule spectroscopy. *P Natl Acad Sci USA* **109**, 17800-17806, doi:10.1073/pnas.1117368109 (2012).

66 Duan, Y. *et al.* A point-charge force field for molecular mechanics simulations of proteins based on condensed-phase quantum mechanical calculations. *J Comput Chem* **24**, 1999-2012, doi:10.1002/jcc.10349 (2003).
67 Jorgensen, W. L., Chandrasekhar, J., Madura, J. D., Impey, R. W. & Klein, M. L. Comparison of Simple Potential Functions for Simulating Liquid Water. *Journal of Chemical Physics* **79**, 926-935, doi:Doi 10.1063/1.445869 (1983).
68 Hess, B. P-LINCS: A parallel linear constraint solver for molecular simulation. *J Chem Theory Comput* **4**, 116-122, doi:10.1021/ct700200b (2008).
69 Bussi, G., Donadio, D. & Parrinello, M. Canonical sampling through velocity rescaling. *Journal of Chemical Physics* **126**, doi:Artn 014101
10.1063/1.2408420 (2007).
70 Abraham, M. J. *et al.* GROMACS: High performance molecular simulations through multi-level parallelism from laptops to supercomputers. *SoftwareX* **1-2**, 19-25, doi:https://doi.org/10.1016/j.softx.2015.06.001 (2015).
71 Zimmerman, M. I., Porter, J. R., Sun, X., Silva, R. R. & Bowman, G. R. Choice of Adaptive Sampling Strategy Impacts State Discovery, Transition Probabilities, and the Apparent Mechanism of Conformational Changes. *J Chem Theory Comput* **14**, 5459-5475, doi:10.1021/acs.jctc.8b00500 (2018).
72 Porter, J. R., Zimmerman, M. I. & Bowman, G. R. Enspara: Modeling molecular ensembles with scalable data structures and parallel computing. *J Chem Phys* **150**, 044108, doi:10.1063/1.5063794 (2019).
73 Fiser, A., Do, R. K. & Sali, A. Modeling of loops in protein structures. *Protein Sci* **9**, 1753-1773, doi:10.1110/ps.9.9.1753 (2000).
74 Eastman, P. *et al.* OpenMM 7: Rapid development of high performance algorithms for molecular dynamics. *PLoS Comput Biol* **13**, e1005659, doi:10.1371/journal.pcbi.1005659 (2017).
75 Borgia, A. *et al.* Extreme disorder in an ultrahigh-affinity protein complex. *Nature* **555**, 61-+, doi:10.1038/nature25762 (2018).
76 Piana, S., Donchev, A. G., Robustelli, P. & Shaw, D. E. Water Dispersion Interactions Strongly Influence Simulated Structural Properties of Disordered Protein States. *J Phys Chem B* **119**, 5113-5123, doi:10.1021/jp508971m (2015).
77 Hofmann, H. *et al.* Polymer scaling laws of unfolded and intrinsically disordered proteins quantified with single-molecule spectroscopy. *P Natl Acad Sci USA* **109**, 16155-16160, doi:10.1073/pnas.1207719109 (2012).
